# Eight new resin glycosides, calyhedins XXIV–XXXI, from the rhizomes of *Calystegia hederacea*

**DOI:** 10.1007/s11418-025-01919-1

**Published:** 2025-06-01

**Authors:** Masateru Ono, Yosuke Matsuoka, Ryota Arakawa, Hiroyuki Shimadzu, Takumi Nagasawa, Hirotaka Nishikawa, Shin Yasuda, Hiroyuki Miyashita, Kazumi Yokomizo, Hitoshi Yoshimitsu, Ryota Tsuchihasi, Masafumi Okawa, Junei Kinjo

**Affiliations:** 1 School of Agriculture, Tokai University, 871-12 Sugido, Mashiki-cho, Kamimashiki-Gun, Kumamoto, 861-2205 Japan; 2 Faculty of Pharmaceutical Sciences, Sojo University, 4-22-1 Ikeda, Nishi-ku, Kumamoto, 860-0082 Japan; 3 Faculty of Pharmaceutical Sciences, Fukuoka University, 8-19-1 Nanakuma, Jonan-ku, Fukuoka, 814-0180 Japan

**Keywords:** Resin glycoside, Jalapin, *Calystegia hederacea*, Calyhedin, Cytotoxic activity, Antiviral activity

## Abstract

**Abstract:**

Eight new resin glycosides, named calyhedins XXIV (**1**)–XXXI (**8**), were isolated from the rhizomes of *Calystegia hederacea* Wall. (Convolvulaceae), along with six known calyhedins: calyhedins II (**9**), III (**10**), IV (**11**), V (**12**), VIII (**13**), and XII (**14**). Their structures were determined from the spectroscopic data. Compounds **1**–**7** were hexa- or hepta-glycosides with macrolactone structures (jalapins), and their sugar moieties were partially acylated by five organic acids, including 2*S*-methylbutyric, (*E*)-2-methylbut-2-enoic, and 2*R*-methyl-3*R*-hydroxybutyric acids. Compounds **1**–**7** were macrolactones with 22- (**1**, **7**), 23- (**2**), 27- (**3**–**5**), or 28- (**6**) membered rings. Compound **8** was identified as an acylated glycosidic acid methyl ester, corresponding to the compound in which the lactone ring of **1** was cleaved and subsequently methylated. In addition, the cytotoxic activities of **1**–**8**, **11**, **12**, and **14** against HL-60 human promyelocytic leukemia cells and the antiviral activities of **1**–**14** and 11 previously isolated calyhedins against herpes simplex virus type 1 (HSV-1) were evaluated. Except for **1**, the other ten tested compounds exhibited cytotoxicity against HL-60 cells, with the IC_50_ values of **3**, **4**, **6**, **7**, **11**, and **12** being closer to or rather lower than that of the positive control cisplatin. Additionally, all tested compounds demonstrated anti-HSV-1 activity, with seven compounds, i.e., **3**, **6**, **9**, **10**, **22**, **23**, and **24**, having EC_50_ values lower than or comparable to that of the positive control acyclovir.

**Graphical abstract:**

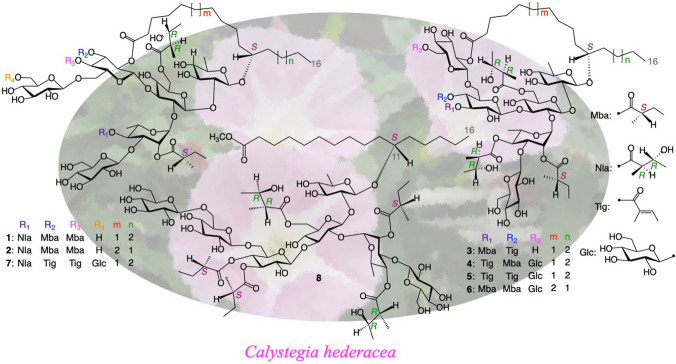

**Supplementary Information:**

The online version contains supplementary material available at 10.1007/s11418-025-01919-1.

## Introduction

The resin glycosides are widespread in plants belonging to the Convolvulaceae family. Within this botanical family, traditional crude drugs such as Rhizoma Jalapae (the root of *Ipomoea purga* (Wender) Hayne), Rhizoma Jalapae Braziliensis (the root of *Operculina macrocarpa* (L.) Urban, syn *I. operculata* (Gomes) Mart.), Orizaba Jalap Tuber (the tuber of *I. orizabensis* (Pelletan) Lebed.), and Pharbitidis Semen (the seeds of *Pharbitis nil* Choisy) exhibit laxative properties attributed to these compounds [1]. Chemically, the resin glycosides are hydroxyl fatty acid oligoglycosides (glycosidic acids) characterized by a core structure in which organic acids acylate the hydroxyl groups in the sugar moiety. The resin glycosides are broadly classified into jalapins, which have an intramolecular cyclic ester structure, and convolvulins, which contain a free carboxyl group on an aglycone [1]. Isolated resin glycosides have demonstrated diverse biological activities, including antibacterial [2], ionophoric [2], anti-inflammatory [3], antiviral [4, 5], and multidrug-resistance-modulating [6] properties, as well as cytotoxicity against cancer cells [2, 5, 7]. Owing to their complex structures and high molecular weights, resin glycosides have seldom been isolated or structurally characterized, and studies on their biological activities remain limited. This has prompted continued interest in the structural diversity and potential biological applications of resin glycosides derived from Convolvulaceae plants.

*Calystegia* species are perennial vines belonging to the Convolvulaceae family, which comprises approximately 25 species distributed across warm to temperate regions worldwide [8]. Recently, nine jalpins have been isolated from the aerial parts of *C. sepium*, and their structures have been elucidated [9, 10]. Furthermore, seven new glycosidic acids and two known organic acids have been identified as components of the resin glycoside fraction from the leaves and stems of *C. japonica*, have been reported [11].

*C. hederacea* Wall. is a perennial herbaceous vine that grows widely throughout India and Eastern Asia. All parts of the plant are used to treat menoxenia, gonorrhea, and other conditions [8]. We previously reported that the crude resin glycoside fraction of the whole plant of *C. hederacea* is composed of four monosaccharides (d-glucose, d-fucose, d-quinovose, and l-rhamnose), three organic acids [2*S*-methylbutyric, (*E*)-2-methylbut-2-enoic (tiglic), and 2*R*-methyl-3*R*-hydroxybutyric (2*R*,3*R*-nilic) acids], and two hydroxyl fatty acids [11*S*-hydroxyhexadecanoic (jalapinolic) and 12*S*-hydroxyhexadecanoic acids]. The absolute configurations of the monosaccharide components were determined using HPLC analysis of their thiocarbamoyl-thiazolidine derivatives. In contrast, the absolute configurations of 2-methylbutyric and nilic acids were elucidated by comparing the specific rotations of their *p*-bromophenacyl esters and ^1^H-NMR spectral data for the (–)-α-methoxy-α-trifluoromethylphenylacetic acid (MTPA) ester of *p*-bromophenacyl nilate with those of authentic samples. Furthermore, the absolute configurations of the jalapinolic and 12-hydroxyhexadecanoic acids were determined based on the ^1^H-NMR spectra of the MTPA esters of the methyl derivatives [12]. Additionally, six new (calyhedic acids A–F) and two known glycosidic acids (calysolic acids A and C) have been reported [12, 13]. Moreover, we have previously described the isolation and structural elucidation of 22 new genuine resin glycosides with macrolactone structures (jalapins) [1] (calyhedins I–XIV and XVI–XXIII) from the rhizomes of *C. hederacea* and a new genuine resin glycoside (calyhedin XV) from its leaves and stems [7, 14–16]. These compounds can be classified into two aglycone types, 11*S*-jalapinolic acid and 12*S*-hydroxyhexadecanoic acid. Both aglycones could be distinguished using methylene carbon signals in the ^13^C-NMR spectrum, especially the signal corresponding to C-14 [7]. In the course of our studies on the resin glycosides of this plant, the present report describes the isolation and structural elucidation of eight new and six known resin glycosides from the rhizomes of *C. hederacea*. Additionally, except for two compounds that could not be obtained in sufficient amounts for in vitro assays, the cytotoxicity of the compounds isolated in this study was evaluated in HL-60 human promyelocytic leukemia cells. Furthermore, the antiviral activities of 25 resin glycosides from *C. hederacea*, including the compounds isolated in this study and in previous reports, were assessed against herpes simplex virus type 1 (HSV-1).

## Results and discussion

Air-dried rhizomes of *C. hederacea* were extracted with methanol (MeOH). The extract was subjected to Diaion HP20, silica gel, octadecylsilyl (ODS) column chromatography, and HPLC on ODS and naphthylethyl group bonded silica (π-NAP) columns, yielding 14 compounds (**1**–**14**).

**Fig. 1:**
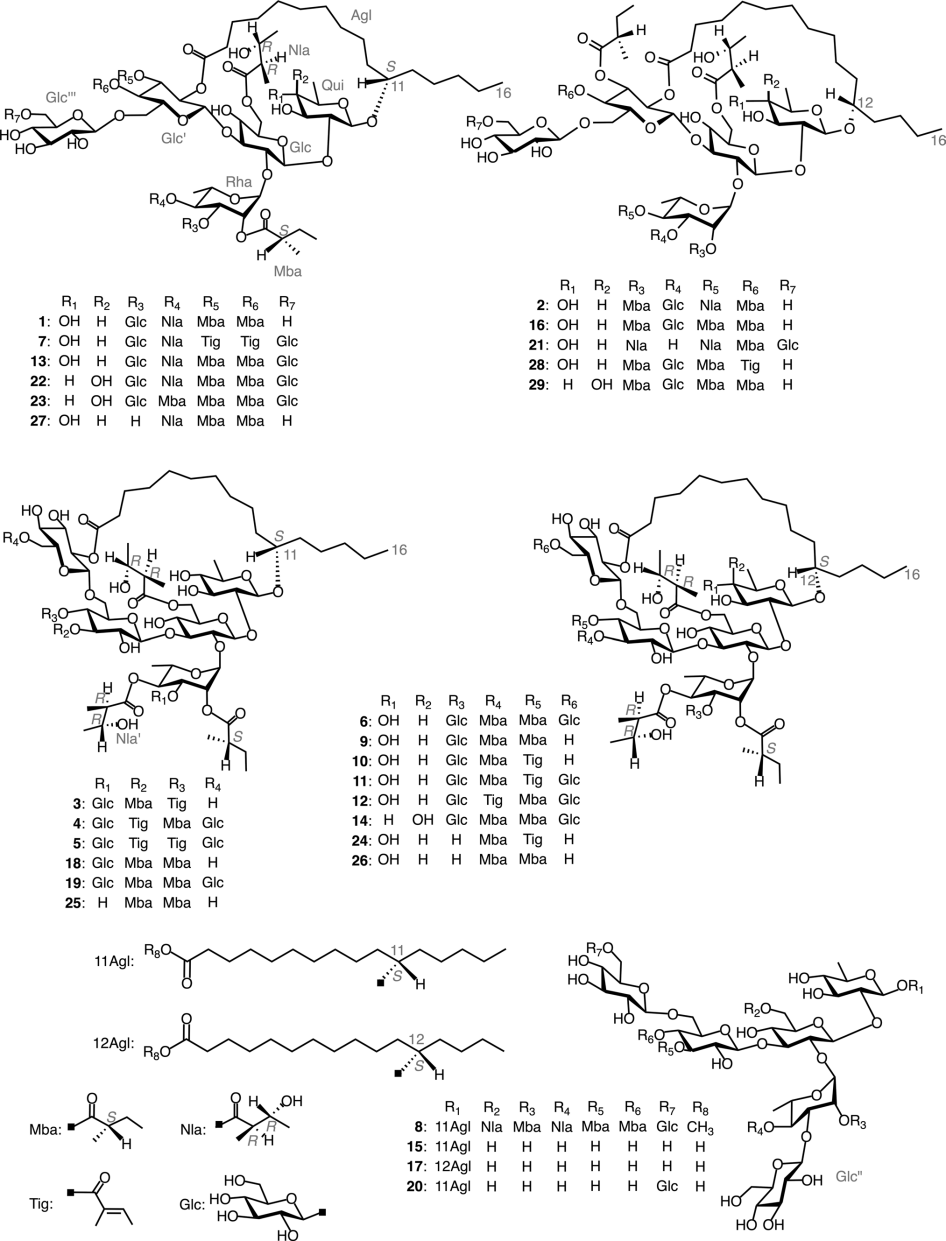
Structures of **1**–**29**

Compounds **9**–**14** were identified as calyhedins II (**9**) [7], III (**10**) [7], IV (**11**) [7], V (**12**) [7], VIII (**13**) [14], and XII (**14**) [15] by comparing their physical and spectral data with reported values (Fig. 1).

**Fig. 2:**
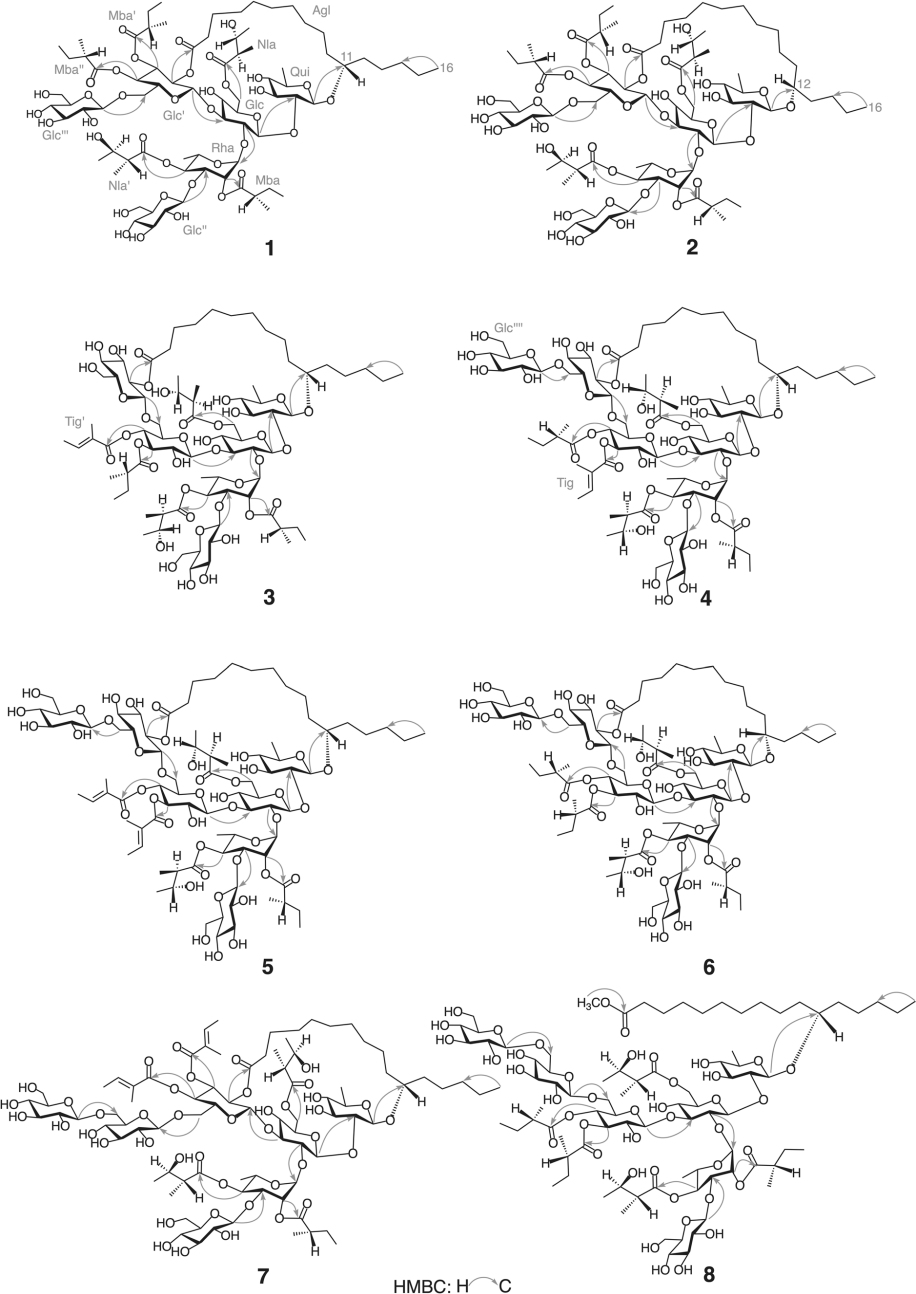
Key HMBC correlations observed for **1**–**8** (in pyridine-*d*_5_, 600 MHz)

**Fig. 3:**
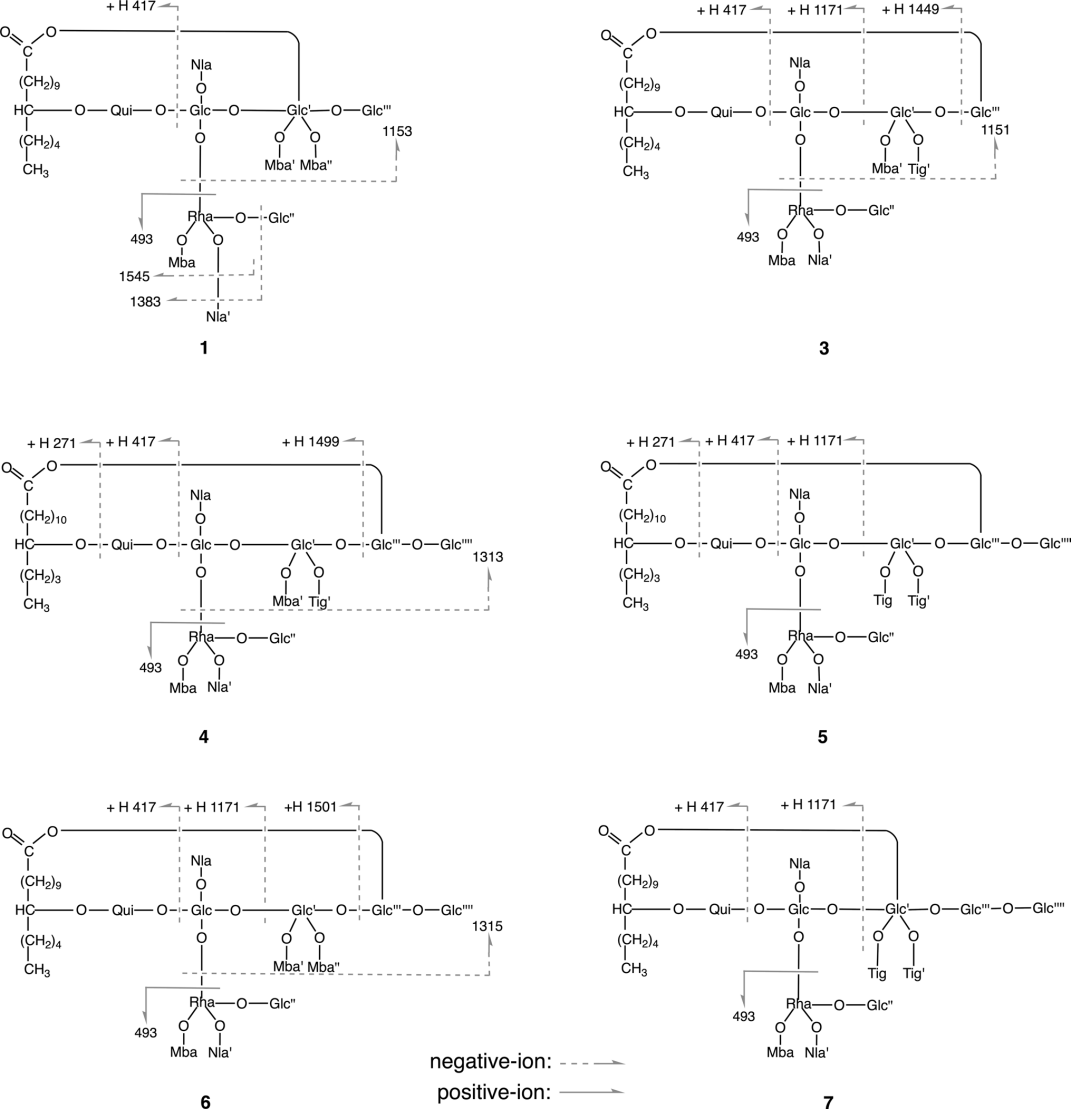
Fragment ions observed in the negative-ion and positive-ion FAB-MS of **1** and **3**–**7**

Compound **1** (calyhedin XXIV) was obtained as an amorphous powder and exhibited an [M + Na]^+^ ion peak at *m/z* 1669 in positive-ion FAB-MS and an [M–H]^–^ ion peak at *m/z* 1645 in negative-ion FAB-MS, indicating an exact mass of 1646. High-resolution (HR)-positive-ion FAB-MS revealed that the molecular formula of **1** is C_77_H_130_O_37_. The ^1^H-NMR spectrum of **1** exhibited signals attributable to three H-2 [$\delta$ 2.58 (1H, ddq, *J* = 7.0, 7.0, 7.0 Hz), 2.35 (1H, ddq, *J* = 7.0, 7.0, 7.0 Hz), 2.24 (1H, ddq, *J* = 7.0, 7.0, 7.0 Hz)] of 2-methylbutyryl residues, two H-2 [$\delta$3.12 (1H, dq, *J* = 7.0, 7.0 Hz), 2.83 (1H, dq, *J* = 7.0, 7.0 Hz)] of niloyl residues, four primary methyl groups [$\delta$0.86 (3H, t, *J* = 7.0 Hz), 0.84 (3H, dd, *J* = 7.5, 7.5 Hz), 0.82 (3H, dd, *J* = 7.5, 7.5 Hz) 0.78 (3H, dd, *J* = 7.5, 7.5 Hz)], nine secondary methyl groups [$\delta$1.77 (3H, d, *J* = 6.0 Hz), 1.61 (3H, d, *J* = 6.0 Hz), 1.45 (3H, d, *J* = 7.0 Hz), 1.41 (3H, d, *J* = 6.5 Hz), 1.35 (3H, d, *J* = 6.5 Hz), 1.28 (3H, d, *J* = 7.0 Hz), 1.26 (3H, d, *J* = 7.0 Hz), 1.10 (3H, d, *J* = 7.0 Hz), 1.05 (3H, d, *J* = 7.0 Hz)], and six anomeric protons [$\delta$5.78 (1H, s), 5.59 (1H, d, *J* = 7.5 Hz), 5.33 (1H, d, *J* = 8.0 Hz), 5.16 (1H, d, *J* = 8.0 Hz), 5.13 (1H, d, *J* = 8.0 Hz), 4.75 (1H, d, *J* = 8.0 Hz]. The ^13^C-NMR spectrum showed signals corresponding to six carboxyl carbons ($\delta$ 176.9, 175.7, 175.6, 175.5, 175.0, 172.7) and six anomeric carbons ($\delta$ 105.6, 104.8, 103.2, 101.9, 99.6, 97.2). These NMR signals were assigned on the basis of ^1^H-^1^H COSY, ^1^H-^1^H total correlation spectroscopy (TOCSY), HMQC, and HMBC spectra (Tables 1 and 2). The assigned spectral data were quite similar to those of **13**, except for the disappearance of signals attributable to 1 mol of β-glucopyranosyl residue and an upfield-shift (7.6 ppm) of the chemical shift of signal corresponding to C-6 of the fourth glucosyl residue (Glc''') in **1**. This observed shift was ascribed to the cleavage of glycosidic linkage at C-6 of Glc''' [17, 18]. In particular, the ^13^C-NMR data for the aglycone moiety (Agl), including C-14 ($\delta$ 32.3), were nearly identical. Thus, the aglycone of **1** was considered to be jalapinolic acid. In the HMBC spectrum of **1**, key cross-peaks were observed between H-1 of quinovosyl residue (Qui) and C-11 of Agl, H-1 of the first glucosyl residue (Glc) and C-2 of Qui, H-2 of Glc and C-1 of the rhamnosyl residue (Rha), H-1 of the second glucosyl residue (Glc') and C-3 of Glc, H-1 of the third glucosyl unit (Glc'') and C-3 of Rha, and H-1 of Glc''' and C-6 of Glc'. These data and previous studies on the component organic acids and glycosidic acids of the crude resin glycoside fraction of whole plants of *C. hederacea* [12] indicated that **1** is composed of 1 mol of calysolic acid C (**15**) [12, 19], 3 mol of 2*S*-methylbutyryic acid, and 2 mol of 2*R*,3*R*-nilic acid. Further, the carboxyl group of the aglycone, 11*S*-jalapinolic acid, of **1** is linked intramolecularly with a hydroxy group of the sugar moiety to form a macrocyclic ester structure since the ^1^H-NMR signals due to H_2_-2 of the Agl of **1** are nonequivalent at $\delta$ 2.64 (1H) and $\delta$ 2.62 (1H), whereas **15** exhibits the equivalent signal due to H_2_-2 of Agl at $\delta$ 2.52 (2H, t, *J* = 7.5 Hz) [12]. When comparing the chemical shifts of the ^1^H-NMR signals of **1** and **15** [12], remarkable downfield shifts (Δ$\delta$ = $\delta$**1**–$\delta$**15**) due to acylation were observed for signals assignable to H-6a (Δ$\delta$ = 0.27) and H-6b (Δ$\delta$ = 0.46) of Glc, H-2 (Δ$\delta$ = 1.26) and H-4 (Δ$\delta$ = 1.57) of Rha, and H-2 (Δ$\delta$ = 1.48), H-3 (Δ$\delta$ = 1.74), and H-4 (Δ$\delta$ = 1.34) of Glc'. Therefore, ester linkages could be located at C-6 of Glc, C-2 and C-4 of Rha, and C-2, C-3, and C-4 of Glc'. Using the HMBC spectrum of **1**, the sites of each ester linkage of the organic acid residues and Agl were investigated. In the HMBC spectrum, key cross-peaks were observed between H-6a and H-6b of Glc and C-1 of the first niloyl residue (Nla), H-2 of Rha and C-1 of the first 2-methylbutyryl residue (Mba), H-4 of Rha and C-1 of the second niloyl residue (Nla') or C-1 of the third 2-methylbutyryl residue (Mba"), H-2 of Glc' and C-1 of Agl, H-3 of Glc' and C-1 of the second 2-methylbutyryl residue (Mba'), and H-4 of Glc' and C-1 of Mba" or C-1 of Nla' (Fig. 2). These data indicated that Nla, Mba, Agl, and Mba' were attached to C-6 of Glc, C-2 of Rha, C-2 of Glc', and C-3 of Glc'. However, the counterparts of H-4 of Rha and H-4 of Glc' could not be identified because the ^13^C-NMR signals corresponding to C-1 of Nla' and C-1 of Mba" appeared at almost the same chemical shifts. Finally, the ester linkages of Nla' and Mba" were confirmed by FAB-MS analysis of **1**. In negative-ion FAB-MS, a fragment ion peak was observed at *m/z* 1153 [M–H–84 (2-methylbutyryl unit)–100 (niloyl unit)–146 (6-deoxyhexosyl unit)–162 (hexosyl unit)] ^–^ (Fig. 3). In addition, the positive-ion FAB-MS of **1** exhibited a fragment ion peak at *m/z* 493, which was assigned to the fragment ion of the (2-*O*-2-methylbutyryl,3-*O*-glucosyl,4-*O*-niloyl)-rhamnosyl unit [7]. These MS data suggested that Nla' and Mba" were located at Rha and Glc', respectively. The coupling constants of the signals due to the anomeric and methine protons of the sugar moiety indicated that the conformations of the quinovopyranosyl and glucopyranosyl residues were ^4^C_1_ and that of the rhamnopyranosyl residue was ^1^C_4_. Accordingly, **1** was proposed as 11*S*-jalapinolic acid 11-*O*-β-d-glucopyranosyl-(1 → 3)-*O*-(2-*O*-2*S*-methylbutyryl-4-*O*-2*R*,3*R*-niloyl)-α-l-rhamnopyranosyl-(1 → 2)-[*O*-β-d-glucopyranosyl-(1 → 6)-*O*-(3,4-di-*O*-2*S*-methylbutyryl)-β-d-glucopyranosyl-(1 → 3)]-*O*-(6-*O*-2*R*,3*R*-niloyl)-β-d-glucopyranosyl-(1 → 2)-β-d-quinovopyranoside, intramolecular 1,2''''-ester (Fig. 1).

**Table 1:** ^1^H-NMR spectral data for **1**–**3** (in pyridine-*d*_5_, 600 MHz)

Position	**1**	**2**	**3**
Qui-1	4.75 d(8.0)	4.81 d(8.0)	4.84 d(7.5)
2	4.09 dd(8.0, 9.0)	4.12 dd(8.0, 9.0)	4.23 dd(7.5, 9.0)
3	4.44 dd(9.0, 9.0)	4.47^a^	4.49 dd(9.0, 9.0)
4	3.61 dd(9.0, 9.0)	3.61 dd(9.0, 9.0)	3.62 dd(9.0, 9.0)
5	3.92 dq(9.0, 6.0)	3.98^a^	3.97^a^
6	1.61 d(6.0)	1.61 d(6.0)	1.62 d(6.0)
Glc-1	5.59 d(7.5)	5.75 d(7.5)	5.72 d(8.0)
2	4.10 dd(7.5, 9.0)	4.08^a^	4.09 dd(8.0, 8.5)
3	4.56 dd(9.0, 9.0)	4.62 dd(9.0, 9.0)	3.71 ddd(3.5, 8.5, 8.5)^b^
4	3.80 dd(9.0, 9.0)	3.78^a^	3.83^a^
5	3.99^a^	4.09^a^	3.77^a^
6a	4.72 dd(2.0, 11.0)	4.73 d(11.0)	4.88 br d(11.5)
6b	4.65 dd(6.0, 11.0)	4.64 dd(6.0, 11.0)	4.70 dd(7.5, 11.5)
Rha-1	5.78 s	5.80 s	6.23 s
2	6.09 d(3.5)	6.04 d(3.5)	6.09 d(3.0)
3	5.18 dd(3.5, 10.0)	5.23^a^	5.18^a^
4	5.90 dd(10.0, 10.0)	5.91dd(10.0, 10.0)	5.90 dd(10.0, 10.0)
5	5.38 dq(10.0, 6.0)	5.33 dq(10.0, 6.0)	5.27 dd(10.0, 6.0)
6	1.77 d(6.0)	1.75 d(6.0)	1.79 d(6.0)
Glc'-1	5.33 d(8.0)	5.30 d(8.0)	4.90 d(7.5)
2	5.46 dd(8.0, 9.5)	5.41 dd(8.0, 9.0)	3.94 dd(7.5, 9.5)
3	5.90 dd(9.5, 9.5)	5.84 dd(9.0, 9.0)	5.70 dd(9.5, 9.5)
4	5.31 dd(9.5, 9.5)	5.25 dd(9.0, 9.0)	5.61 dd(9.5, 9.5)
5	4.35^a^	4.21^a^	4.01 ddd(3.0, 4.5, 9.5)
6a	4.27 br d(12.5)	4.32 d(12.5)	4.17 dd(3.0, 12.5)
6b	4.10^a^	4.08^a^	4.07 dd(4.5, 12.5)
Glc"-1	5.13 d(8.0)	5.23 d(8.0)	5.18 d(8.0)
2	3.88^a^	3.90 dd(8.0, 9.0)	3.89^a^
3	4.03 dd(9.0, 9.0)	4.09^a^	4.09 dd(8.5, 8.5)
4	3.88^a^	3.84 dd(9.0, 9.0)	3.89^a^
5	4.04^a^	4.11^a^	4.16^a^
6a	4.45^a^	4.35^a^	4.54 br d(10.0)
6b	4.04^a^	4.00^a^	4.16^a^
Glc'''-1	5.16 d(8.0)	5.23 d(8.0)	5.03 d(7.5)
2	3.98^a^	3.97 dd(8.0, 9.0)	5.50 dd(7.5, 9.5)
3	4.30 dd(9.0, 9.0)	4.32 dd(9.0, 9.0)	4.32 dd(9.5, 9.5)
4	4.22 dd(9.0, 9.0)	4.21^a^	4.22 dd(9.5, 9.5)
5	3.98^a^	4.00^a^	3.83^a^
6a	4.48 br d(10.5)	4.48 br d(10.5)	4.42 br d(11.0)
6b	4.37^a^	4.35^a^	4.29 dd(5.0, 11.0)
Agl-2	2.64^a^	2.68 m	2.56^a^
2	2.62^a^	2.56 m	2.41^a^
10a	1.67^a^		1.71^a^
10b	1.57^a^		1.62^a^
11a	3.65 m	1.70^a^	3.77^a^
11b		1.55^a^	
12a	1.86^a^	3.78^a^	1.86^a^
12b	1.60^a^		1.63^a^
13a	1.44^a^	1.80^a^	1.48^a^
13b		1.64^a^	
14a	1.29^a^	1.54^a^	1.31^a^
14b	1.24^a^	1.48^a^	1.23^a^
15	1.29^a^	1.34^a^	1.31^a^
16	0.86 t(7.0)	0.90 t(7.5)	0.87 t(7.0)
Nla-2	2.83 dq(7.0, 7.0)	2.80 dq(7.0, 7.0)	2.82 dq(7.0, 7.0)
3	4.39^a^	4.36^a^	4.37 m
4	1.35 d(6.5)	1.35 d(6.5)	1.36 d(6.5)
5	1.26 d(7.0)	1.23 d(7.0)	1.34 d(7.0)
Nla'-2	3.12 dq(7.0, 7.0)	3.13 dq(7.0, 7.0)	3.10 dq(7.0, 7.0)
3	4.43^a^	4.45^a^	4.43^a^
4	1.41 d(6.5)	1.43 d(6.5)	1.44 d(6.5)
5	1.45 d(7.0)	1.48 d(7.0)	1.48 d(7.0)
Mba-2	2.58 ddq(7.0, 7.0, 7.0)	2.58 ddq(7.0, 7.0, 7.0)	2.38 ddq(7.0, 7.0, 7.0)
3a	1.83^a^	1.82 m	1.71 m
3b	1.53^a^	1.51 m	1.43^a^
4	0.82 dd(7.5, 7.5)	0.83 dd(7.5, 7.5)	0.79 dd(7.5, 7.5)
5	1.28 d(7.0)	1.30 d(7.0)	1.19 d(7.0)
Mba'-2	2.35 ddq(7.0, 7.0, 7.0)	2.35 ddq(7.0, 7.0, 7.0)	2.50 ddq(7.0, 7.0, 7.0)
3a	1.68^a^	1.69 m	1.78^a^
3b	1.40^a^	1.42^a^	1.46^a^
4	0.84 dd(7.5, 7.5)	0.85 dd(7.5, 7.5)	0.92 dd(7.5, 7.5)
5	1.10 d(7.0)	1.09 d(7.0)	1.16 d(7.0)
Mba"-2	2.24 ddq(7.0, 7.0, 7.0)	2.23 ddq(7.0, 7.0, 7.0)	
3a	1.65^a^	1.61^a^	
3b	1.31^a^	1.30^a^	
4	0.78 dd(7.5, 7.5)	0.77 dd(7.5, 7.5)	
5	1.05 d(7.0)	1.04 d(7.0)	
Tig’-3			6.95 qq(1.0, 7.0)
4			1.61 d(7.0)
5			1.84 br s

$\delta$in ppm from TMS [coupling constants (J) in Hz are given in parentheses]

*Qui* quinovopyranosyl, *Fuc* fucopyranosyl, *Glc* glucopyranosyl, *Rha* rhamnopyranosyl, *Agl* Aglycone moiety, *Nla* niloyl, *Mba* 2-methylbutyryl, *Tig* tigloyl

^a^Signals were overlapped with other signals

^b^*J* = 3.5 Hz arose from coupling of hydroxyl proton ($\delta$ 7.49)

**Table 2:** ^13^C-NMR spectral data of compounds **1**–**8** (150 MHz, in pyridine-*d*_5_)

Position	**1**	**2**	**3**	**4**	**5**	**6**	**7**	**8**
Qui-1	103.2	102.3	103.1	103.1	103.1	103.4	103.3	102.0
2	81.2	80.8	79.7	79.6	79.7	79.6	81.1	79.8
3	78.9	79.4	79.4	79.4	79.5	79.5	78.9	79.4
4	77.2	77.1	77.2	77.2	77.2	77.1	77.2	77.2
5	71.9	72.1	72.4	72.3	72.4	72.4	71.8	72.3
6	18.4	18.5	18.5	18.5	18.5	18.5	18.4	18.5
Glc-1	101.9	101.8	101.1	101.1	101.1	101.0	101.9	101.2
2	76.2	74.2	74.8	74.9	74.9	74.8	76.4	74.8
3	84.6	84.5	89.6	89.5	89.7	88.7	84.4	88.9
4	70.1	70.1	70.8	70.7	70.8	71.3	70.9	70.5
5	73.9	74.6	73.9	73.9	74.0	73.9	73.9	73.8
6	64.6	64.6	64.9	64.9	64.9	64.8	64.7	64.2
Rha-1	97.2	97.0	97.1	97.2	97.2	97.0	97.3	97.1
2	72.9	73.1	73.3	73.0	73.1	73.0	72.8	73.0
3	75.4	74.5	75.0	75.2	75.1	74.9	75.2	74.8
4	73.6	73.8	74.0	74.0	73.9	74.0	73.6	74.1
5	67.2	67.3	67.1	67.1	67.1	67.1	67.2	67.1
6	18.0	18.3	18.7	18.7	18.7	18.7	18.0	18.6
Glc'-1	99.6	99.6	103.9	103.8	104.1	103.4	99.8	103.6
2	72.2	72.2	72.5	72.8	72.9	72.5	72.4	72.4
3	73.2	73.2	75.6	76.3	76.4	76.0	73.7	75.2
4	70.4	70.3	69.3	68.6	69.3	68.4	70.2	69.8
5	74.3	75.5	73.4	73.0	73.3	73.2	74.5	73.8
6	68.4	68.1	66.1	66.1	66.7	65.8	68.8	68.7
Glc"-1	105.6	105.6	105.8	105.7	105.7	105.8	105.6	105.7
2	75.0	75.2	75.1	75.1	75.0	75.1	75.1	75.3
3	78.2	78.3	78.3	78.0	78.0	78.1	78.4	77.8
4	72.0	72.1	72.0	71.6	71.6	71.7	72.0	72.1
5	77.8	77.8	78.0	78.5	78.5	78.6	77.9	78.4
6	63.2	63.3	63.2	63.1	63.1	63.3	63.1	63.2
Glc'''-1	104.8	104.7	102.1	102.3	102.4	102.6	105.1	104.5
2	75.4	75.5	75.1	74.8	74.9	74.6	75.5	75.3
3	78.1	78.1	76.1	76.0	76.1	76.3	77.8	78.0
4	71.5	71.6	71.7	71.2	71.3	70.3	71.5	71.2
5	78.2	78.2	79.2	77.5	77.5	77.8	76.9	76.9
6	62.5	62.5	62.3	68.9	69.0	68.4	70.0	70.0
Glc''''-1				105.4	105.4	105.5	105.6	105.5
2				75.1	75.1	75.1	75.4	75.2
3				78.2	78.2	78.3	78.2	78.3
4				71.9	71.9	72.1	71.4	71.2
5				78.4	78.4	78.5	78.5	78.5
6				62.8	62.8	62.8	62.7	62.8
Agl-1	172.7	172.7	173.0	173.0	173.0	172.8	172.8	174.0
2	33.9	33.6	34.4	34.3	34.4	34.3	33.8	34.2
3	25.0	24.9	25.0	25.4	25.3	25.1	25.1	25.3
10	35.6		35.4	35.4	35.4		35.7	34.7
11	81.4	34.6	82.0	81.8	81.8	35.2	81.5	80.6
12	36.5	80.5	35.7	35.7	35.7	82.1	36.5	35.2
13	25.5	35.4	25.3	25.0	24.9	35.2	25.5	25.1
14	32.3	27.7	32.3	32.3	32.3	28.0	32.3	32.3
15	23.0	23.2	23.0	23.0	23.0	23.0	23.0	23.0
16	14.3	14.4	14.3	14.3	14.3	14.3	14.3	14.3
CH_2_	25.3	24.7	25.4	25.3	25.4	25.1	25.4	25.3
CH_2_	28.7	28.6	28.9	29.1	29.1	29.4	28.8	29.5
CH_2_	29.9	28.7	29.6	29.6	29.9	29.6	30.0	29.7
CH_2_	30.2	29.0	30.2	30.4	30.3	30.0	30.3	30.0
CH_2_	30.5	29.6	31.0	31.1	31.0	30.5	30.6	30.3
CH_2_	30.9	29.6	31.3	31.3	31.3	31.2	30.9	30.6
CH_2_		29.9				31.3		
OCH_3_								51.3
Nla-1	175.7	175.7	175.6	175.7	175.6	175.6	175.7	175.4
2	48.2	48.2	48.4	48.4	48.5	48.3	48.2	48.3
3	68.9	68.9	69.0	69.0	69.3	69.0	68.9	68.9
4	20.8	20.8	21.0	21.1	21.1	21.0	20.8	20.8
5	13.3	13.4	13.7	13.7	13.7	13.6	13.3	13.4
Nla'-1	175.5	175.6	175.5	175.5	175.5	175.5	175.7	175.4
2	49.4	49.4	49.4	49.4	49.4	49.4	49.4	49.3
3	69.9	69.8	69.7	69.7	69.7	69.7	69.9	69.6
4	21.6	21.7	21.6	21.6	21.6	21.6	21.6	21.5
5	14.1	14.0	14.0	14.0	14.0	14.0	14.2	13.9
Mba-1	176.9	176.8	176.9	176.7	176.7	176.6	176.8	176.6
2	41.1	41.0	41.2	41.2	41.2	41.0	41.0	41.0
3	26.9	26.6	26.6	26.7	26.7	26.6	26.9	26.6
4	11.5	11.5	11.9	11.5	11.5	11.6	11.5	11.6
5	16.5	16.5	16.8	16.6	16.6	16.8	16.2	16.8
Mba'-1	175.0	175.0	175.8	175.8		175.7		175.6
2	41.1	41.1	41.6	41.4		41.3		41.2
3	26.6	26.5	27.1	26.8		26.8		27.0
4	11.6	11.6	11.5	12.0		11.8		11.8
5	16.5	16.5	17.0	16.8		16.8		16.8
Mba"-1	175.6	175.5				175.7		175.7
2	40.8	40.8				41.2		41.2
3	26.6	26.8				27.1		26.6
4	11.6	11.6				12.0		11.8
5	16.3	16.3				16.6		16.6
Tig-1				167.2	167.3		166.6	
2				128.8	128.9		128.5	
3				137.9	137.6		138.0	
4				14.2	14.4		14.2	
5				12.3	12.3		12.2	
Tig'-1			167.2		167.3		167.0	
2			128.2		128.3		128.1	
3			139.4		139.2		139.1	
4			14.4		14.2		14.1	
5			12.2		12.3		12.1	

$\delta$in ppm from TMS

*Qui* quinovopyranosyl, *Fuc* fucopyranosyl, *Glc* glucopyranosyl, *Rha* rhamnopyranosyl, *Agl* Aglycone moiety, *Nla* niloyl, *Mba* 2-methylbutyryl, *Tig* tigloyl

Compound **2** (calyhedin XXV) was obtained as an amorphous powder, and its molecular formula was determined to be the same as that of **1** using HR-positive-ion FAB-MS. The ^1^H- and ^13^C-NMR spectra of **2** were nearly identical to those of **1**, except for slight differences in the signals due to Agl. NMR signals were assigned using the same two-dimensional (2D)-NMR techniques as those used for **1** (Tables 1 and 2). The ^13^C-NMR data of the Agl of **2** were almost the same as those of calyhedin XXI (**16**) [16], and acylation shifts were observed in the same signals as those of **1** in the ^1^H-NMR spectrum. These data suggested that **2** is a congener of **1** in which the glycosidic acid (**15**) component of **1** is replaced by calyhedic acid B (**17**) [12], which differs from **1** only in its aglycone. In the HMBC spectrum of **2**, key cross-peaks were detected between H-6a and H-6b of Glc and C-1 of Nla, H-2 of Rha and C-1 of Mba, H-4 of Rha and C-1 of Nla', H-2 of Glc' and C-1 of Agl, H-3 of Glc' and C-1 of Mba', and H-4 of Glc' and C-1 of Mba" (Fig. 2). In addition, the negative-ion and positive-ion FAB-MS of **2** exhibited the same fragment ion peaks as those of **1** at *m/z* 1153 and 493, respectively. Accordingly, **2** was defined as an isomer of **1**, in which the 11*S*-jalapinoyl residue in **1** was replaced by a 12*S*-hydroxyhexadecanoyl residue (Fig. 1).

Compound **3** (calyhedin XXVI) was obtained as an amorphous powder, and its exact mass was determined to be 1644, which is two mass units smaller than that of **1**, with an [M + Na]^+^ ion peak at *m/z* 1667 in positive-ion FAB-MS and an [M–H]^–^ ion peak at *m/z* 1643 in negative-ion FAB-MS. The molecular formula was determined to be C_77_H_128_O_37_ using HR-positive-ion FAB-MS. The ^1^H- and ^13^C-NMR spectra of **3** were similar to those of calyhedin I (**18**) [7], except for the appearance of signals due to the 1 mol tigloyl residue and the absence of signals due to the 1 mol of 2-methylbutyryl residue (Tables 1 and 2). Furthermore, acylation shifts were observed for the same signals as those of **18** in the ^1^H-NMR spectrum of **3**. In addition, the HMBC spectrum of **3** showed key cross-peaks between H-6a and H-6b of Glc and C-1 of Nla or C-1 of Nla', H-2 of Rha and C-1 of Mba, H-4 of Rha and C-1 of Nla' or C-1 of Nla, H-3 of Glc' and C-1 of Mba', H-4 of Glc' and C-1 of the tigloyl residue (Tig'), and H-2 of Glc''' and C-1 of Agl (Fig. 2). Furthermore, fragment ion peaks were detected at *m/z* 1171 [M–H–82 (tigloyl unit)–84–144 (162–H_2_O)–162]^–^ and 1151 in negative-ion FAB-MS and at *m/z* 493 in positive-ion FAB-MS (Fig. 3). Thus, **3** was defined as 11*S*-jalapinolic acid 11-*O*-β-d-glucopyranosyl-(1 → 3)-*O*-(2-*O*-2*S*-methylbutyryl,4-*O*-2*R*,3*R*-niloyl)-α-l-rhamnopyranosyl-(1 → 2)-[*O*-β-d-glucopyranosyl-(1 → 6)-*O*-(3-*O*-2*S*-methylbutyryl,4-*O*-tigloyl)-β-d-glucopyranosyl-(1 → 3)]-*O*-(6-*O*-2*R*,3*R*-niloyl)-β-d-glucopyranosyl-(1 → 2)-β-d-quinovopyranoside, intramolecular 1,2''''''-ester (Fig. 1).

Compound **4** (calyhedin XXVII) was obtained as an amorphous powder. Its exact mass was 1806, which is one hexosyl unit (162 mass units) larger than that of **3**, with an [M + Na]^+^ ion peak at *m/z* 1829 in positive-ion FAB-MS and an [M–H]^–^ ion peak at *m/z* 1805 in negative-ion FAB-MS. The molecular formula of **4** was determined to be C_83_H_138_O_42_ using HR-positive-ion FAB-MS. The ^1^H-NMR spectrum of **4** showed signals corresponding to two H-2 of 2-methylbutyryl residues, two H-2 of niloyl residues, one H-3 of tigloyl residue, three primary methyl groups, nine secondary methyl groups, and seven anomeric protons. The ^13^C-NMR spectrum showed signals corresponding to six carboxyl carbons and seven anomeric carbons. These signals were assigned using the same 2D-NMR spectroscopy used for **1**–**3** (Tables 2 and 3). The data were similar to those of **12**, except for the Agl, and the data of Agl were almost identical to those of **3**, indicating that the aglycone of **4** is 11*S*-jalapinolic acid. The HMBC spectrum of **4** indicated key cross-peaks between H-1 of Qui and C-11 of Agl, H-1 of Glc and C-2 of Qui, H-2 of Glc and C-1 of Rha, H-1 of Glc' and C-3 of Glc, H-3 of Rha and C-1 of Glc'', H-1 of Glc''' and C-6 of Glc', and H-1 of the fifth glucosyl residue (Glc'''') and C-6 of Glc''', Ha-6 and Hb-6 of Glc and C-1 of Nla, H-2 of Rha and C-1 of Mba, H-4 of Rha and C-1 of Nla', H-3 of Glc' and C-1 of the tigloyl residue (Tig), H-4 of Glc' and C-1 of Mba'', and H-2 of Glc''' and C-1 of Agl (Fig. 2). The negative- and positive-ion FAB-MS yielded fragment ion peaks at *m/z* 1313 and 493, respectively (Fig. 3). Accordingly, **4** was assigned as 11*S*-jalapinolic acid 11-*O*-β-d-glucopyranosyl-(1 → 6)-*O*-β-d-glucopyranosyl-(1 → 6)-*O*-(3-*O*-tigloyl,4-*O*-2*S*-methylbutyryl)-β-d-glucopyranosyl-(1 → 3)-[*O*-β-d-glucopyranosyl-(1 → 3)-*O*-(2-*O*-2*S*-methylbutyryl-4-*O*-2*R*,3*R*-niloyl)-α-l-rhamnopyranosyl-(1 → 2)]-*O*-(6-*O*-2*R*,3*R*-niloyl)-β-d-glucopyranosyl-(1 → 2)-β-d-quinovopyranoside, intramolecular 1,2''''''-ester (Fig. 1).

**Table 3:** ^1^H-NMR spectral data for **4**–**6** (in pyridine-*d*_5_, 600 MHz)

Position	**4**	**5**	**6**
Qui-1	4.84 d(7.5)	4.85 d(7.5)	4.84 d(8.0)
2	4.25^a^	4.24^a^	4.26^a^
3	4.49 dd(9.0, 9.0)	4.50 dd(9.0, 9.0)	4.51 dd(9.0, 9.0)
4	3.64 dd(9.0, 9.0)	3.64 dd(9.0, 9.0)	3.64 dd(9.0, 9.0)
5	3.97^a^	3.98^a^	4.00^a^
6	1.62 d(6.0)	1.62 d(6.0)	1.62 d(6.0)
Glc-1	5.72 d(7.5)	5.74 d(8.0)	5.77 d(7.5)
2	4.11 dd(7.5, 8.5)	4.12^a^	4.07 dd(7.5, 9.0)
3	3.72 dd(8.5, 8.5)	3.75 dd(8.5, 8.5)	3.62^a^
4	3.86 dd(8.5, 8.5)	3.88^a^	3.76^a^
5	3.78^a^	3.79^a^	3.76^a^
6a	4.89 br d(11.0)	4.91 br d(11.5)	4.85 br d(12.0)
6b	4.71^a^	4.73 dd(7.0, 11.5)	4.65 dd(6.0, 12.0)
Rha-1	6.30 d(1.5)	6.29 d(1.5)	6.31 d(1.0)
2	6.14 dd(1.5, 3.5)	6.14 dd(1.5, 3.0)	6.14 dd(1.0, 3.5)
3	5.17 dd(3.5, 10.0)	5.18 dd(3.0, 10.0)	5.21 dd(3.5, 10.0)
4	5.88 dd(10.0, 10.0)	5.88 dd(10.0, 10.0)	5.92 dd(10.0, 10.0)
5	5.26 dq(10.0, 6.0)	5.27 dq(10.0, 6.0)	5.25 dq(10.0, 6.0)
6	1.79 d(6.0)	1.79 d(6.0)	1.81 d(6.0)
Glc'-1	4.91 d(8.0)	4.96^a^	4.80 d(7.5)
2	3.98^a^	4.03 ddd(6.0, 7.5, 9.5)^b^	3.94^a^
3	5.73 dd(10.0, 10.0)	5.80 dd(9.5, 9.5)	5.62 dd(9.5, 9.5)
4	5.60 dd(10.0, 10.0)	5.65 dd(9.5, 9.5)	5.64 dd(9.5, 9.5)
5	3.97^a^	4.08^a^	3.95^a^
6a	4.22^a^	4.24^a^	4.31 d(11.0)
6b	4.04 dd(3.0, 12.5)	4.12^a^	4.11^a^
Glc"-1	5.17 d(8.0)	5.16 d(7.5)	5.23 d(8.0)
2	3.89^a^	3.89^a^	3.91 dd(8.0, 9.0)
3	4.09 dd(9.0, 9.0)	4.08^a^	4.11^a^
4	3.89^a^	3.88^a^	3.88 dd(9.0, 9.0)
5	4.12^a^	4.13^a^	4.23^a^
6a	4.51^a^	4.51 br d(11.0)	4.55^a^
6b	4.11^a^	4.13^a^	4.12^a^
Glc'''-1	4.90 d(8.0)	4.95^a^	4.85 d(8.0)
2	5.42 dd(8.0, 9.0)	5.43 dd(8.0, 9.5)	5.49 dd(8.0, 9.0)
3	4.24^a^	4.24^a^	4.25^a^
4	4.29 dd(9.0, 9.0)	4.24^a^	4.30 dd(9.0, 9.0)
5	3.89^a^	3.89^a^	3.94^a^
6a	4.71^a^	4.64 br d(11.5)	4.73 br d(11.0)
6b	4.34 dd(4.0, 11.0)	4.26 dd(5.0, 11.5)	4.38 dd(6.0, 11.0)
Glc''''-1	5.01 d(8.0)	4.96^a^	5.06 d(8.0)
2	4.03 dd(8.0, 9.0)	3.97 dd(8.0, 9.0)	4.03 dd(8.0, 9.0)
3	4.12 dd(9.0, 9.0)	4.17 dd(9.0, 9.0)	4.20 dd(9.0, 9.0)
4	4.24^a^	4.23^a^	4.25^a^
5	3.92^a^	3.88^a^	3.94^a^
6a	4.44 dd(2.0, 11.5)	4.51^a^	4.55^a^
6b	4.38^a^	4.37^a^	4.40 dd(5.0, 12.0)
Agl-2a	2.60 ddd(7.5, 7.5, 16.0)	2.60 ddd(7.5, 7.5, 16.5)	2.62 dd(8.0, 8.0, 16.0)
2b	2.45^a^	2.44 ddd(7.0, 7.0, 16.5)	2.42^a^
10a	1.76^a^	1.70^a^	
10b	1.65^a^	1.63^a^	
11	3.77^a^	3.78^a^	
12a	1.84^a^	1.86^a^	3.76^a^
12b	1.62^a^	1.59^a^	
13a	1.50^a^	1.48^a^	1.83^a^
13b			1.57^a^
14	1.25^a^	1.28^a^	1.45^a^
15	1.29^a^	1.30^a^	1.16^a^
16	0.87 t(7.5)	0.87 t(7.5)	0.89 t(7.5)
Nla-2	2.84 dq(7.0, 7.0)	2.84 dq(7.0, 7.0)	2.82 dq(7.0, 7.0)
3	4.36^a^	4.37^a^	4.36^a^
4	1.37 d(7.0)	1.37 d(7.0)	1.36 d(7.0)
5	1.36 d(7.0)	1.36 d(7.0)	1.32 d(7.0)
Nla'-2	3.11 dq(7.0, 7.0)	3.10 dq(7.0, 7.0)	3.17 dq(7.0, 7.0)
3	4.44 m	4.44 dq(7.0, 7.0)	4.43 m
4	1.44 d(7.0)	1.44 d(7.0)	1.45 d(7.0)
5	1.48 d(7.0)	1.48 d(7.0)	1.50 d(7.0)
Mba-2	2.34 ddq(7.0, 7.0, 7.0)	2.33 ddq(7.0, 7.0, 7.0)	2.39 ddq(7.0, 7.0, 7.0)
3a	1.70 m	1.69 m	1.75^a^
3b	1.44^a^	1.40 m	1.46^a^
4	0.78 dd(7.5, 7.5)	0.77 dd(7.5, 7.5)	0.80 dd(7.5, 7.5)
5	1.16 d(7.0)	1.15 d(7.0)	1.23 d(7.0)
Mba'-2	2.41 ddq(7.0, 7.0, 7.0)		2.44 ddq(7.0, 7.0, 7.0)
3a	1.67^a^		1.75^a^
3b	1.37^a^		1.39^a^
4	0.87 dd(7.5, 7.5)		0.91 dd(7.5, 7.5)
5	1.10 d(7.0)		1.15 d(7.0)
Mba"-2			2.53 ddq(7.0, 7.0, 7.0)
3a			1.83^a^
3b			1.53^a^
4			0.95 dd(7.5, 7.5)
5			1.23 d(7.0)
Tig-3	7.05 qq(1.0, 7.0)	7.01 qq(1.5, 7.0)	
4	1.63 d(7.0)	1.56 d(7.0)	
5	1.91 br s	1.86 br s	
Tig-'-3		6.93 qq(1.5, 7.0)	
4		1.56 d(7.0)	
5		1.78 br s	

$\delta$ in ppm from TMS [coupling constants (*J*) in Hz are given in parentheses]

*Qui* quinovopyranosyl, *Fuc* fucopyranosyl, *Glc* glucopyranosyl, *Rha* rhamnopyranosyl, *Agl* Aglycone moiety, *Nla* niloyl, *Mba* 2-methylbutyryl, *Tig* tigloyl

^a^Signals were overlapped with other signals

^b^*J* = 6.0 Hz arose from coupling of hydroxyl proton ($\delta$ 7.47)

Compound **5** (calyhedin XXVIII) was obtained as an amorphous powder. Both negative-ion and positive-ion FAB-MS indicated that the exact mass of **5** was 1804, which is two mass units less than that of **4**, and its molecular formula was determined to be C_83_H_136_O_42_ using HR positive-ion FAB-MS. The ^1^H- and ^13^C-NMR spectra of **5** were similar to those of **4**, except for the appearance of additional signals due to the 1 mol of tigloyl residue, and the absence of signals due to the 1 mol 2-methylbutyryl residue (Tables 2 and 3). In the ^1^H-NMR spectrum of **5**, the acylation shifts were observed for the same signals as those of **4**. In addition, fragment ion peaks were observed at *m/z* 1171 in negative-ion FAB-MS and at *m/z* 493 in positive-ion FAB-MS, indicating that 1 mol each of niloyl and 2-methylbutyryl residues and 2 mol of tigloyl residues were located in Rha, Rha, Glc', and Glc', respectively (Fig. 3). In the HMBC spectrum of **5**, significant correlations were observed between H-6a and H-6b of Glc and C-1 of Nla, H-2 of Rha and C-1 of Mba, H-4 of Rha and C-1 of Nla', H-3 of Glc' and C-1 of the first tigloyl residue (Tig) or C-1 of the second tigloyl residue (Tig'), H-4 of Glc' and C-1 of Tig' or C-1 of Tig, and H-2 of Glc''' and C-1 of Agl (Fig. 2). Although the ^13^C-NMR signals attributed to C-1 of Tig and C-1 of Tig' appeared at almost the same chemical shifts, the ^1^H- and ^13^C-NMR signals were assigned referring to those of **3** and **4**. Accordingly, **5** was proposed as an analog of **4**; the 2*S*-methylbutyryl residue at C-4 of Glc' in **4** was replaced with a tigloyl residue (Fig. 1).

Compound **6** (calyhedin XXIX) was obtained as an amorphous powder. Using HR-positive-ion FAB-MS, the molecular formula of **6** was determined to be C_83_H_140_O_42_, which is the same as that of calyhedin VII (**19**) [14]. The signals attributable to the sugar moiety in the ^1^H- and ^13^C-NMR spectra of **6** were also quite similar to those of **19**, including acylation shift signals in the ^1^H-NMR spectrum. In contrast, the ^13^C-NMR signals attributed to Agl were similar to those of **9**–**12** and **14** (Tables 1 and 2). These data suggested that **6** is an isomer of **19**, in which a 12*S*-hydroxyhexadecanoyl residue replaces the 11*S*-jalapinoyl residue of **19**. This assumption was confirmed by the HMBC spectrum and FAB-MS of **6**. In the HMBC spectrum of **6**, important cross-peaks were observed between Ha-6 and Hb-6 of Glc and C-1 of Nla or C-1 of Mba' or C-1 of Mba", H-2 of Rha and C-1 of Mba, H-3 of Rha and C-1 of Nla', H-3 of Glc' and C-1 of Mba' or C-1 of Nla or C-1 of Mba", H-4 of Glc' and C-1 of Mba" or C-1 of Nla or C-1 of Mba', and H-2 of Glc''' and C-1 of Agl (Fig. 2). However, the counterparts of Ha-6 and Hb-6 of Glc, H-3 of Glc', and H-4 of Glc' could not be identified because the ^13^C-NMR signals of C-1 of Nla, C-1 of Mba', and C-1 of Mba" appeared at almost the same chemical shifts. Finally, all ester bonds were confirmed by FAB-MS of **6**. The fragment ion peaks observed at *m/z* 1315 and 1171 in the negative-ion FAB-MS of **6** indicated that the ester linkages of Nla, Mba, and Nla' were located in Glc, Rha, and Rha, respectively, whereas the positive-ion FAB-MS of **6** revealed a fragment ion peak at *m/z* 493, indicating that both locations of Mba and Nla' were Rha (Fig. 3). Consequently, the structure of **6** was determined to be 12*S*-hydroxyhexadecanoic acid 12-*O*-β-d-glucopyranosyl-(1 → 6)-*O*-β-d-glucopyranosyl-(1 → 6)-*O*-(3,4-di-*O*-2*S*-methylbutyryl)-β-d-glucopyranosyl-(1 → 3)-[*O*-β-d-glucopyranosyl-(1 → 3)-*O*-(2-*O*-2*S*-methylbutyryl-4-*O*-2*R*,3*R*-niloyl)-α-l-rhamnopyranosyl-(1 → 2)]-*O*-(6-*O*-2*R*,3*R*-niloyl)-β-d-glucopyranosyl-(1 → 2)-β-d-quinovopyranoside, intramolecular 1,2''''''-ester (Fig. 1). The assignments of the NMR signals for Mba' and Mba" were based on data from **19** [14].

Compound **7** (calyhedin XXX) was obtained as an amorphous powder. The molecular formula of **7** was determined to be the same as that of **5** by using HR positive-ion FAB-MS. The signals in the ^1^H- and ^13^C-NMR spectra of **7** were similar to those of **13**, except for the appearance of additional signals due to the 2 mol tigloyl residues and the disappearance of signals corresponding to the 2 mol 2-methylbutyryl residues (Tables 2 and 4). In particular, the Agl signals were nearly identical. These data suggested that **7** is a positional isomer of **5** with respect to the ester linkage of Agl. The HMBC spectrum of **7** showed important cross-peaks between H-6b of Glc and C-1 of Nla or C-1 of Nla', H-2 of Rha and C-1 of Mba, H-4 of Rha and C-1 of Nla' or C-1 of Nla, H-2 of Glc' and C-1 of Agl, H-3 of Glc' and C-1 of Tig, and H-4 of Glc' and C-1 of Tig' (Fig. 2). In addition, fragment ion peaks were observed at *m/z* 1171 in negative-ion FAB-MS and at *m/z* 493 in positive-ion FAB-MS (Fig. 3). Accordingly, we concluded that **7** is a positional isomer of **5**, in which the ester linkage of Agl of **7** is at C-2 of Glc' rather than at C-2 of Glc''' (Fig. 1).

**Table 4:** ^1^H-NMR spectral data for **7** and **8** (in pyridine-*d*_5_, 600 MHz)

Position	**7**	**8**
Qui-1	4.75 d(7.5)	4.89 d(8.0)
2	4.08^a^	4.21^a^
3	4.44 dd(9.5, 9.5)	4.49 dd(9.0, 9.0)
4	3.62 dd(9.5, 9.5)	3.65 dd(9.0, 9.0)
5	3.91^a^	3.98^a^
6	1.61 d(6.0)	1.63 d(6.0)
Glc-1	5.61 d(7.5)	5.73 d(7.5)
2	4.13 dd(7.5, 9.0)	4.10 dd(7.5, 9.5)
3	4.67 dd(9.0, 9.0)	3.86^a^
4	3.85^a^	3.81^a^
5	4.08^a^	3.81^a^
6a	4.73 br d(12.0)	4.71 br d(11.0)
6b	4.66 dd(4.0, 12.0)	4.67 dd(4.5, 11.0)
Rha-1	5.81 s	6.25 s
2	6.11 d(3.5)	6.11 br s
3	5.17 dd(3.5, 10.0)	5.19 br d(10.0)
4	5.90 dd(10.0, 10.0)	5.89 dd(10.0, 10.0)
5	5.30 dq(10.0, 6.5)	5.23^a^
6	1.77 d(6.5)	1.75 d(6.0)
Glc'-1	5.38 d(8.0)	4.89 d(8.0)
2	5.46 dd(8.0, 9.0)	3.82^a^
3	6.00 dd(9.0, 9.0)	5.67 dd(9.5, 9.5)
4	5.35 dd(9.0, 9.0)	5.30 dd(9.5, 9.5)
5	4.42^a^	4.23^a^
6a	4.39^a^	4.41 br d(11.0)
6b	4.06^a^	3.87^a^
Glc"-1	5.12 d(8.0)	5.24 d(7.0)
2	3.90^a^	3.92^a^
3	4.04^a^	4.14^a^
4	3.91^a^	3.94^a^
5	3.84^a^	4.21^a^
6a	4.38^a^	4.55 br d(11.5)
6b	4.08^a^	4.15^a^
Glc'''-1	5.13 d(7.5)	4.78 d(7.5)
2	3.90^a^	3.93^a^
3	4.21 dd(9.0, 9.0)	4.13^a^
4	4.09^a^	4.22^a^
5	4.08^a^	3.98^a^
6a	4.76 dd(1.0, 11.0)	4.71 br d(11.0)
6b	4.28 dd(5.5, 11.0)	4.31 dd(5.0, 11.0)
Glc''''-1	5.02 d(7.5)	5.01^a^
2	4.03 dd(7.5, 9.0)	4.01 dd(7.5, 9.0)
3	4.19 dd(9.0, 9.0)	4.22^a^
4	4.24 dd(9.0, 9.0)	4.15^a^
5	3.90^a^	3.92^a^
6a	4.50 dd(3.5, 12.0)	4.52 br d(12.0)
6b	4.37^a^	4.37 dd(6.0, 12.0)
Agl-2a	2.60^a^	2.36 t(7.5)
2b	2.54^a^	
10a		1.75^a^
10b		1.68^a^
11	3.65^a^	3.85^a^
12a	1.85^a^	1.77^a^
12b	1.62^a^	1.75^a^
13	1.45^a^	1.40^a^
14	1.27^a^	1.28^a^
15	1.30^a^	1.33^a^
16	0.87 t(7.0)	0.88 t(7.0)
Nla-2	2.84 dq(7.0, 7.0)	2.83 dq(7.0, 7.0)
3	4.39^a^	4.38^a^
4	1.35 d(7.0)	1.37 d(7.0)
5	1.26 d(7.0)	1.30 d(7.0)
Nla'-2	3.12 dq(7.5, 7.5)	3.11 dq(7.0, 7.0)
3	4.41^a^	4.45 dq(7.0, 7.0)
4	1.41 d(6.0)	1.44 d(7.0)
5	1.45 d(7.5)	1.49 d(7.0)
Mba-2	2.58 ddq(7.0, 7.0, 7.0)	2.40 ddq(7.0, 7.0, 7.0)
3a	1.84^a^	1.75^a^
3b	1.52^a^	1.45^a^
4	0.82 dd(7.5, 7.5)	0.81 dd(7.5, 7.5)
5	1.27 d(7.0)	1.23 d(7.0)
Mba'-2		2.46 ddq(7.0, 7.0, 7.0)
3a		1.80^a^
3b		1.52^a^
4		0.94 dd(7.5, 7.5)
5		1.19 d(7.0)
Mba"-2		2.37^a^
3a		1.70^a^
3b		1.40^a^
4		0.90 dd(7.5, 7.5)
5		1.12 d(7.0)
Tig-3	6.92^a^	
4	1.45 br d(7.0)	
5	1.74 br s	
Tig'-3	6.92^a^	
4	1.49 brd(7.0)	
5	1.98 br s	

$\delta$in ppm from tetramethylsilane (TMS) [coupling constants (*J*) in Hz are given in parentheses]

*Qui* quinovopyranosyl, *Fuc* fucopyranosyl, *Glc* glucopyranosyl, *Rha* rhamnopyranosyl, *Agl* Aglycone moiety, *Nla* niloyl, *Mba* 2-methylbutyryl, *Tig* tigloyl

^a^Signals were overlapped with other signals

Compound **8** (calyhedin XXXI) was obtained as an amorphous powder. It exhibited an [M + Na]^+^ ion peak at *m/z* 1863 in positive-ion FAB-MS and an [M–H]^–^ ion peak at *m/z* 1839 in negative-ion FAB-MS, indicating an exact mass of 1840. HR positive-ion FAB-MS revealed that the molecular formula of **8** was C_84_H_144_O_43_. The ^1^H-NMR spectrum of **8** indicated signals corresponding to three H-2 of 2-methylbutyryl residues, two H-2 of niloyl residues, one methoxy group, nine secondary methyl groups, four primary methyl groups, two equivalent methylene protons adjacent to a carbonyl group, and some monosaccharide residues (Table 4). These data suggested that **8** is a non-macrolactone-type resin glycoside composed of 3 mol of 2*S*-methylbutyric acid and 2 mol of 2*R*,3*R*-nilic acid. The ^13^C-NMR spectrum of **8** showed signals attributed to seven anomeric carbons and six ester carbonyl carbons (Table 2). ^1^H- and ^13^C-NMR signals were assigned using 2D-NMR techniques, similar to those used to assign the signals for **1**–**7**. The ^13^C-NMR data for Agl were almost identical to those for the calyjaponic acid B methyl ester [11], indicating that the aglycone of **8** was methyl jalapinolate. In addition, the HMBC spectrum of **8** revealed key cross-peaks between H-1 of Qui or H-1 of Glc' and C-11 of Agl or C-3 of Glc, H-2 of Glc and C-1 of Rha, H-1 of Glc" and C-3 of Rha, H-3 of Rha and C-1 of Glc", H-1 of Glc''' and C-6 of Glc', and H-1 of Glc'''' and C-6 of Glc''' (Fig. 2). Although the ^1^H-NMR signals attributable to H-1 of Qui and H-1 of Glc' appeared at nearly identical chemical shifts, and no HMBC correlations were observed for the glycosidic linkage at C-1 of Glc, glycosylation shifts [17, 18] were detected at C-11 of Agl, C-2 of Qui, and C-3 of Glc. This supported the conclusion that the glycosidic acid component of **8** is calyhedic acid C (**20**) [12]. A comparison of the ^1^H-NMR signals due to the sugar moiety between **8** and **20** showed acylation shifts (Δ$\delta$ = $\delta$**8**–$\delta$**20**) of ^1^H-NMR signals due to Ha-6 (Δ$\delta$ = 0.30) of Glc, Hb-6 (Δ$\delta$ = 0.53), H-2 (Δ$\delta$ = 0.97) and H-4 (Δ$\delta$ = 1.37) of Rha, and H-3 (Δ$\delta$ = 1.48) and H-4 (Δ$\delta$ = 1.40) of Glc'. In addition, significant HMBC correlations were observed between the methoxy protons and C-1 of Agl, H-2 of Rha and C-1 of Mba, H-4 of Rha and C-1 of Nla' or C-1 of Nla, H-3 of Glc' and C-1 of Mba", and H-4 of Glc' and C-1 of Mba' (Fig. 2). Furthermore, positive-ion FAB-MS of **8** revealed a fragment ion peak at *m/z* 493. Consequently, the structure of **8** was defined as methyl 11*S*-jalapinolate 11-*O*-β-d-glucopyranosyl-(1 → 6)-*O*-β-d-glucopyranosyl-(1 → 6)-*O*-(3,4-di-*O*-2*S*-methylbutyryl)-β-d-glucopyranosyl-(1 → 3)-[*O*-β-D-glucopyranosyl-(1 → 3)-*O*-(2-*O*-2*S*-methylbutyryl,4-*O*-2*R*,3*R*-niloyl)-α-l-rhamnopyranosyl-(1 → 2)]-*O*-(6-*O*-2*R*,3*R*-niloyl)-β-d-glucopyranosyl-(1 → 2)-β-d-quinovopyranoside (Fig. 1).

We previously reported the cytotoxicity of calyhedins II (**9**), III (**10**), VII, VIII (**13**), X, XI, XV, XVII, XVIII, XIX, XX, XXI, XXII, and XXIII, obtained from *C. hederacea* against HL-60 cells [5–8]. Therefore, we evaluated the cytotoxicity of **1**–**8**, **11**, **12**, and **14** in HL-60 cells. Except for **1**, the other ten tested compounds showed apparent cytotoxicity against HL-60 cells, and the IC_50_ values for **3**, **4**, **6**, **7**, **11**, and **12** were closer to or lower than that of the positive control cisplatin. These cytotoxicity findings showed no clear structure–activity relationship in relation to differences in the type of organic acid bound to the sugar moiety, size of the macrolactone ring, and sugar linkage.

To date, several jalapins with anti-HSV-1 activities have been reported [4, 5, 20, 21, 22]. Therefore, we performed an activity assay on **1**–**14**, along with 11 previously isolated jalapins from *C. hederacea* (calyhedins VII (**19**), X (**21**), XI (**22**), XV (**23**), XVII (**24**), XVIII (**25**), XIX (**26**), XX (**27**), XXI (**16**), XXII (**28**), and XXIII (**29**)). All the tested compounds exhibited anti-HSV-1 activity, and seven (**3**, **6**, **9**, **10**, **22**, **23**, and **24**) had EC_50_ values lower than or comparable to that of the positive control, acyclovir. Compounds **23** and **24** exhibited relatively high selectivity indices. Although no clear structure–activity relationship was observed, compounds with a jalapin structure showed stronger activity than the acylated glycosidic acid methyl ester (see **13** and **19** *vs*. **8**). Compounds with 27- and 28-membered rings tended to show stronger activity than the corresponding compounds with 22- and 23-membered rings (see **5** *vs*. **7**, **25** *vs*.**27**, **9** *vs*. **2**), although **13** with 22-membered ring and **19** with 27-membered ring showed almost identical activity. The glucosyl group (Glc") attached to the C-3 of Rha tended to enhance the activity (see **1** *vs*. **27**).

Further research is required to elucidate the structure–activity relationships of resin glycosides in relation to their cytotoxic effects on HL-60 cells and their anti-HSV-1 activities.

## Conclusion

Our investigation of air-dried rhizomes of *C. hederacea* resulted in the isolation and structural elucidation of eight new (**1**–**8**) and six known resin glycosides (**9**–**14**). Compounds **1**–**7** had lactone structures similar to those of the resin glycosides previously isolated from *C. hederacea*. Therefore, they can be classified as jalapin-type resin glycosides [4] and include macrolactones with 22- (**1** and **7**), 23- (**2**), 27- (**3**–**5**), or 28- (**6**) membered rings. Compound **8** was an acylated glycosidic acid methyl ester. This compound may have been an artifact generated by macrolactone resin glycosides such as **13** and **19** during extraction, isolation, or both. In addition, the cytotoxic activity of the 11 compounds isolated in this study against HL-60 human promyelocytic leukemia cells and the antiviral activity of the 14 compounds isolated in this study and 11 previously isolated calyhedins against HSV-1 were evaluated. With the exception of **1**, all tested compounds demonstrated dual activity.

## Experimental section

### General procedure

The optical rotations were measured with a JASCO P-2300 polarimeter (JASCO, Tokyo, Japan). The ^1^H- and ^13^C-NMR spectra were recorded by using an ECZ-600R/S1 spectrometer (JEOL, Tokyo, Japan), and chemical shifts are reported on a $\delta$ (ppm) scale with tetramethylsilane (TMS) as the internal standard. MS data were collected using a JEOL JMS-700 mass spectrometer (JEOL). Column chromatography was carried out over Chromatorex ODS (Fuji Silysia Chemical, Ltd., Aichi, Japan). HPLC separation was performed on a Shimadzu LC-10AS micropump using a Shimadzu RID-10A RI detector (Shimadzu). For HPLC column chromatography, Inertsil ODS-HL (GL Sciences, Tokyo, Japan; 20 mm i.d. × 250 mm, column 1), COSMOSIL 5C_18_-AR-II (Nacalai Tesque, Inc., Kyoto, Japan; 20 mm i.d. × 250 mm, column 2), and COSMOSIL π-nap (Nacalai Tesque, Inc; 20 mm i.d. × 250 mm, column 3) were used.

### Plant materials

The rhizomes of *C. hederacea* were collected in September 2016 in Fukuoka Prefecture, Japan, and identified by one of the authors (Prof. Okawa M.). A voucher specimen (CHRFU2016) has been deposited at the Laboratory of Natural Products Chemistry, School of Agriculture, Tokai University.

### Cells

The HL-60 human promyelocytic leukemia cells (JCRB0085) were obtained from Japanese Collection of Research Bioresources (Tokyo, Japan). HSV-1 strain (KOS) and Vero cells were provided by the Chemo-Sero Therapeutic Institute (Kumamoto, Japan).

### Isolation of **1**–**14**

Fractions 1-6, 1-7, and 1-9 were previously separated from the MeOH extract of the air-dried rhizomes of *C. hederacea* [8] Fraction 1-6 (1314 mg) was applied to Chromatorex ODS column (solvent, 80% MeOH, 90% MeOH, 100% MeOH) to yield frs. 1-6-1–1-6-6. Fraction 1-6-3 (556 mg) was subjected to HPLC (column, column 1; solvent 90% MeOH) to afford **3** (19 mg), **2** (23 mg), and **1** (10 mg). HPLC (column, column 2; solvent, 95% MeOH) of fr. 1-7 (1.032 g) afforded **10** (18 mg) and **9** (14 mg). Chromatography of fr. 1-9 (2.661 g) over Chromatorex ODS column (solvent, 85% MeOH, 90% MeOH, 95% MeOH, 100% MeOH) to yield frs. 1-9-1–1-9-5. Fraction 1-9-3 (1.081 g) was subjected to HPLC (column, column 1; solvent 90% MeOH) to afford frs. 1-9-3-1–1-9-3-7. Fractions 1-9-3-2 (122 mg), 1-9-3-3 (152 mg), 1-9-3-4 (133 mg), and 1-9-3-5 (122 mg) were each subjected to HPLC (column, column 3; solvent, 95% MeOH) to yield **8** (18 mg), **12** (22 mg), and **5** (19 mg) from fr. 1-9-3-2, **4** (20 mg) from fr. 1-9-3-3, **11** (18 mg) from 1-9-3-4, and **14** (9 mg), and **7** (9 mg) from fr. 1-9-3-5. Fraction 1-9-4 (614 mg) was applied to HPLC (column, column 1; solvent, 90% MeOH)) to yield frs. 1-9-4-1–1-9-4-4 and **13** (57 mg). Fraction 1-9-4-2 (69 mg) was subjected to HPLC (column, column 3; solvent 95% MeOH) to afford **6** (9 mg).

Calyhedin XXIV (**1**): amorphous powder. [α]^25^_D_ –13.7°(*c* = 1.3, MeOH). Positive-ion FAB-MS *m/z*: 1669 [M + Na]^+^, 493. HR positive-ion FAB-MS *m/z*: 1669.8188 (Calcd for C_77_H_130_O_37_Na^+^, 1669.8183). Negative-ion FAB-MS *m/z*: 1645 [M–H]^–^, 1545 [1645–100]^–^, 1383 [1545–162 (hexosyl unit)]^–^, 1153 [1383–146–84]^–^, 417 [1153–162 × 2–144–100–84 × 2]^–^. ^1^H-NMR spectral data: see Table 1. ^13^C-NMR spectral data: see Table 2.

Calyhedin XXV (**2**): amorphous powder. [α]^26^_D_ –14.6° (*c* = 2.4, MeOH). Positive-ion FAB-MS *m/z*: 1669 [M + Na]^+^, 493. HR positive-ion FAB-MS *m/z*: 1669.8187 (Calcd for C_77_H_130_O_37_Na^+^, 1669.8183). Negative-ion FAB-MS *m/z*: 1645 [M–H]^–^, 1545 [1645–100]^–^, 1153 [1383–146–84]^–^, 417 [1153–162 × 2–144–100–84 × 2]^–^. ^1^H-NMR spectral data: see Table 1. ^13^C-NMR spectral data: see Table 2.

Calyhedin XXVI (**3**): amorphous powder. [α]^26^_D_ –11.3° (*c* = 2.9, MeOH). Positive-ion FAB-MS *m/z*: 1667 [M + Na]^+^, 493. HR positive-ion FAB-MS *m/z*: 1667.8042 (Calcd for C_77_H_128_O_37_Na^+^, 1667.8027). Negative-ion FAB-MS *m/z*: 1643 [M–H]^–^, 1561 [1643–82]^–^, 1499 [1643–144]^–^, 1171 [1499–162–84–82]^–^, 1151 [1643–162–146–100–84]^–^, 417 [1151–162 × 2–144–100–84–82]^–^. ^1^H-NMR spectral data: see Table 1. ^13^C-NMR spectral data: see Table 2.

Calyhedin XXVII (**4**): amorphous powder. [*α*]^24^_D_ –21.9° (*c* = 2.6, MeOH). Positive-ion FAB-MS *m/z*: 1829 [M + Na]^+^, 493. HR positive-ion FAB-MS *m/z*: 1829.8574 (Calcd for C_83_H_138_O_42_Na^+^, 1829.8555). Negative-ion FAB-MS *m/z*: 1805 [M–H]^–^, 1499 [1805–144–162]^–^, 1313 [1807–84–100–146–162]^–^, 417 [1171–84–100 × 2–146–162 × 2]^–^, 271 [417–146]^–^. ^1^H-NMR spectral data: see Table 3. ^13^C-NMR spectral data: see Table 2.

Calyhedin XXVIII (**5**): amorphous powder. [*α*]^24^_D_ –21.5° (*c* = 2.3, MeOH). Positive-ion FAB-MS *m/z*: 1827 [M + Na]^+^, 493. HR positive-ion FAB-MS *m/z*: 1827.8384 (Calcd for C_83_H_136_O_42_Na^+^, 1827.8398). Negative-ion FAB-MS *m/z*: 1803 [M–H]^–^, 1171 [1501–84 × 2–162]^–^, 417 [1171–84–100 × 2–146–162 × 2]^–^, 271 [417–146]^–^. ^1^H-NMR spectral data: see Table 3. ^13^C-NMR spectral data: see Table 2.

Calyhedin XXIX (**6**): amorphous powder. [α]^27^_D_ –17.5° (*c* = 1.1, MeOH). Positive-ion FAB-MS *m/z*: 1831 [M + Na]^+^, 493. HR positive-ion FAB-MS *m/z*: 1831.8726 (Calcd for C_83_H_140_O_42_Na^+^, 1831.8711). Negative-ion FAB-MS *m/z*: 1807 [M–H]^–^, 1501 [1807–144–162]^–^, 1315 [1807–84–100–146–162]^–^, 1171 [1501–84 × 2–162]^–^, 417 [1171–84–100 × 2–146–162 × 2]^–^. ^1^H-NMR spectral data: see Table 3. ^13^C-NMR spectral data: see Table 2.

Calyhedin XXX(**7**): amorphous powder. [*α*]^27^_D_ –32.4° (*c* = 1.1, MeOH). Positive-ion FAB-MS *m/z*: 1827 [M + Na]^+^, 493. HR positive-ion FAB-MS *m/z*: 1827.8387 (Calcd for C_83_H_136_O_42_Na^+^, 1827.8398). Negative-ion FAB-MS *m/z*: 1803 [M–H]^–^, 1171 [1501–84 × 2–162]^–^, 417 [1171–84–100 × 2–146–162 × 2]^–^. ^1^H-NMR spectral data: see Table 4. ^13^C-NMR spectral data: see Table 2.

Calyhedin XXXI (**8**): amorphous powder. [*α*]^23^_D_ –12.1° (*c* = 2.3, MeOH). Positive-ion FAB-MS *m/z*: 1863 [M + Na]^+^, 493. HR positive-ion FAB-MS *m/z*: 1863.8965 (calcd for C_84_H_144_O_43_Na^+^, 1863.8974). Negative-ion FAB-MS *m/z*: 1839 [M–H]^–^, 1171. ^1^H-NMR spectral data, see Table 4. ^13^C-NMR, see Table 2.

Cytotoxic assay for HL-60 cells.

**HL-60 cells seeded in individual wells** of a 96-well culture plate at a density of 5 × 10^4^ cells/100 µL per well were incubated in the presence of test samples. A cell counting kit-8 (CCK-8) containing 2-(2-methoxy-4-nitrophenyl)-3-(4-nitrophenyl)-5-(2,4-disulphophenyl)-2*H*-tetrazolium monosodium salt (WST-8) was used to measure the activities of dehydrogenase enzyme(s) in viable cells according to the manufacturer’s instruction (Dojindo Labs, Kumamoto, Japan). After incubation for 24 h, CCK-8 solution was added to each well, followed by another 3 h of incubation. To determine cytotoxic activity, the reduction of WST-8 was determined colorimetrically at 450 nm using a grating microplate reader (SH-1000Lab, Corona Electric, Ibaraki, Japan). Cisplatin was used as a positive control. Data shown represent mean ± S.D. derived from three experiments (Tables 5 and 6).

**Table 5:** Cytotoxic activity (IC_50_) of **1**–**8**, **11**, **12**, **14**, and cisplatin

Sample	IC_50_ (µM)
**1**	>50
**2**	12.9 ± 2.8
**3**	8.19 ± 0.04
**4**	6.01 ± 0.28
**5**	13.3 ± 0.3
**6**	9.73 ± 0.70
**7**	6.96 ± 2.10
**8**	14.4 ± 0.1
**11**	9.80 ± 2.62
**12**	8.05 ± 0.21
**14**	13.6 ± 1.0
Cisplatin	10.3 ± 1.6

Data shown represent mean ± S.D. derived from three experiments. Cisplatin was used as a positive control

**Table 6:** Anti-herpes activity (EC_50_), cytotoxic activity (IC_50_), and selectivity Index (IC_50_/EC_50_) of **1**–**14**, **16**, **19**, **21**–**29**, and acyclovir

Sample	EC_50_ (µM)	IC_50_ (µM)	IC_50_/EC_50_
**1**	12.5 ± 4.0	> 200	> 16
**2**	7.0 ± 0.5	6.3 ± 1.6	0.9
**3**	2.6 ± 0.8	5.3 ± 4.5	2.0
**4**	8.9 ± 0.0	6.7 ± 1.2	0.8
**5**	4.3 ± 0.2	17.5 ± 5.7	4.1
**6**	2.2 ± 0.1	6.4 ± 1.7	2.9
**7**	17.4 ± 0.5	10.3 ± 3.6	0.6
**8**	27.0 ± 3.5	10.1 ± 0.3	0.4
**9**	1.8 ± 0.0	51.6 ± 5.4	28.7
**10**	1.8 ± 0.0	23.7 ± 1.4	13.2
**11**	16.8 ± 0.9	38.8 ± 0.0	2.3
**12**	8.9 ± 0.0	64.6 ± 1.6	7.3
**13**	8.7 ± 0.2	31.3 ± 2.8	3.6
**14**	16.5 ± 0.6	39.4 ± 1.3	2.4
**16**	33.6 ± 0.2	> 200	> 6.0
**19**	8.9 ± 0.0	102.9 ± 17.1	11.6
**21**	26.0 ± 8.3	52.1 ± 7.6	2.0
**22**	3.4 ± 0.3	64.0 ± 0.9	18.8
**23**	2.3 ± 0.2	> 200	> 87.0
**24**	3.5 ± 0.4	> 200	> 57.1
**25**	12.8 ± 0.4	151.3 ± 2.6	11.8
**26**	6.5 ± 0.5	155.0 ± 2.6	23.8
**27**	70.7 ± 7.0	> 200	> 2.8
**28**	15.9 ± 1.6	> 200	> 12.6
**29**	14.1 ± 0.2	> 200	> 14.2
Acyclovir	3.5 ± 0.2	> 200	> 57.1

EC_50_ and IC_50_ data represent the mean ± S.D. derived from two and four experiments, respectively. Acyclovir was used as a positive control

### Anti-HSV-1 assay

The antiviral activity of test samples against HSV-1 was using a plaque reduction assay [23]. Confluent monolayers of Vero cells in 6-well plates were infected with HSV-1 at 100 plaque-forming units per well. Following a 1 h adsorption period, the cultures were overlaid with Dulbecco’s modified Eagle minimum essential medium (DMEM) containing 2% heat-inactivated fetal calf serum (FCS) and 2% sulfonated γ-globulin including various concentrations of the test samples. The plates were incubated in the CO_2_ incubator for 3 days, then fixed with formalin and stained with crystal violet in methanol. Infections HSV-1 production was quantified by observing the virus-induced cytopathic effect. The cytotoxic activity against Vero cells was measured by the 3-(4,5-dimethyl-thiazol-2-yl)-2,5-diphenyl tetrazolium bromide (MTT) assay. The vero cells were seeded in 96-well plates at 1 × 10^4^ cells per well. After 1 day incubation, the cells were refed with DMEM containing 5% FBS and various concentrations of the test samples. After 3 days incubation, cells were washed with PBS and incubated for 4 h with MTT solution at a final concentration of 0.5 mg/mL. Isopropanol and hydrochloric acid were added to the culture medium at final concentrations of 50% and 20 mM, respectively. The optical density of each well at 570 nm was determined spectrophotometrically using a reference wavelength of 630 nm. Acyclovir was used as a positive control in the anti-HSV assay.

## Supplementary Information

Below is the link to the electronic supplementary material.

Supplementary file1 (PDF 35527 KB)
