# Supplementary material for: Eight new resin glycosides, calyhedins XXIV–XXXI, from the rhizomes of Calystegia hederacea
Source: J Nat Med. 2025 Jun 1;79(4):845–62. doi: 10.1007/s11418-025-01919-1 (PMC12228659; doi:10.1007/s11418-025-01919-1)
Supplement: Supplementary file 1 — Supplementary file1 (PDF 35527 KB) [file 11418_2025_1919_MOESM1_ESM.pdf]

Supplementary information

Eight new resin glycosides, calyhedins XXIV–XXXI, from the rhizomes of *Calystegia hederacea*

Masateru Ono · Yosuke Matsuoka · Ryota Arakawa · Hiroyuki Shimadzu · Takumi Nagasawa · Hirotaka Nishikawa · Shin Yasuda · Hiroyuki Miyashita · Kazumi Yokomizo · Hitoshi Yoshimitsu · Ryota Tsuchihasi · Masafumi Okawa · Junei Kinjo

List of Content

Figure S1: ^{1}H-NMR (600 MHz, pyridine-d_5) spectrum of **1**
Figure S2: ^{13}C-NMR (150 MHz, pyridine-d_5) spectrum of **1**
Figure S3: DEPT (150 MHz, pyridine-d_5) spectrum of **1**
Figure S4: ^{1}H-^{1}H COSY (600 MHz, pyridine-d_5) spectrum of **1**
Figure S5: ^{1}H-^{1}H TOCSY (600 MHz, pyridine-d_5) spectrum of **1**
Figure S6: HMQC (600 MHz, pyridine-d_5) spectrum of **1**
Figure S7: HMBC (600 MHz, pyridine-d_5) spectrum of **1**
Figure S8: ^{1}H-NMR (600 MHz, pyridine-d_5) spectrum of **2**
Figure S9: ^{13}C-NMR (150 MHz, pyridine-d_5) spectrum of **2**
Figure S10: DEPT (150 MHz, pyridine-d_5) spectrum of **2**
Figure S11: ^{1}H-^{1}H COSY (600 MHz, pyridine-d_5) spectrum of **2**
Figure S12: ^{1}H-^{1}H TOCSY (600 MHz, pyridine-d_5) spectrum of **2**
Figure S13: HMQC (600 MHz, pyridine-d_5) spectrum of **2**
Figure S14: HMBC (600 MHz, pyridine-d_5) spectrum of **2**
Figure S15: ^{1}H-NMR (600 MHz, pyridine-d_5) spectrum of **3**
Figure S16: ^{13}C-NMR (150 MHz, pyridine-d_5) spectrum of **3**
Figure S17: DEPT (150 MHz, pyridine-d_5) spectrum of **3**

Figure S18: ^{1}H-^{1}H COSY (600 MHz, pyridine-d_5) spectrum of **3**
Figure S19: ^{1}H-^{1}H TOCSY (600 MHz, pyridine-d_5) spectrum of **3**
Figure S20: HMQC (600 MHz, pyridine-d_5) spectrum of **3**
Figure S21: HMBC (600 MHz, pyridine-d_5) spectrum of **3**
Figure S22: ^{1}H-NMR (600 MHz, pyridine-d_5) spectrum of **4**
Figure S23: ^{13}C-NMR (150 MHz, pyridine-d_5) spectrum of **4**
Figure S24: DEPT (150 MHz, pyridine-d_5) spectrum of **4**
Figure S25: ^{1}H-^{1}H COSY (600 MHz, pyridine-d_5) spectrum of **4**
Figure S26: ^{1}H-^{1}H TOCSY (600 MHz, pyridine-d_5) spectrum of **4**
Figure S27: HMQC (600 MHz, pyridine-d_5) spectrum of **4**
Figure S28: HMBC (600 MHz, pyridine-d_5) spectrum of **4**
Figure S29: ^{1}H-NMR (600 MHz, pyridine-d_5) spectrum of **5**
Figure S30: ^{13}C-NMR (150 MHz, pyridine-d_5) spectrum of **5**
Figure S31: DEPT (150 MHz, pyridine-d_5) spectrum of **5**
Figure S32: ^{1}H-^{1}H COSY (600 MHz, pyridine-d_5) spectrum of **5**
Figure S33: ^{1}H-^{1}H TOCSY (600 MHz, pyridine-d_5) spectrum of **5**
Figure S34: HMQC (600 MHz, pyridine-d_5) spectrum of **5**
Figure S35: HMBC (600 MHz, pyridine-d_5) spectrum of **5**
Figure S36: ^{1}H-NMR (600 MHz, pyridine-d_5) spectrum of **6**
Figure S37: ^{13}C-NMR (150 MHz, pyridine-d_5) spectrum of **6**
Figure S38: DEPT (150 MHz, pyridine-d_5) spectrum of **6**
Figure S39: ^{1}H-^{1}H COSY (600 MHz, pyridine-d_5) spectrum of **6**
Figure S40: ^{1}H-^{1}H TOCSY (600 MHz, pyridine-d_5) spectrum of **6**
Figure S41: HMQC (600 MHz, pyridine-d_5) spectrum of **6**
Figure S42: HMBC (600 MHz, pyridine-d_5) spectrum of **6**
Figure S43: ^{1}H-NMR (600 MHz, pyridine-d_5) spectrum of **7**
Figure S44: ^{13}C-NMR (150 MHz, pyridine-d_5) spectrum of **7**
Figure S45: DEPT (150 MHz, pyridine-d_5) spectrum of **7**
Figure S46: ^{1}H-^{1}H COSY (600 MHz, pyridine-d_5) spectrum of **7**
Figure S47: ^{1}H-^{1}H TOCSY (600 MHz, pyridine-d_5) spectrum of **7**
Figure S48: HMQC (600 MHz, pyridine-d_5) spectrum of **7**
Figure S49: HMBC (600 MHz, pyridine-d_5) spectrum of **7**
Figure S50: ^{1}H-NMR (600 MHz, pyridine-d_5) spectrum of **8**

Figure S51: ^{13}C-NMR (150 MHz, pyridine-d_5) spectrum of **8**

Figure S52: DEPT (150 MHz, pyridine-d_5) spectrum of **8**

Figure S53: ^{1}H-^{1}H COSY (600 MHz, pyridine-d_5) spectrum of **8**

Figure S54: ^{1}H-^{1}H TOCSY (600 MHz, pyridine-d_5) spectrum of **8**

Figure S55: HMQC (600 MHz, pyridine-d_5) spectrum of **8**

Figure S56: HMBC (600 MHz, pyridine-d_5) spectrum of **8**

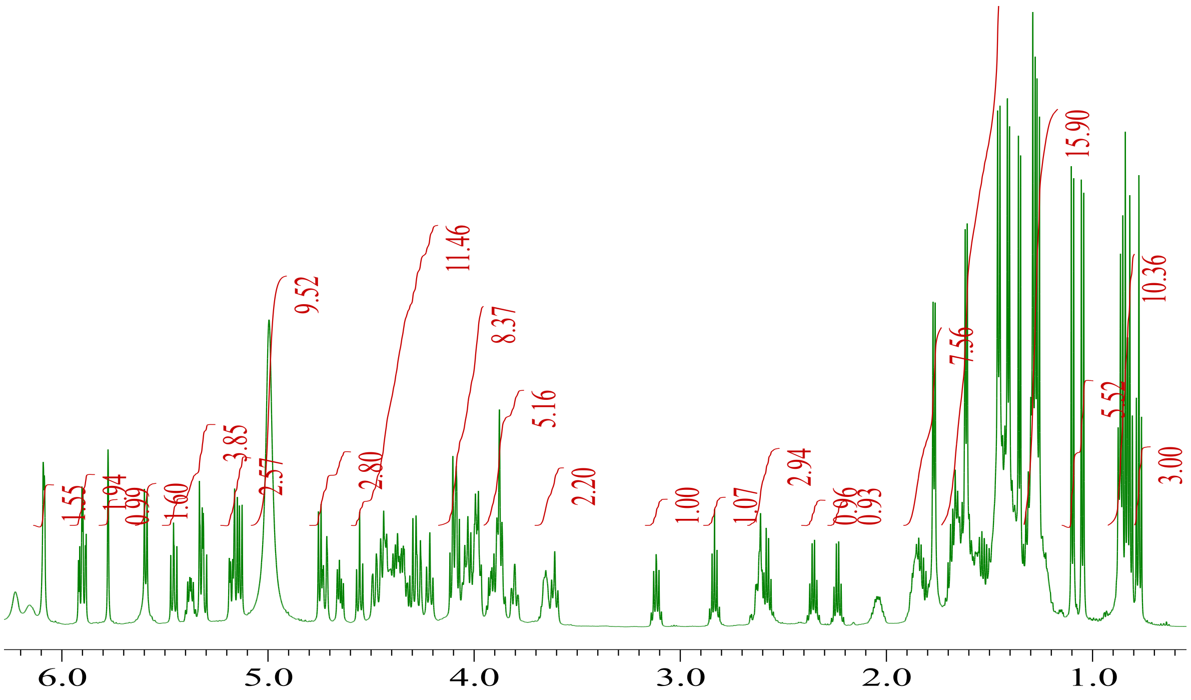

Figure S1: ^{1}H-NMR (600 MHz, pyridine-*d*$_5$) spectrum of **1**

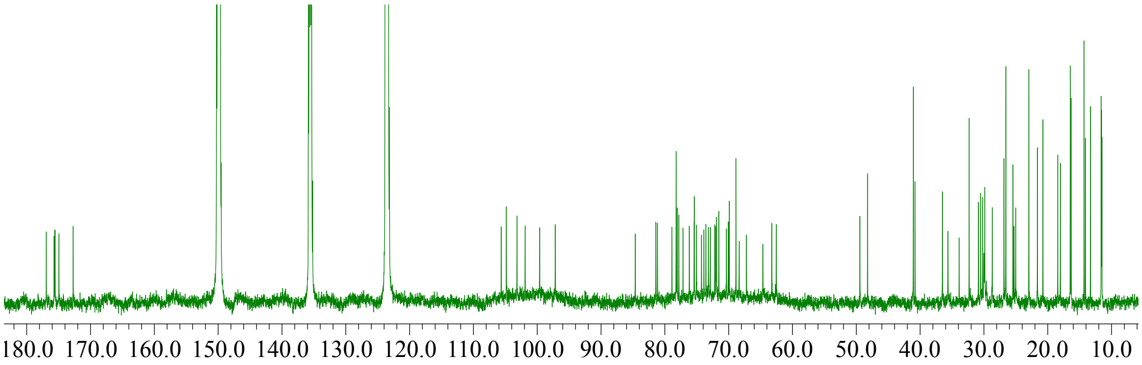

Figure S2: ^{13}C-NMR (150 MHz, pyridine-*d*$_5$) spectrum of **1**

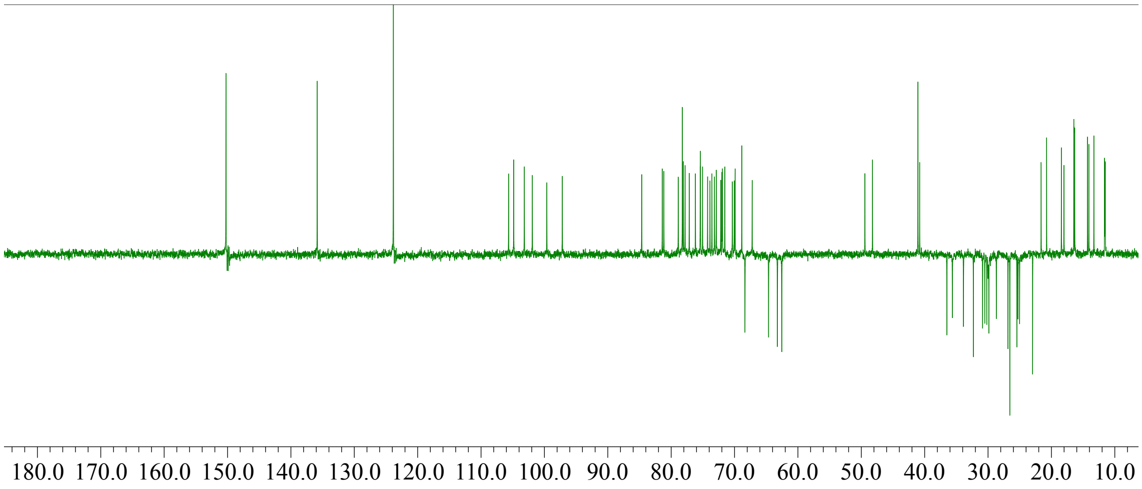

Figure S3: DEPT (150 MHz, pyridine-*d*$_5$) spectrum of **1**

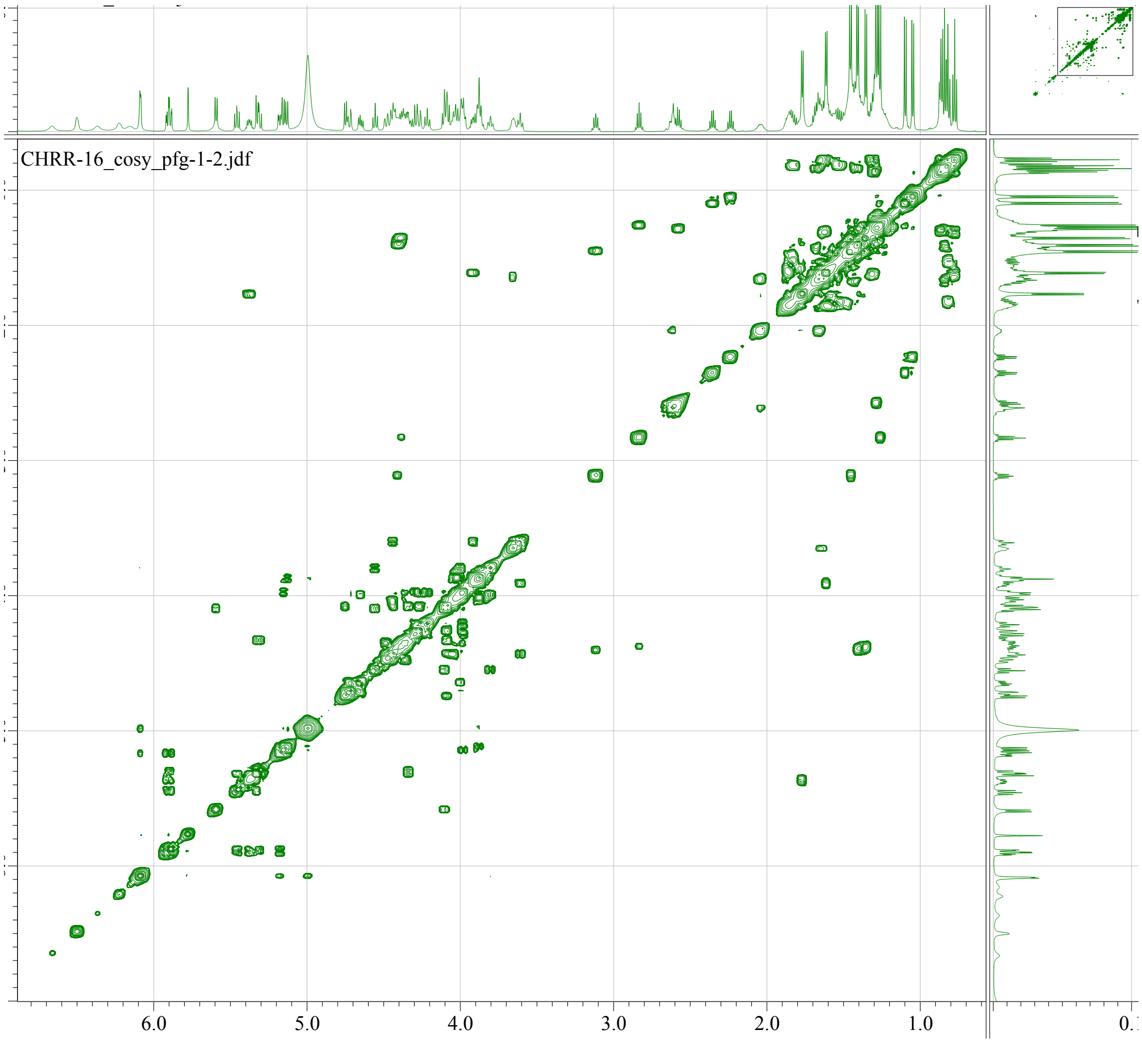

Figure S4: ^{1}H-^{1}H COSY (600 MHz, pyridine-d_5) spectrum of **1**

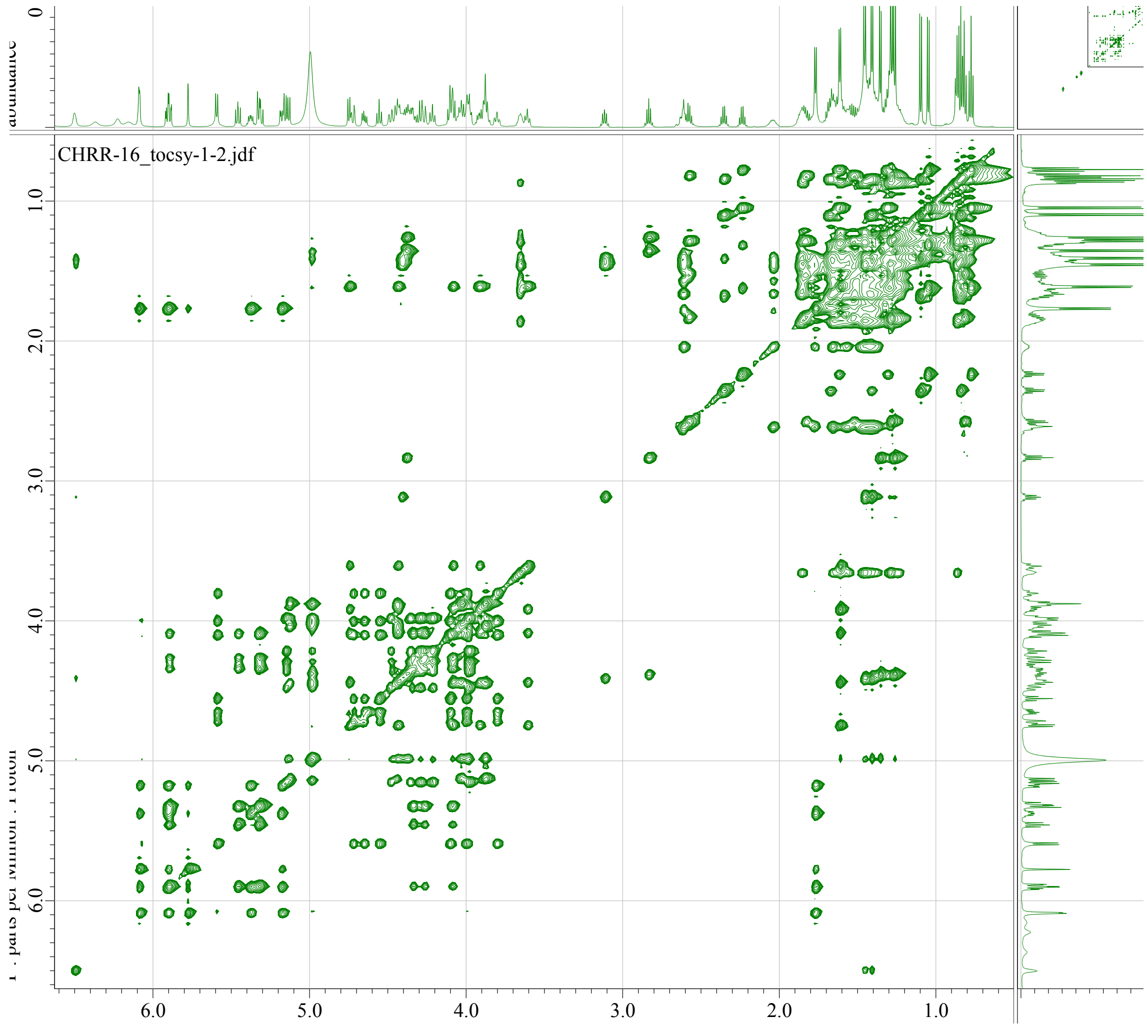

Figure S5: ^{1}H-^{1}H TOCSY (600 MHz, pyridine-d_5) spectrum of **1**

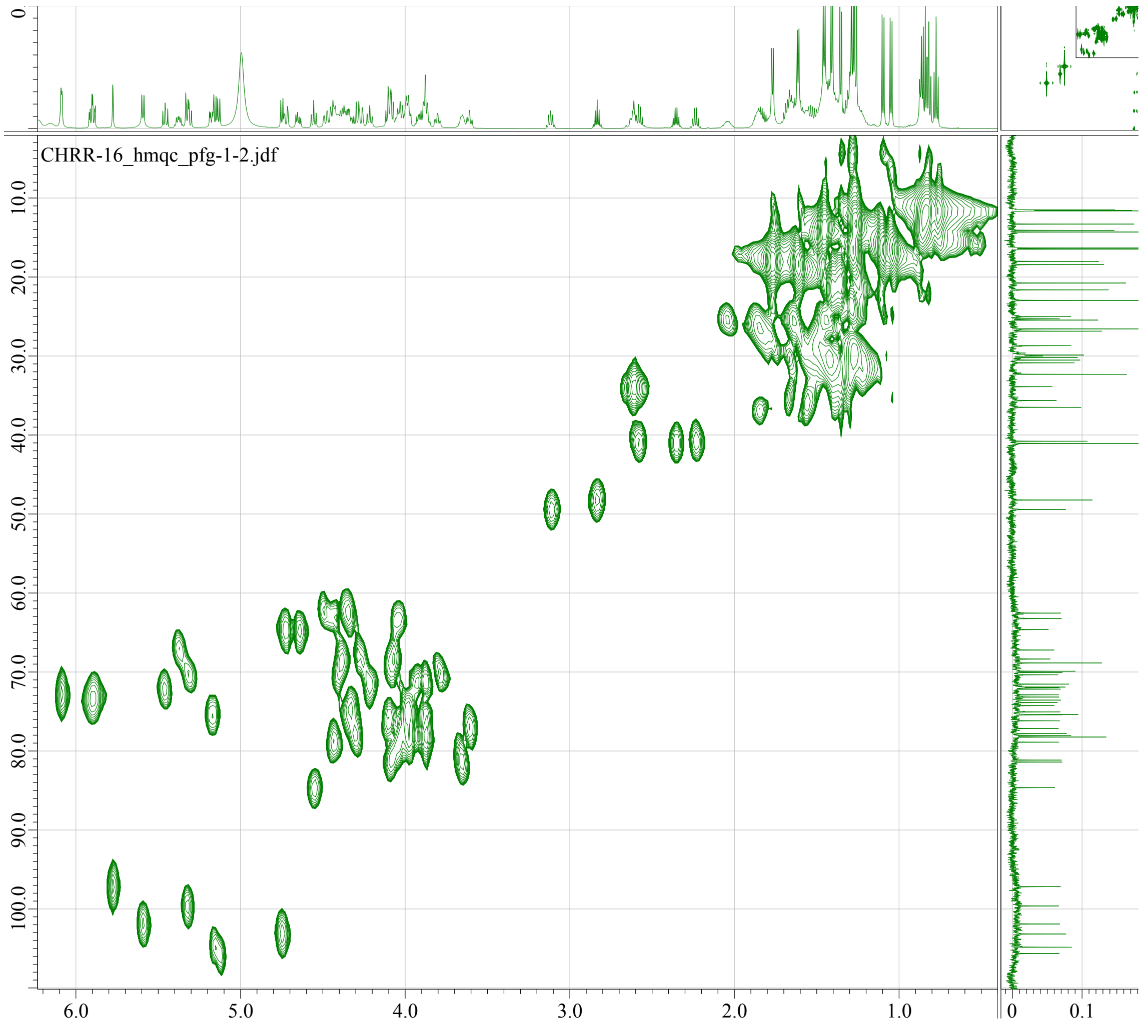

Figure S6: HMQC (600 MHz, pyridine-d_5) spectrum of **1**

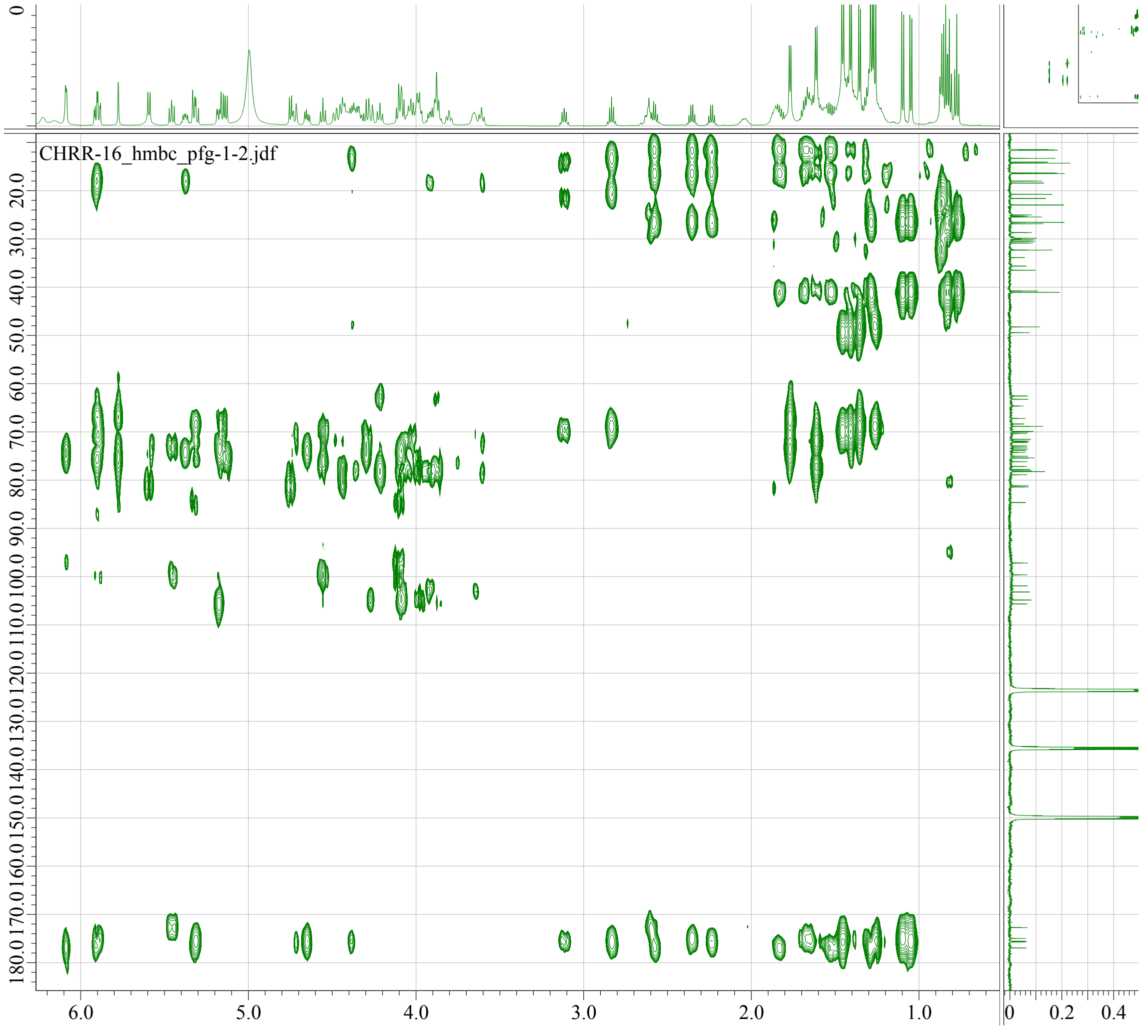

Figure S7: HMBC (600 MHz, pyridine-d_5) spectrum of **1**

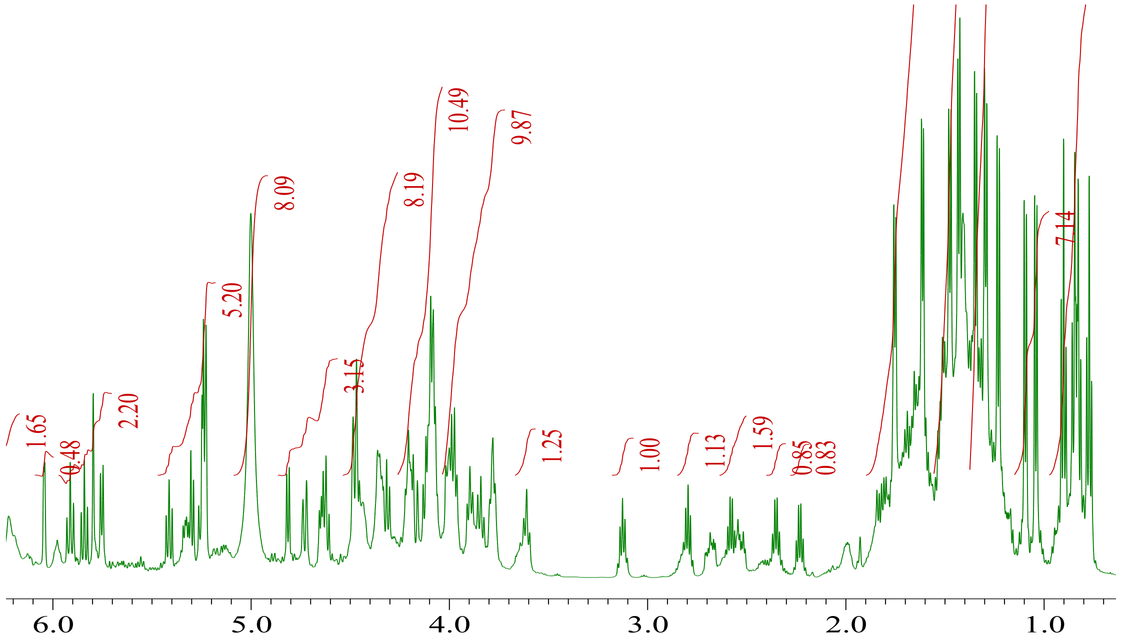

Figure S8: ^{1}H-NMR (600 MHz, pyridine-d_5) spectrum of **2**

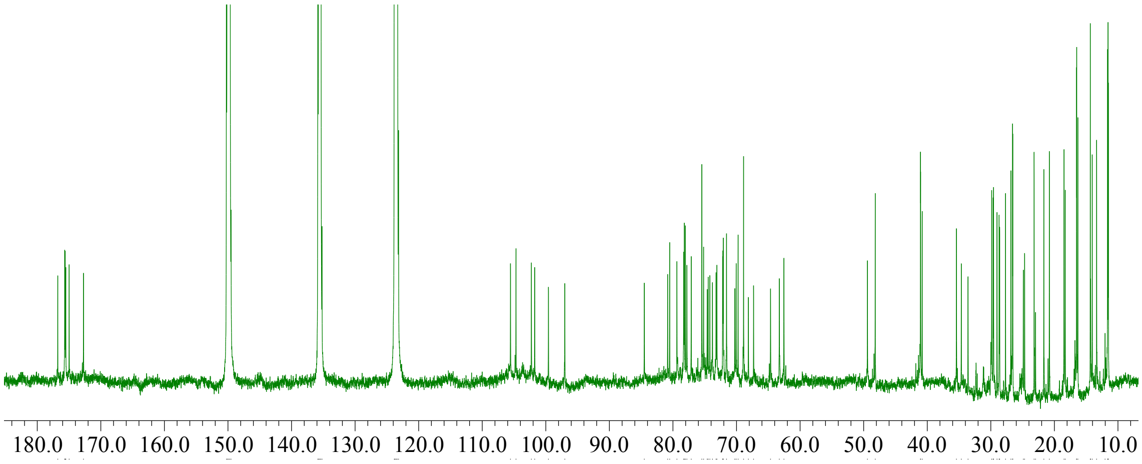

Figure S9: ^{13}C-NMR (150 MHz, pyridine-d_5) spectrum of **2**

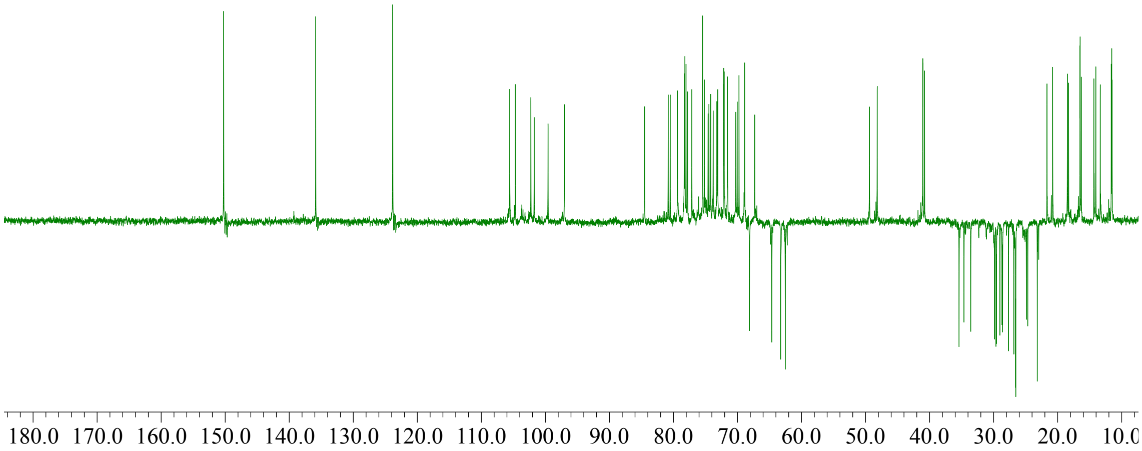

Figure S10: DEPT (150 MHz, pyridine-d_5) spectrum of **2**

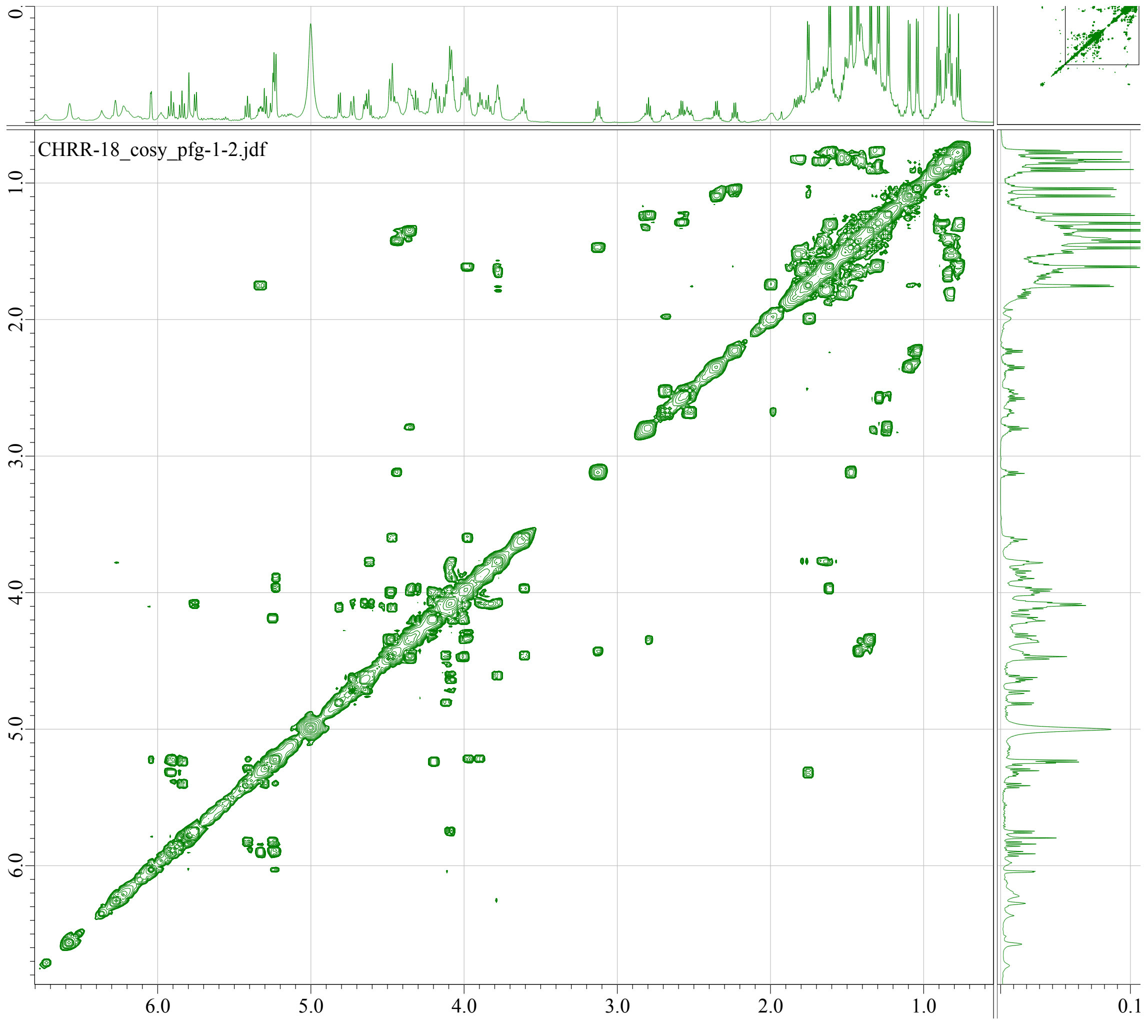

Figure S11: ^{1}H-^{1}H COSY (600 MHz, pyridine-d_5) spectrum of **2**

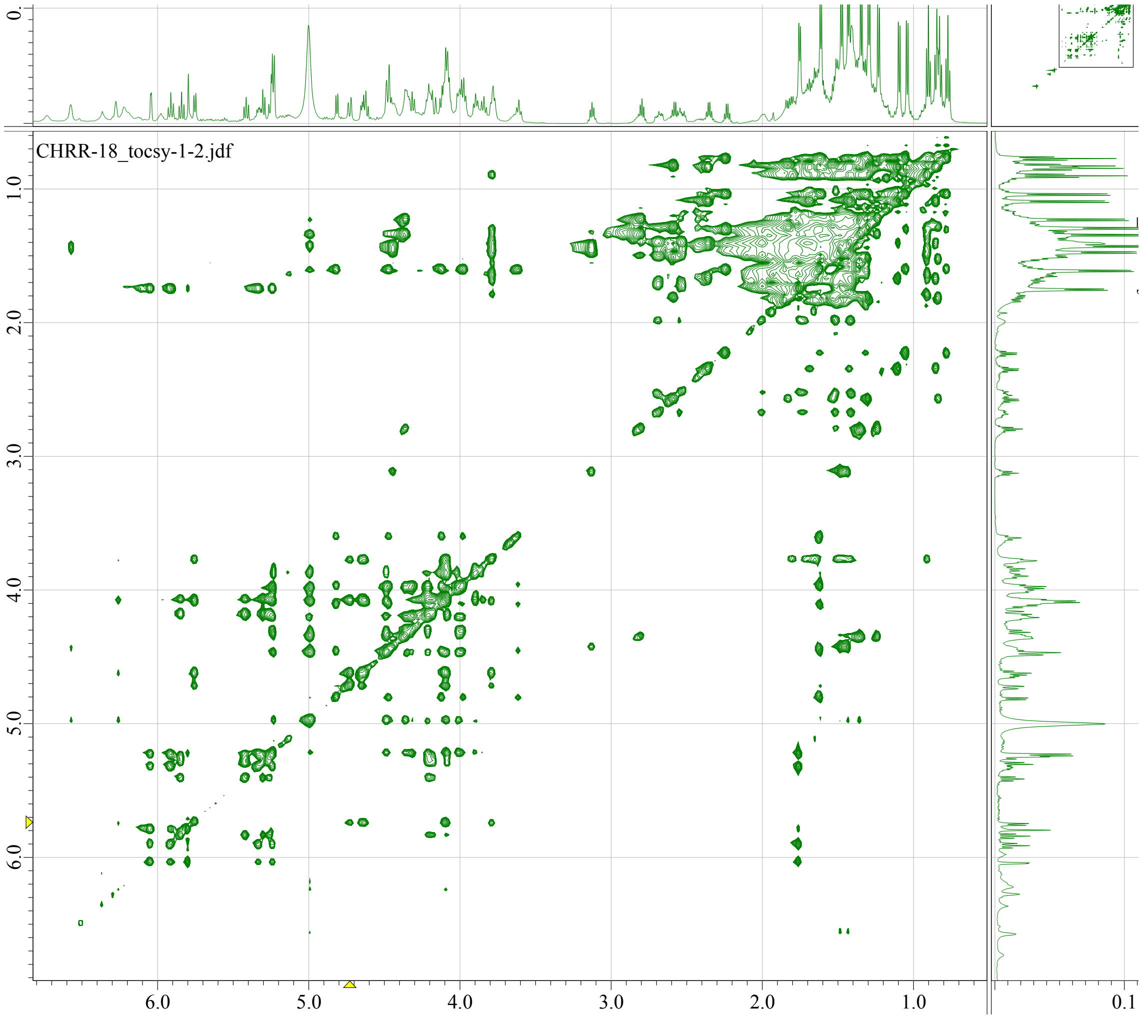

Figure S12: ^{1}H-^{1}H TOCSY (600 MHz, pyridine-d_5) spectrum of **2**

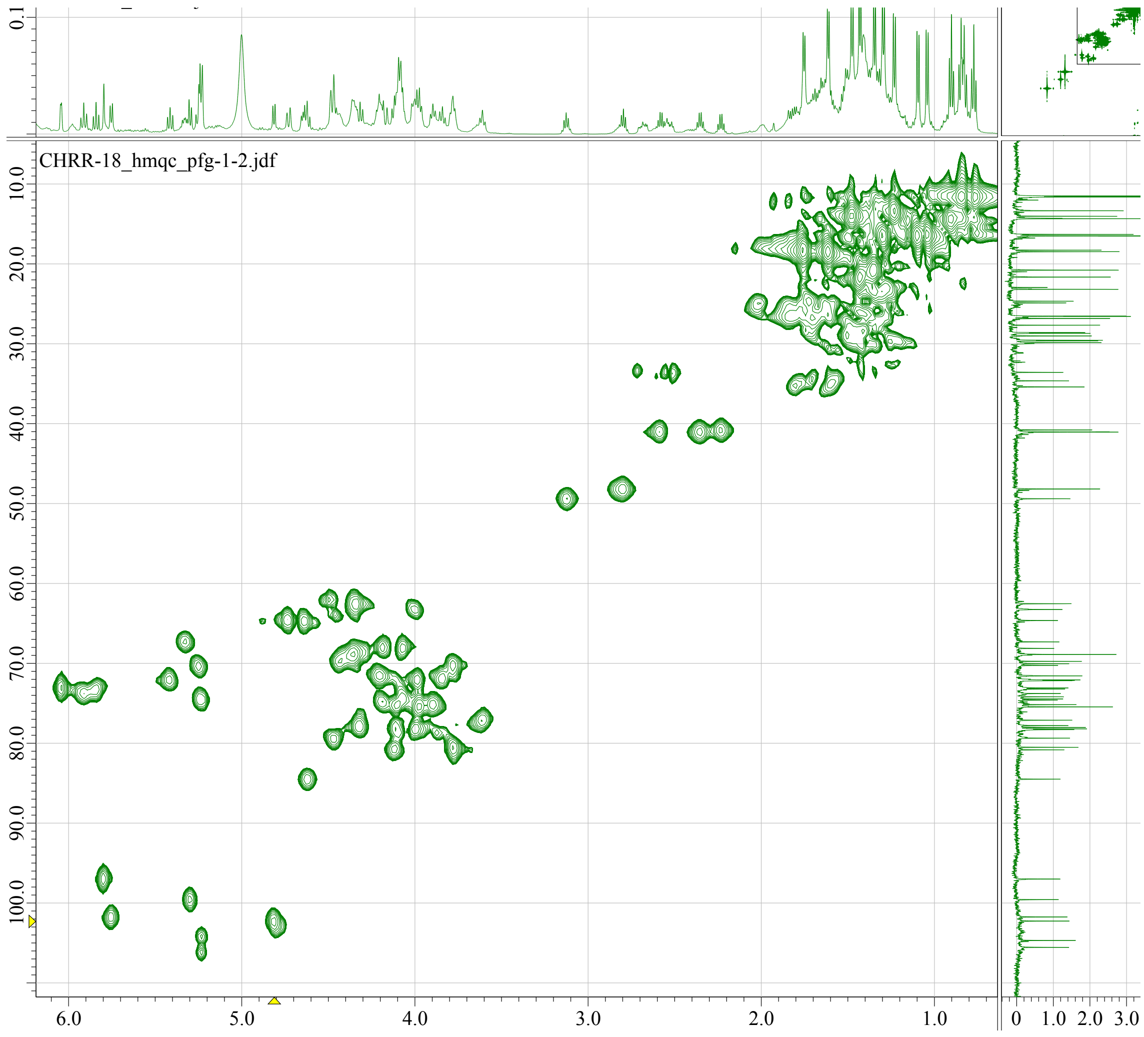

Figure S13: HMQC (600 MHz, pyridine-d_5) spectrum of **2**

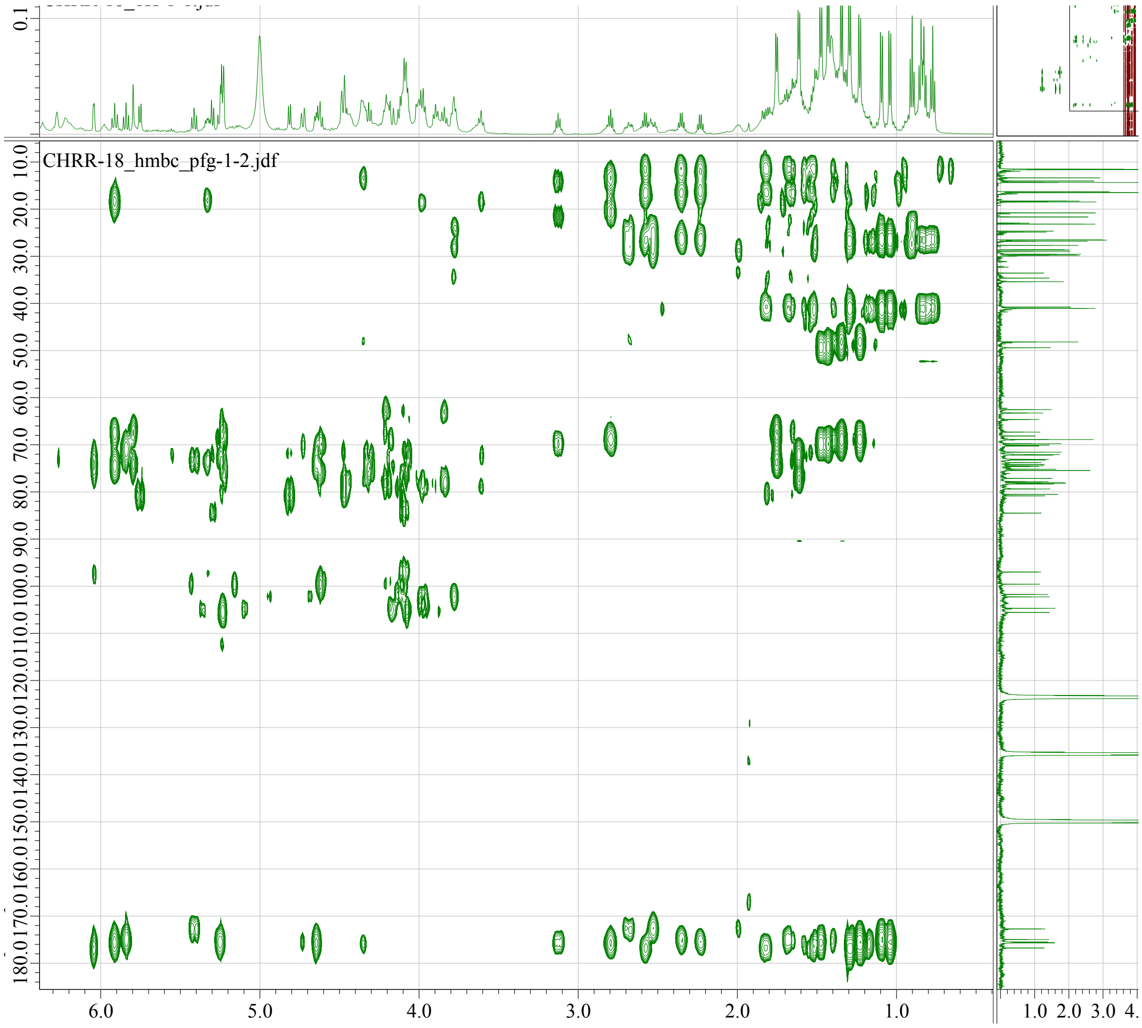

Figure S14: HMBC (600 MHz, pyridine-d_5) spectrum of **2**

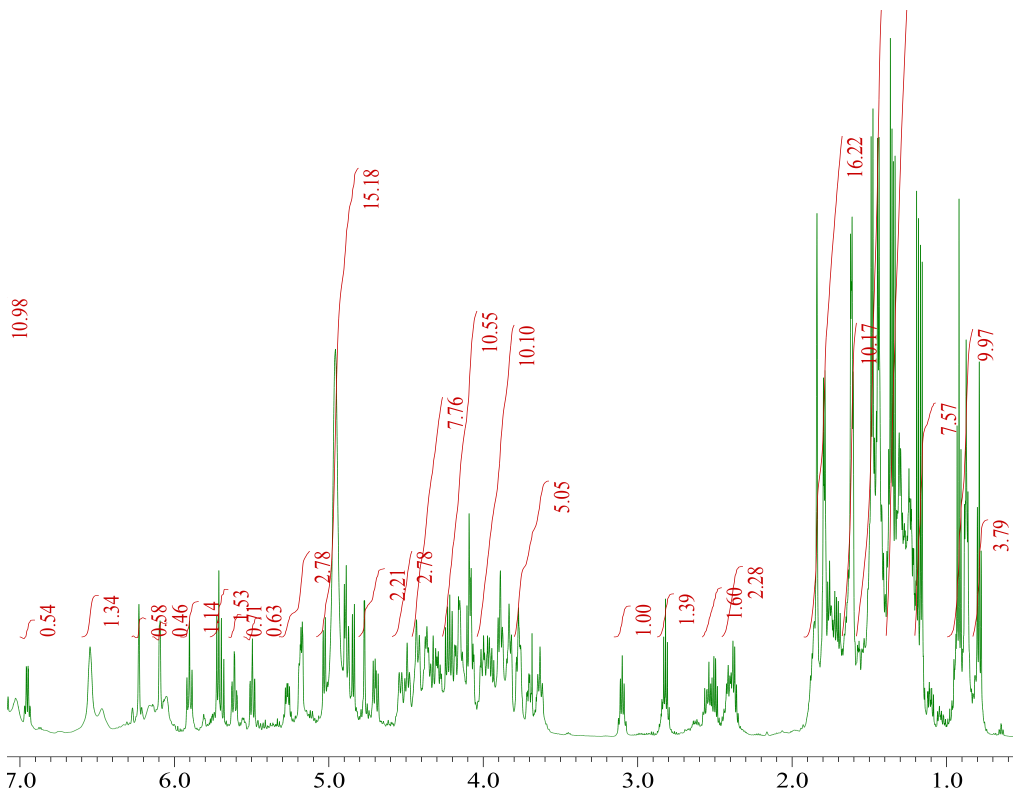

Figure S15: ^{1}H-NMR (600 MHz, pyridine-d_5) spectrum of **3**

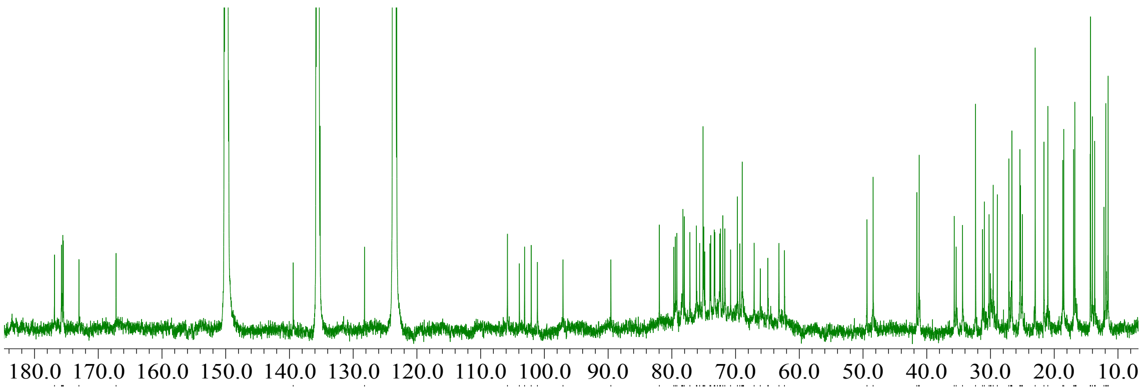

Figure S16: ^{13}C-NMR (150 MHz, pyridine-d_5) spectrum of **3**

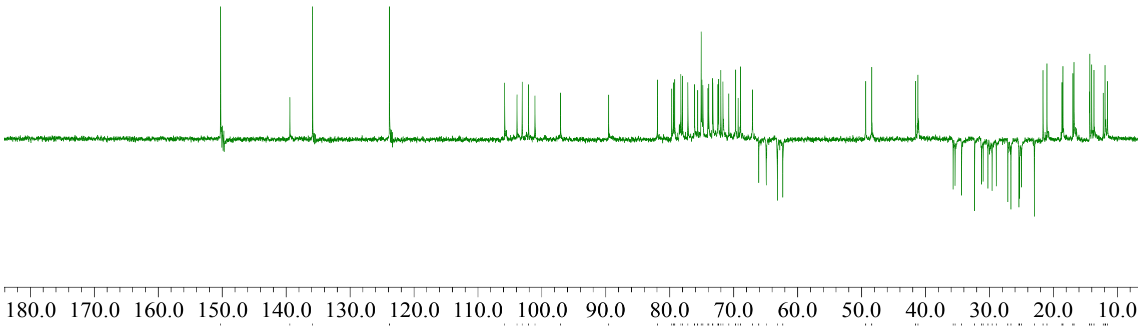

Figure S17: DEPT (150 MHz, pyridine-d_5) spectrum of **3**

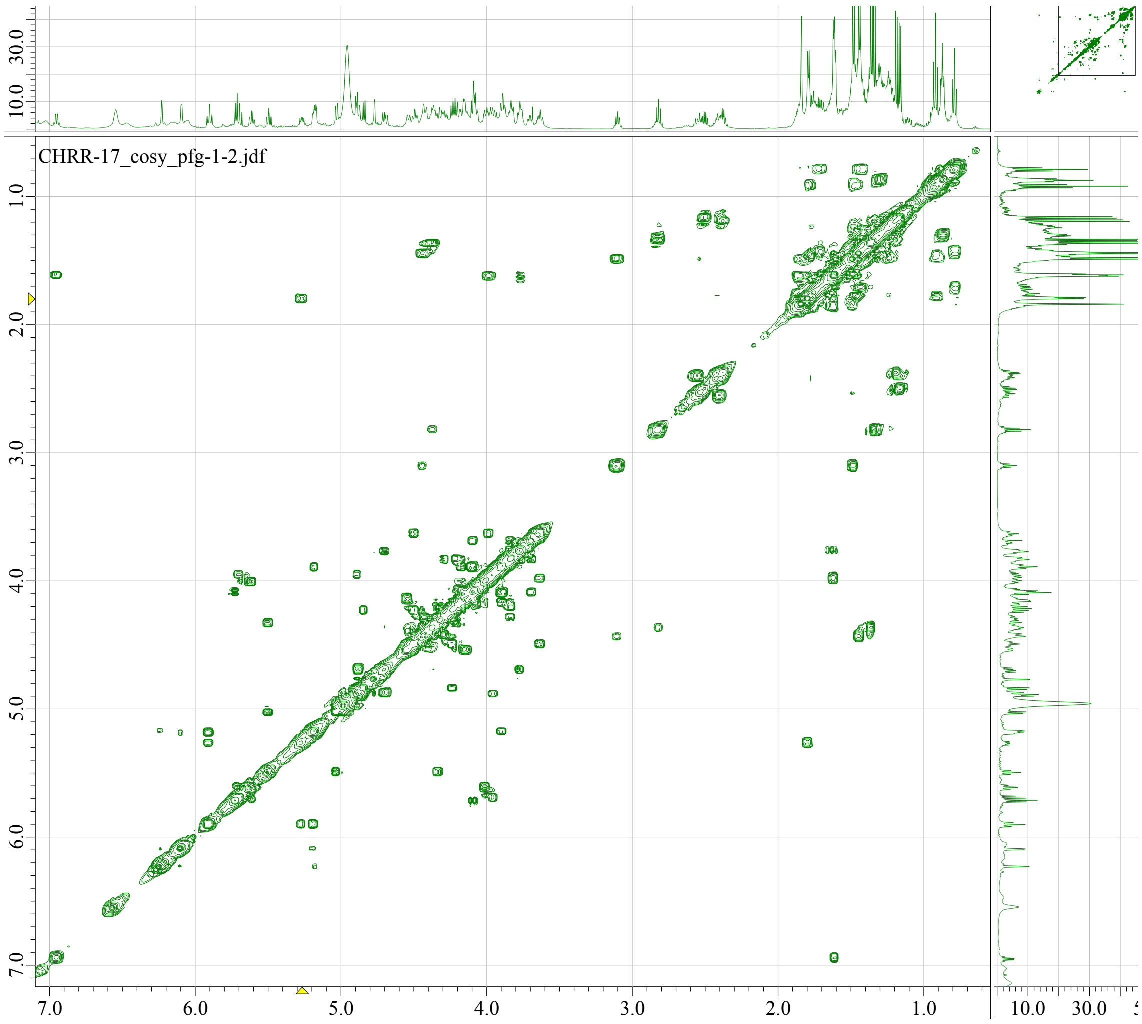

Figure S18: ^{1}H-^{1}H COSY (600 MHz, pyridine-d_5) spectrum of **3**

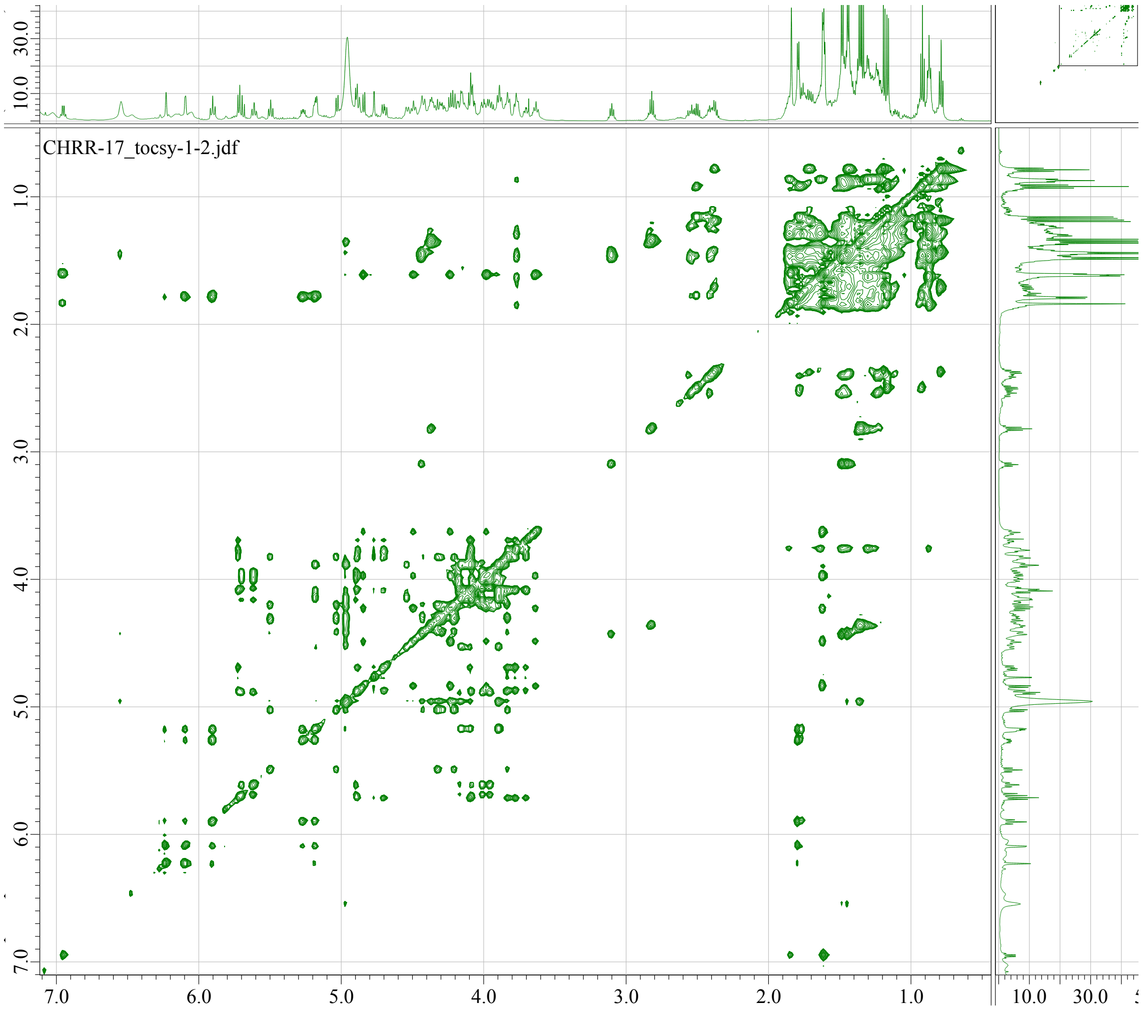

Figure S19: ^{1}H-^{1}H TOCSY (600 MHz, pyridine-d_5) spectrum of **3**

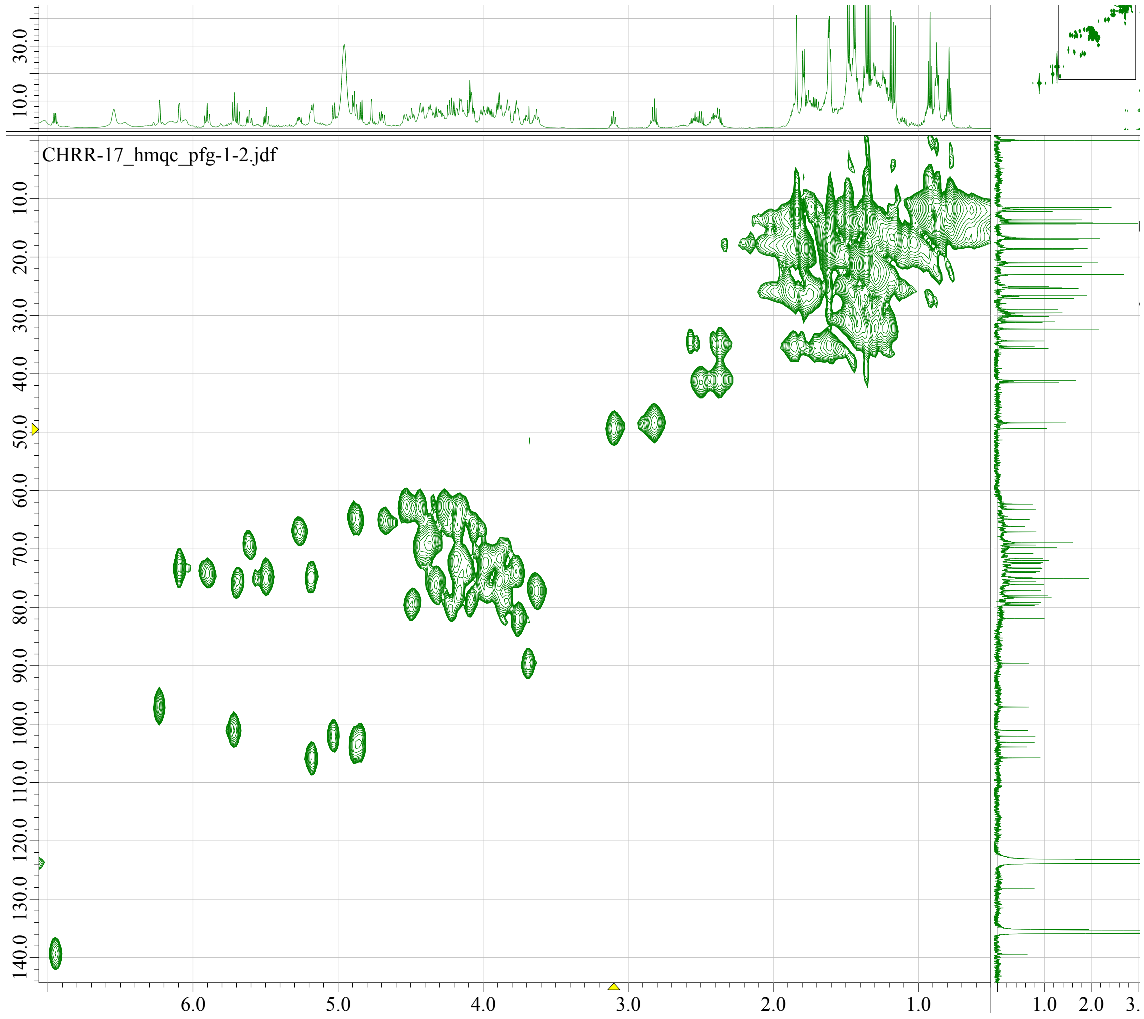

Figure S20: HMQC (600 MHz, pyridine-d_5) spectrum of **3**

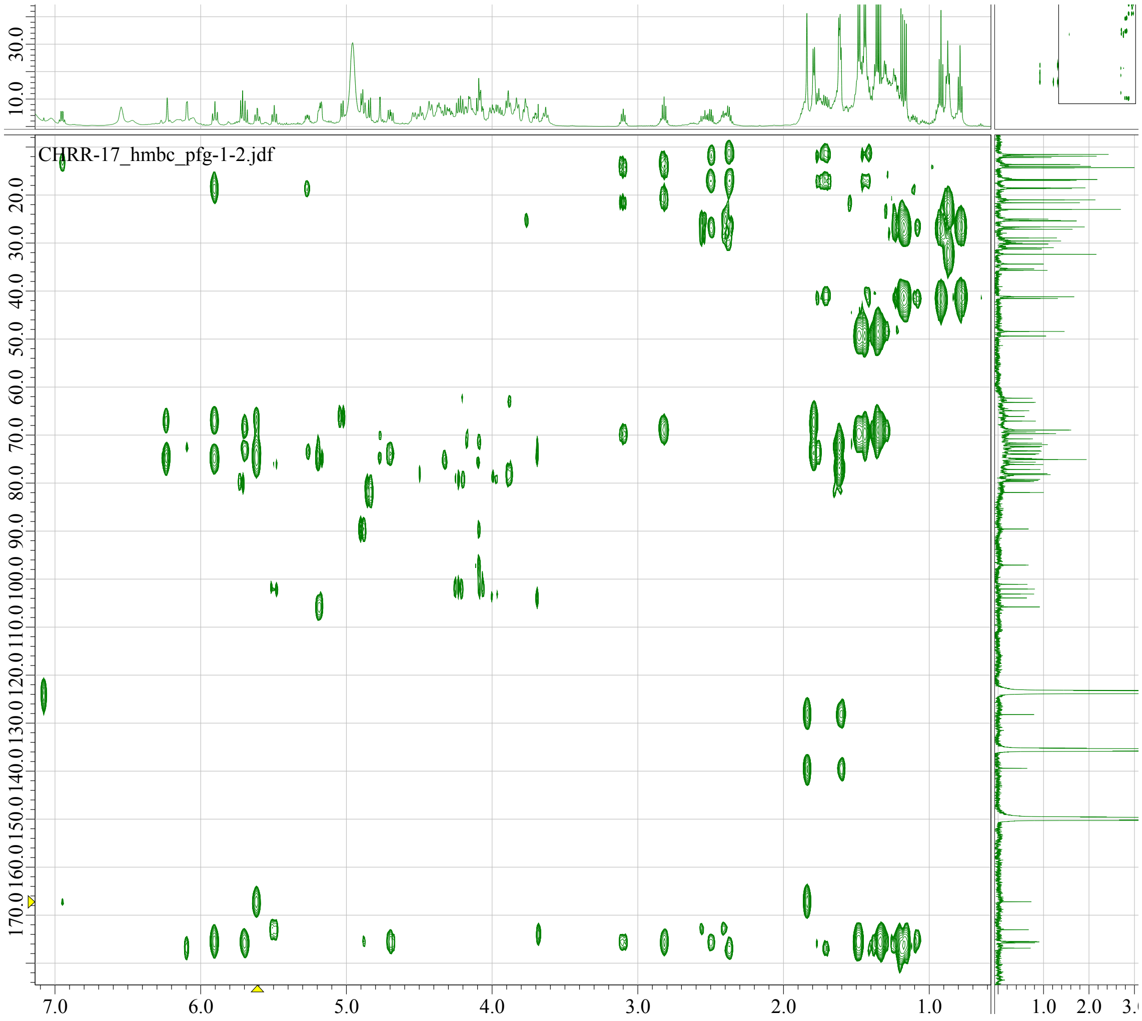

Figure S21: HMBC (600 MHz, pyridine-d_5) spectrum of **3**

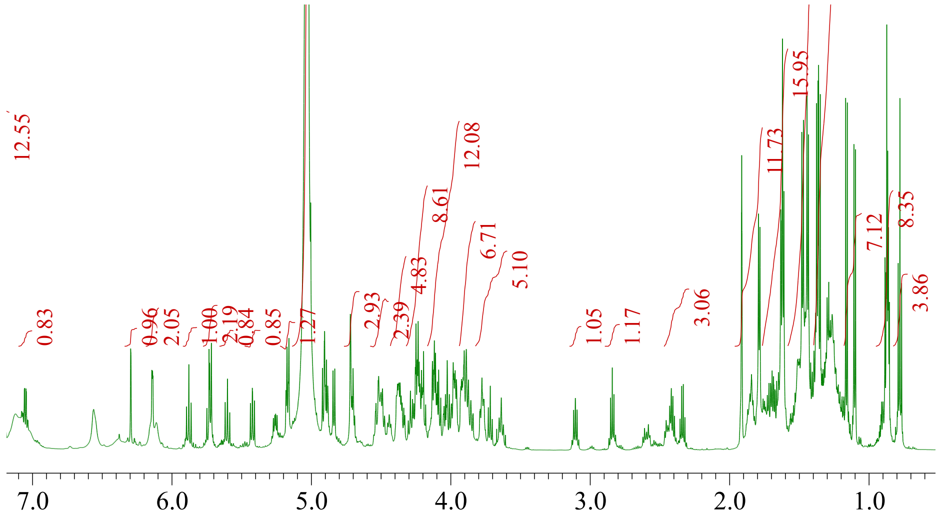

Figure S22: ^{1}H-NMR (600 MHz, pyridine-d_5) spectrum of **4**

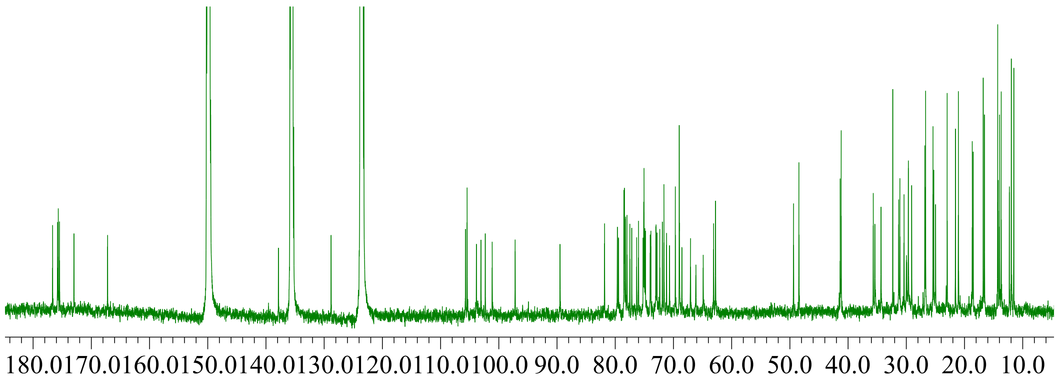

Figure S23: ^{13}C-NMR (150 MHz, pyridine-d_5) spectrum of **4**

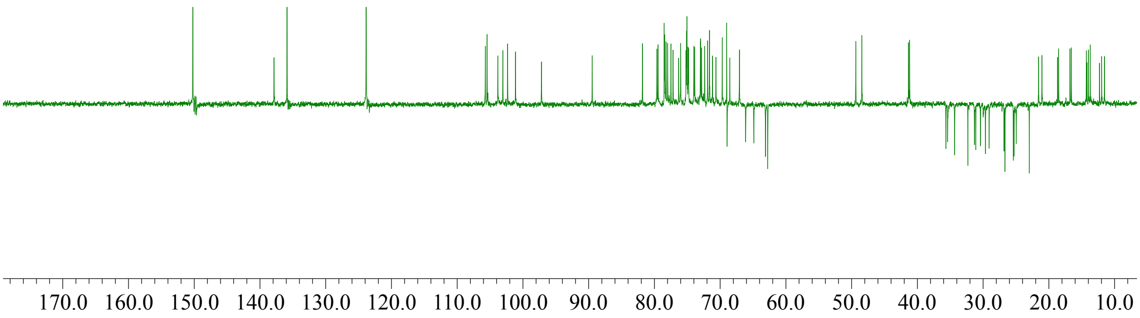

Figure S24: DEPT (150 MHz, pyridine-d_5) spectrum of **4**

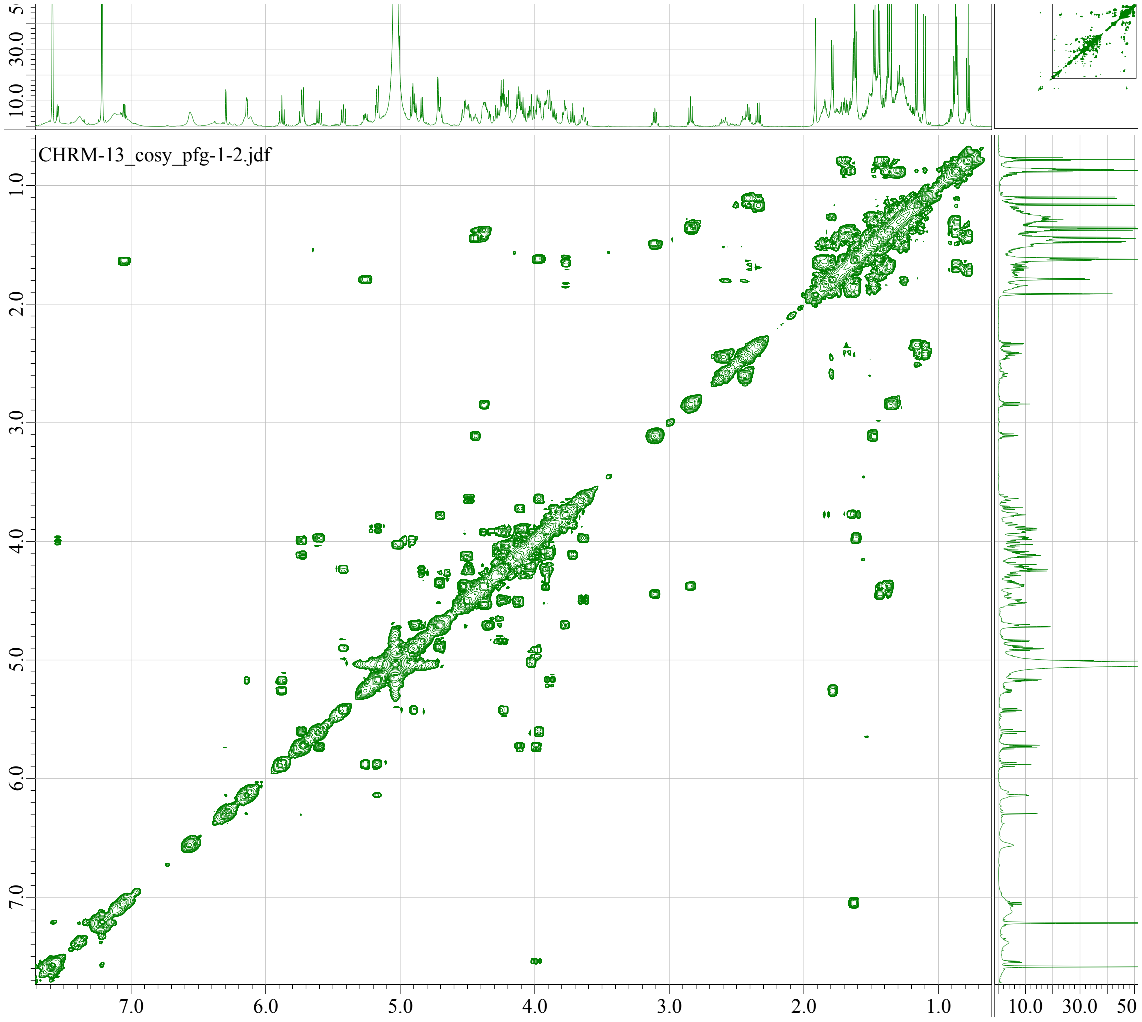

Figure S25: ^{1}H-^{1}H COSY (600 MHz, pyridine-d_5) spectrum of **4**

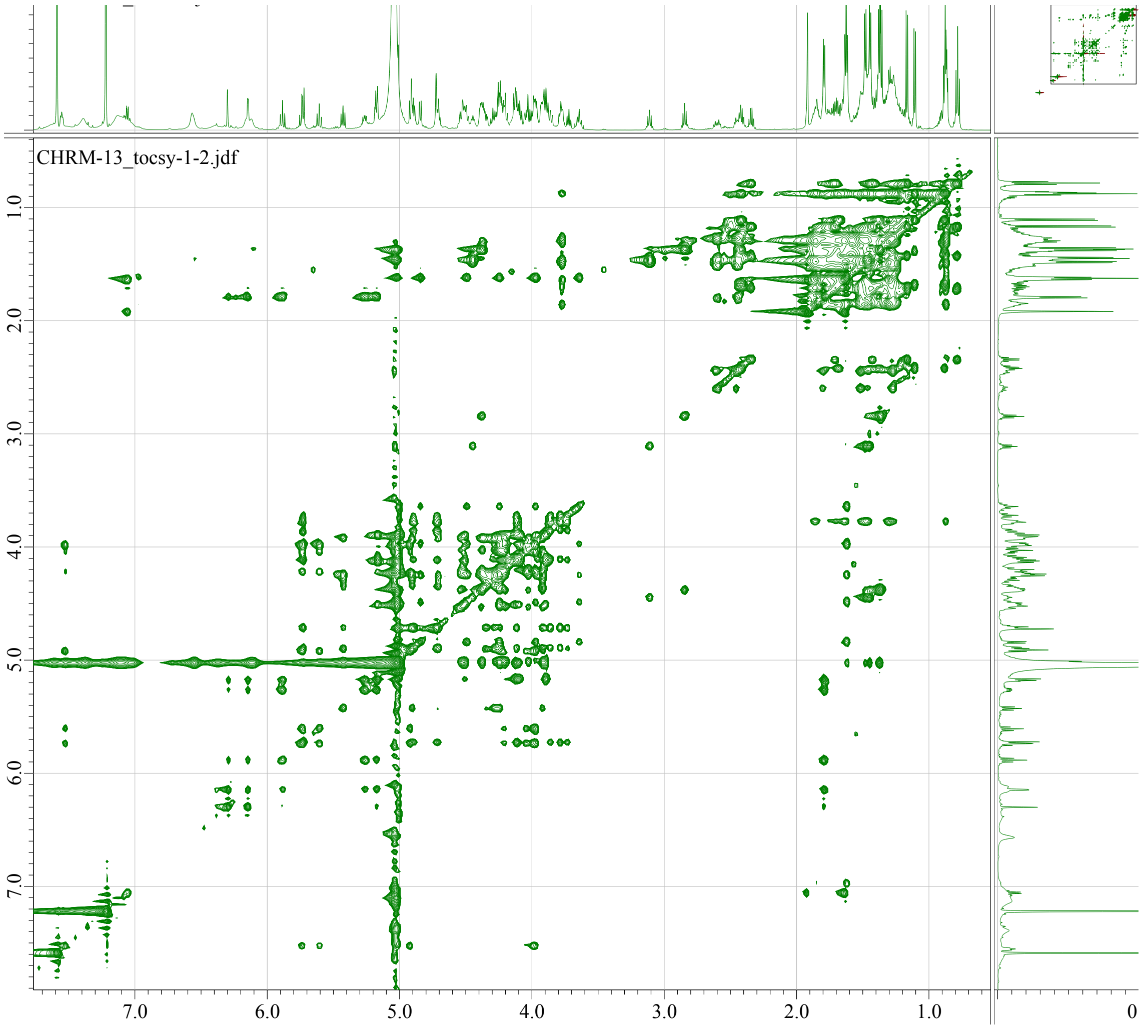

Figure S26: ^{1}H-^{1}H TOCSY (600 MHz, pyridine-d_5) spectrum of **4**

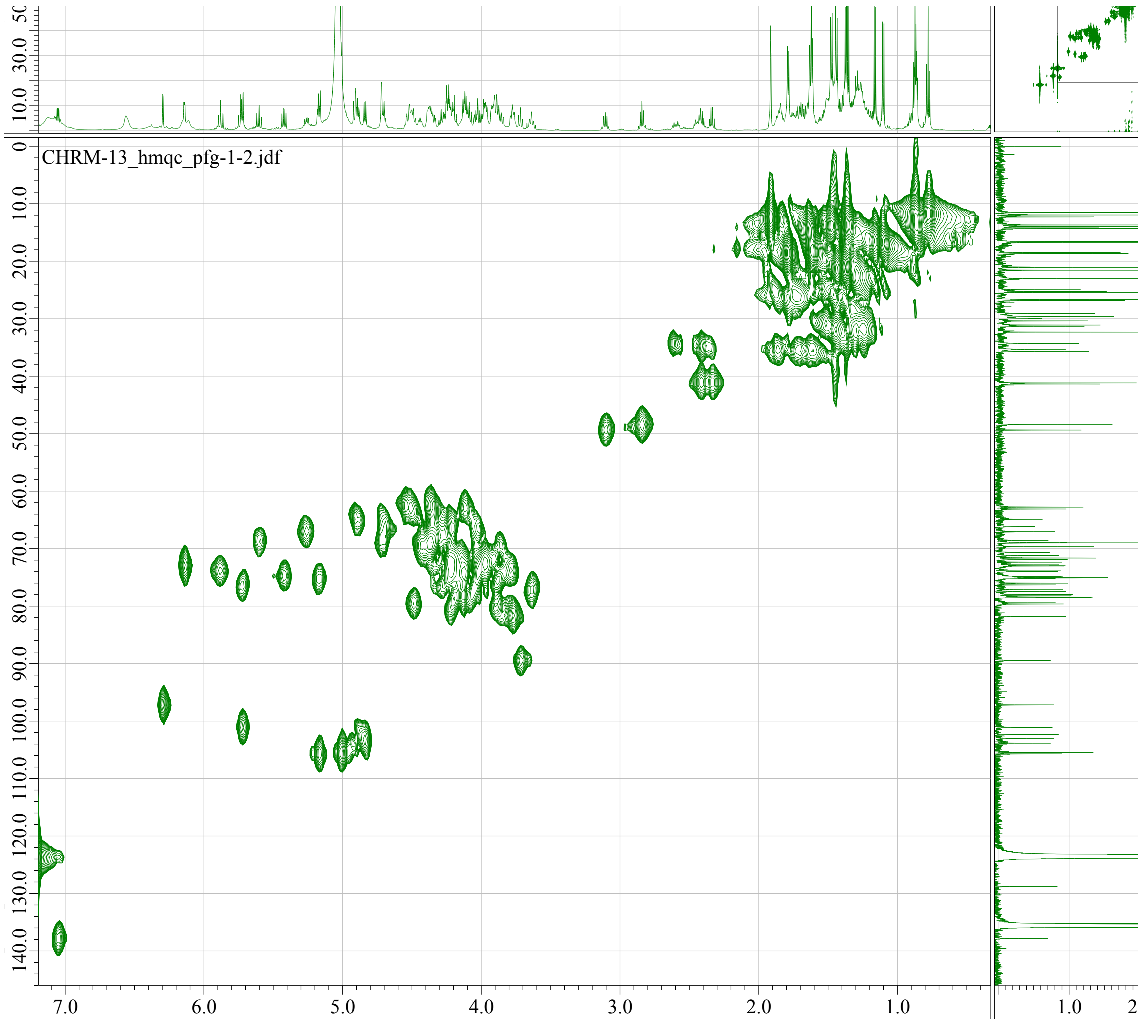

Figure S27: HMQC (600 MHz, pyridine-d_5) spectrum of **4**

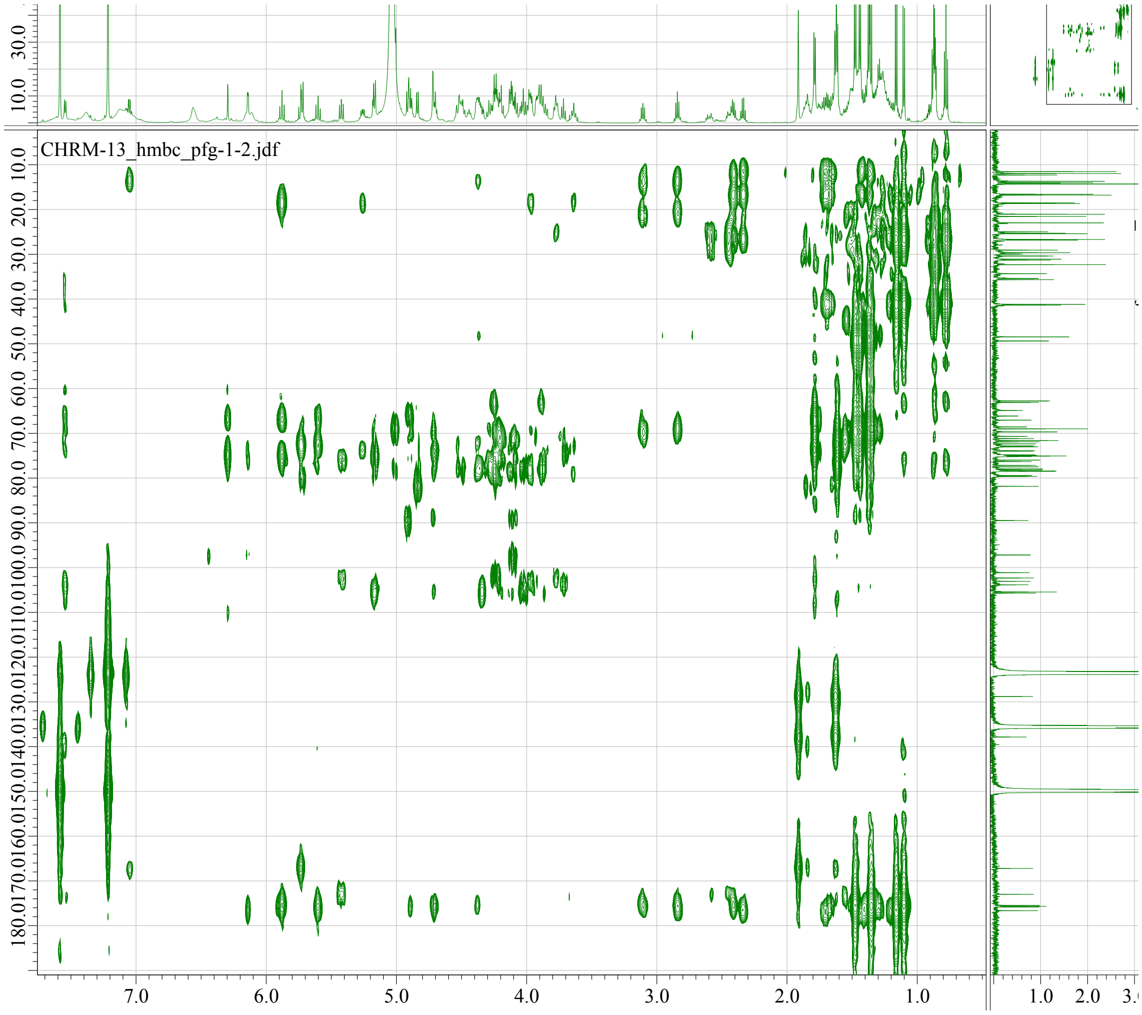

Figure S28: HMBC (600 MHz, pyridine-d_5) spectrum of **4**

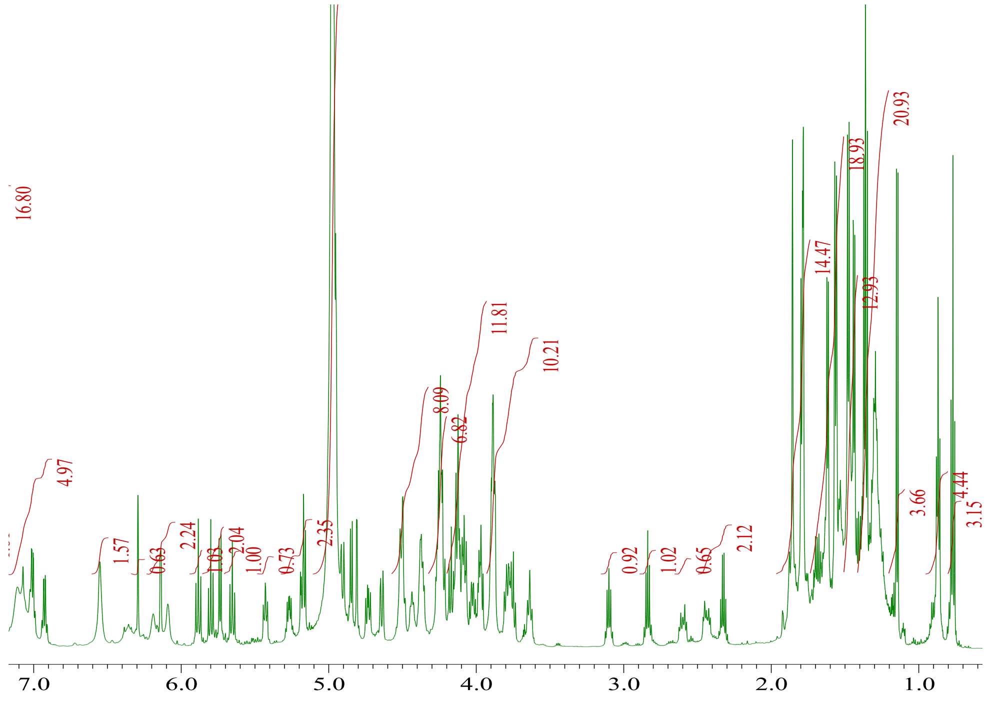

Figure S29: ^{1}H-NMR (600 MHz, pyridine-d_5) spectrum of **5**

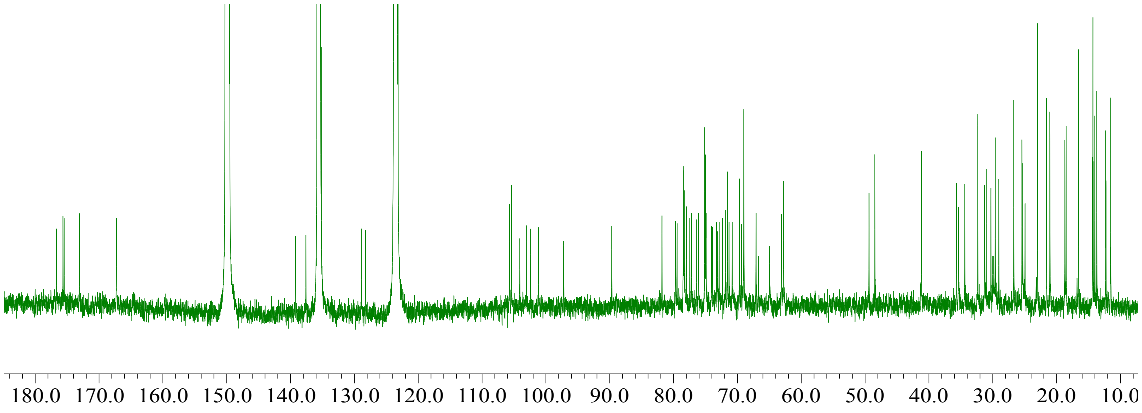

Figure S30: ^{13}C-NMR (150 MHz, pyridine-d_5) spectrum of **5**

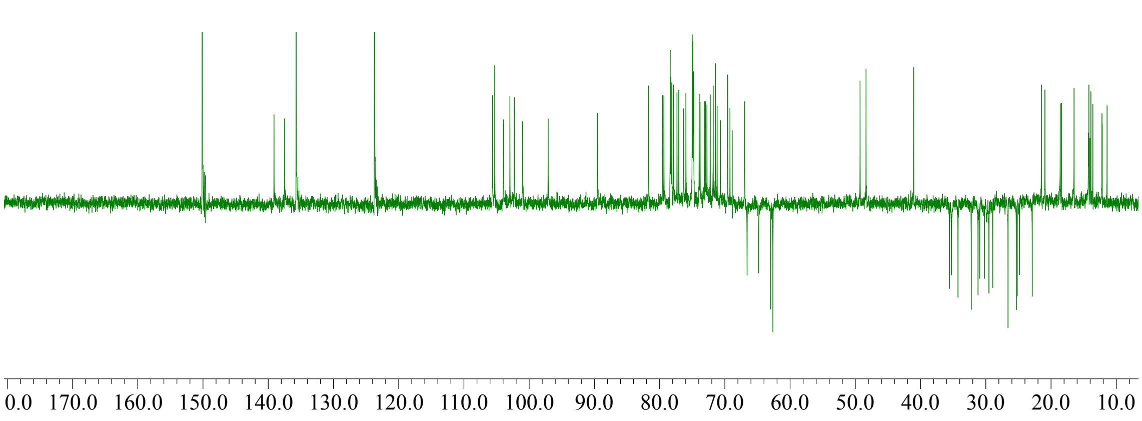

Figure S31: DEPT (150 MHz, pyridine-d_5) spectrum of **5**

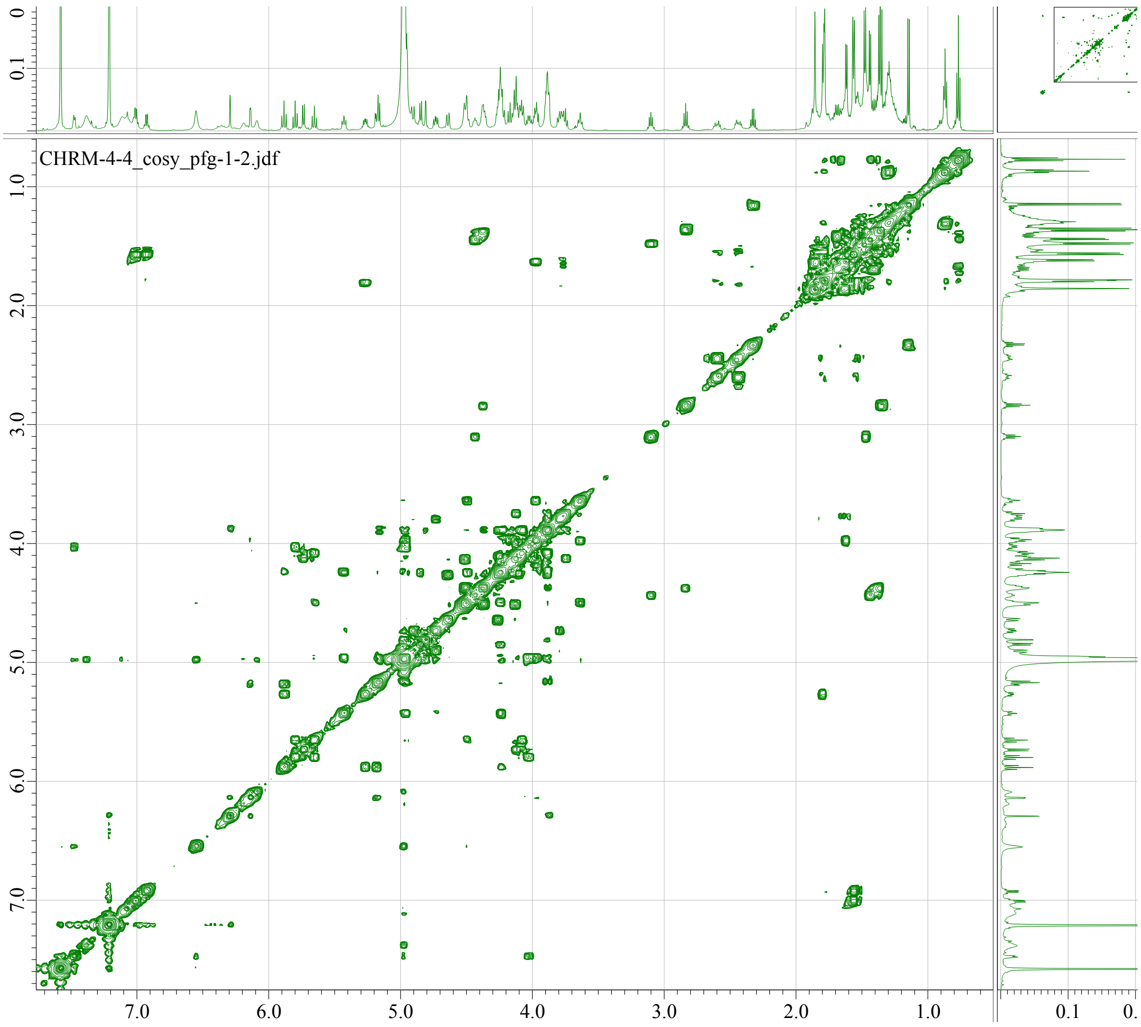

Figure S32: ^{1}H-^{1}H COSY (600 MHz, pyridine-d_5) spectrum of **5**

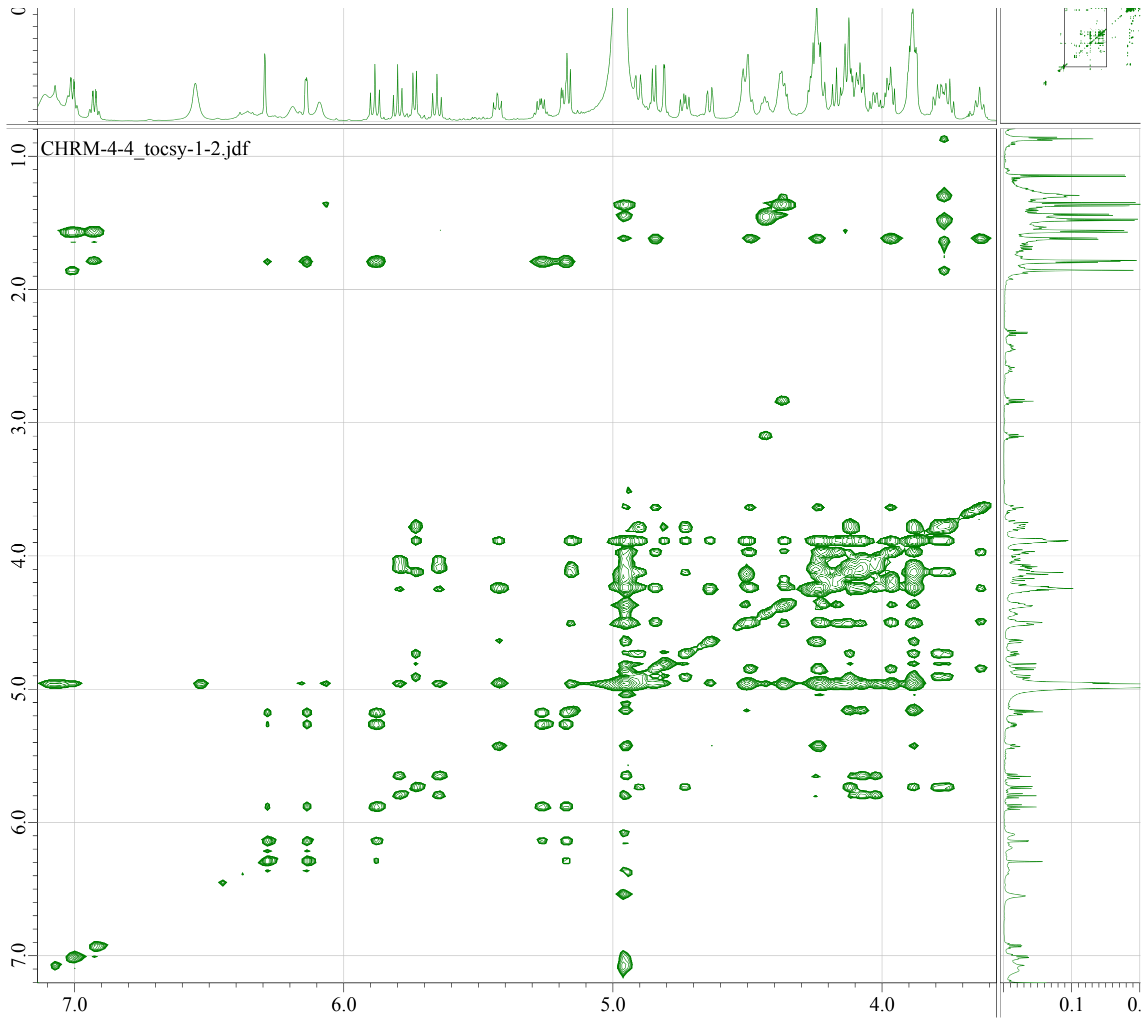

Figure S33: ^{1}H-^{1}H TOCSY (600 MHz, pyridine-d_5) spectrum of **5**

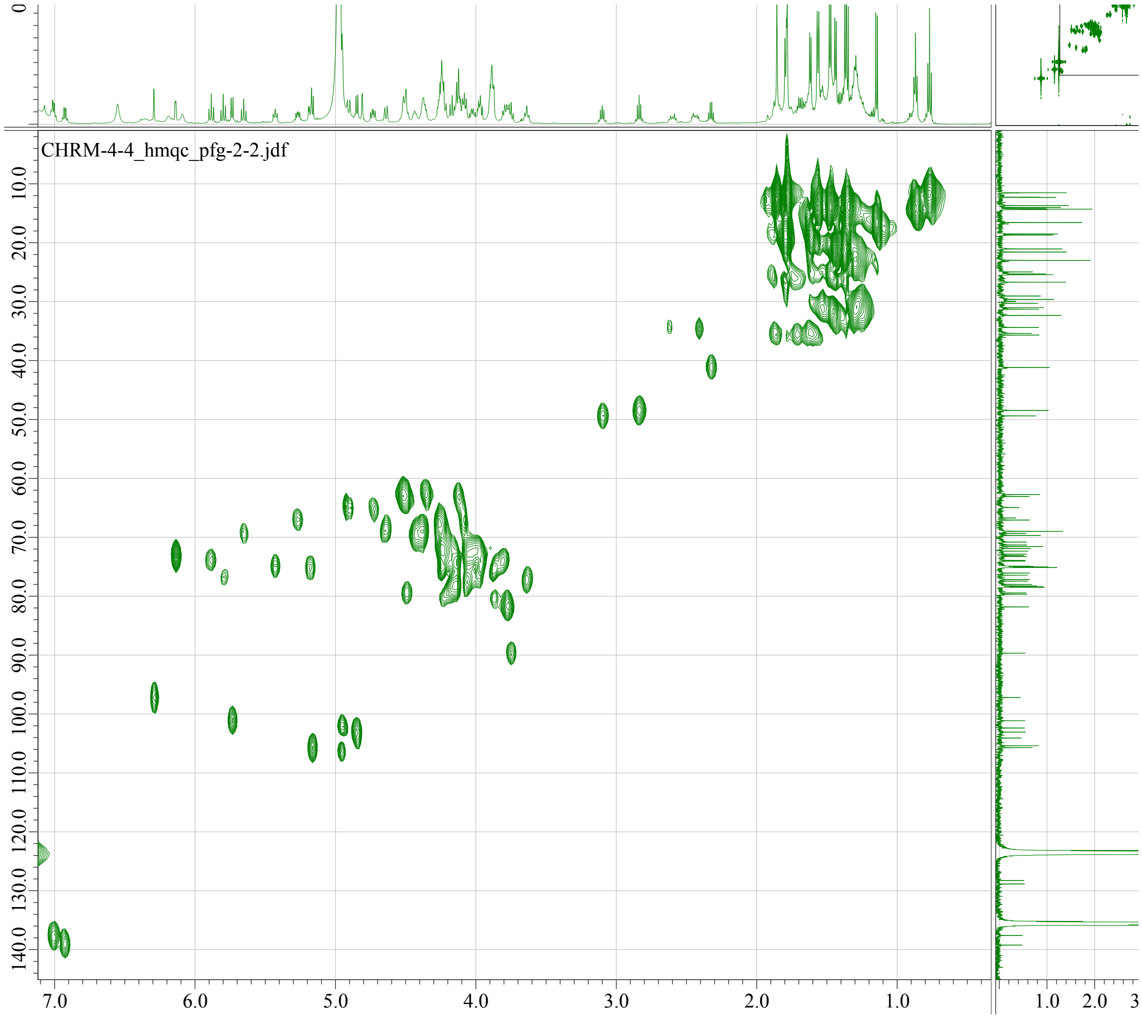

Figure S34: HMQC (600 MHz, pyridine-d_5) spectrum of **5**

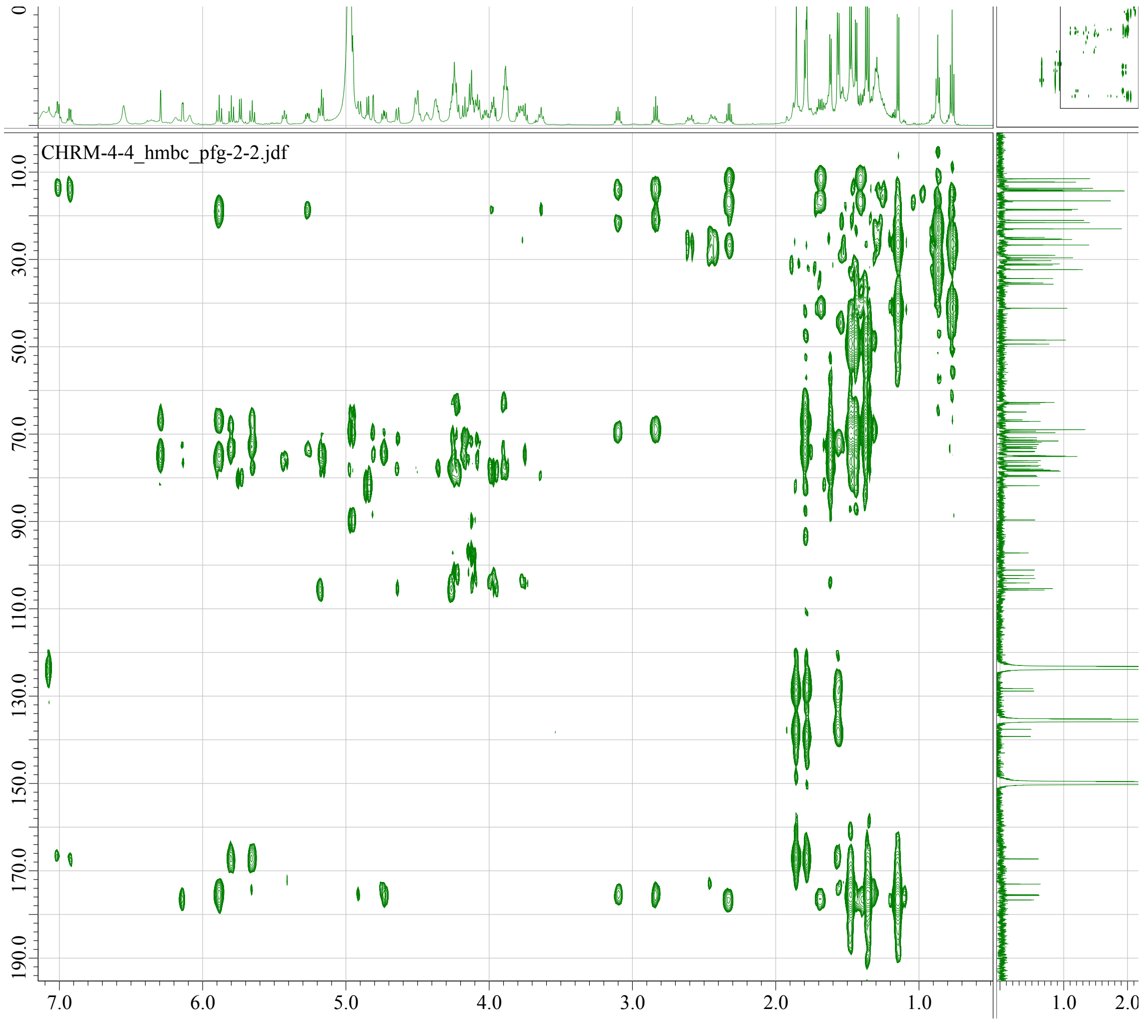

Figure S35: HMBC (600 MHz, pyridine-d_5) spectrum of **5**

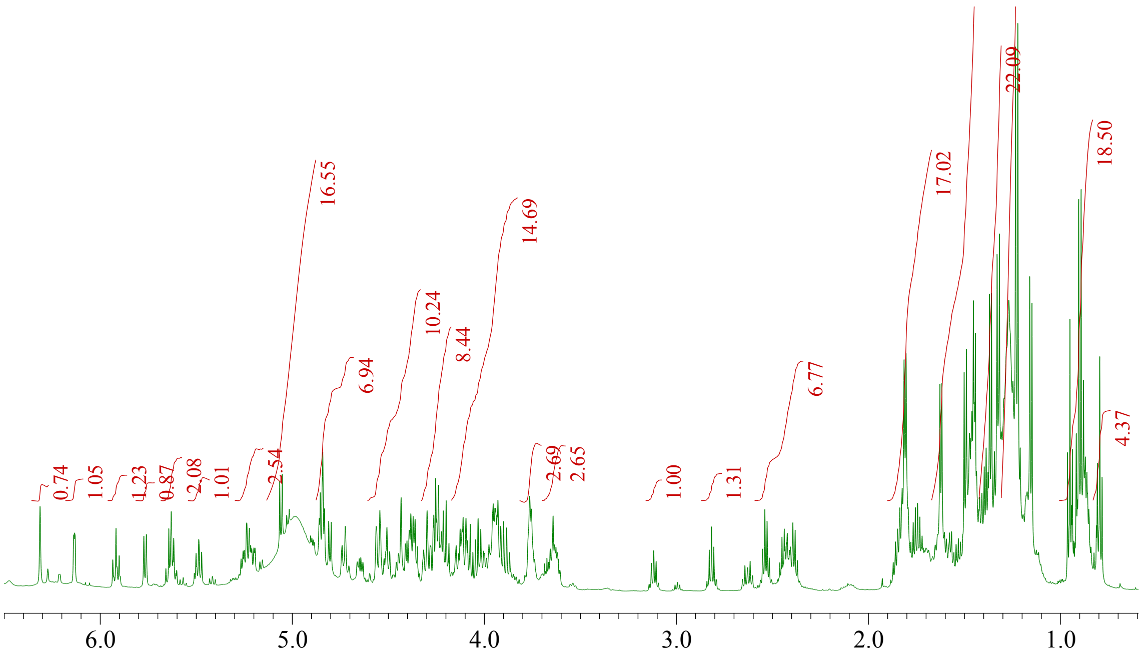

Figure S36: ^{1}H-NMR (600 MHz, pyridine-d_5) spectrum of **6**

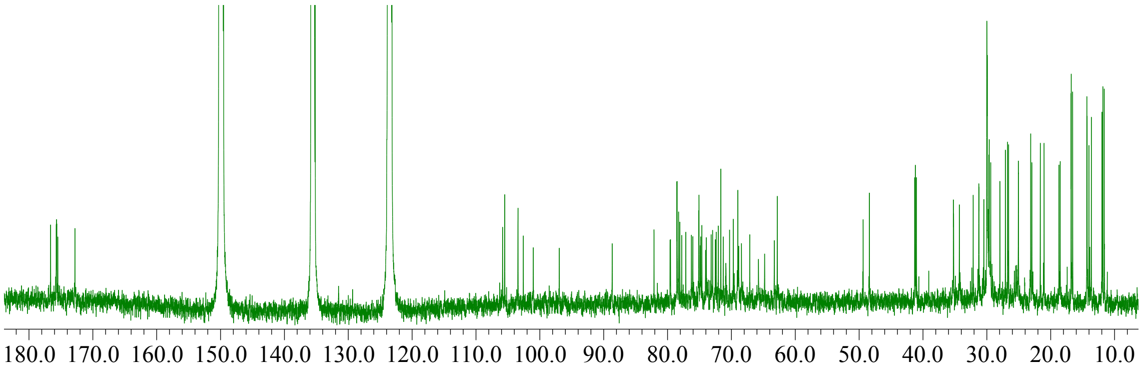

Figure S37: ^{13}C-NMR (150 MHz, pyridine-d_5) spectrum of **6**

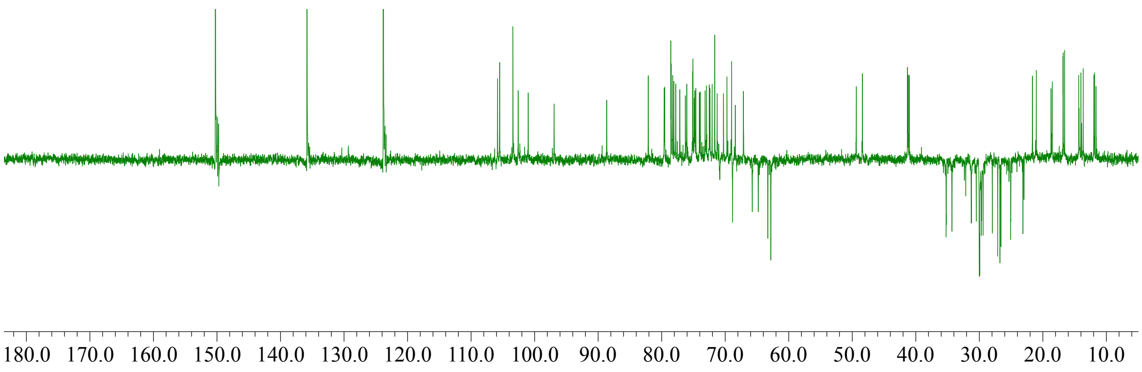

Figure S38: DEPT (150 MHz, pyridine-d_5) spectrum of **6**

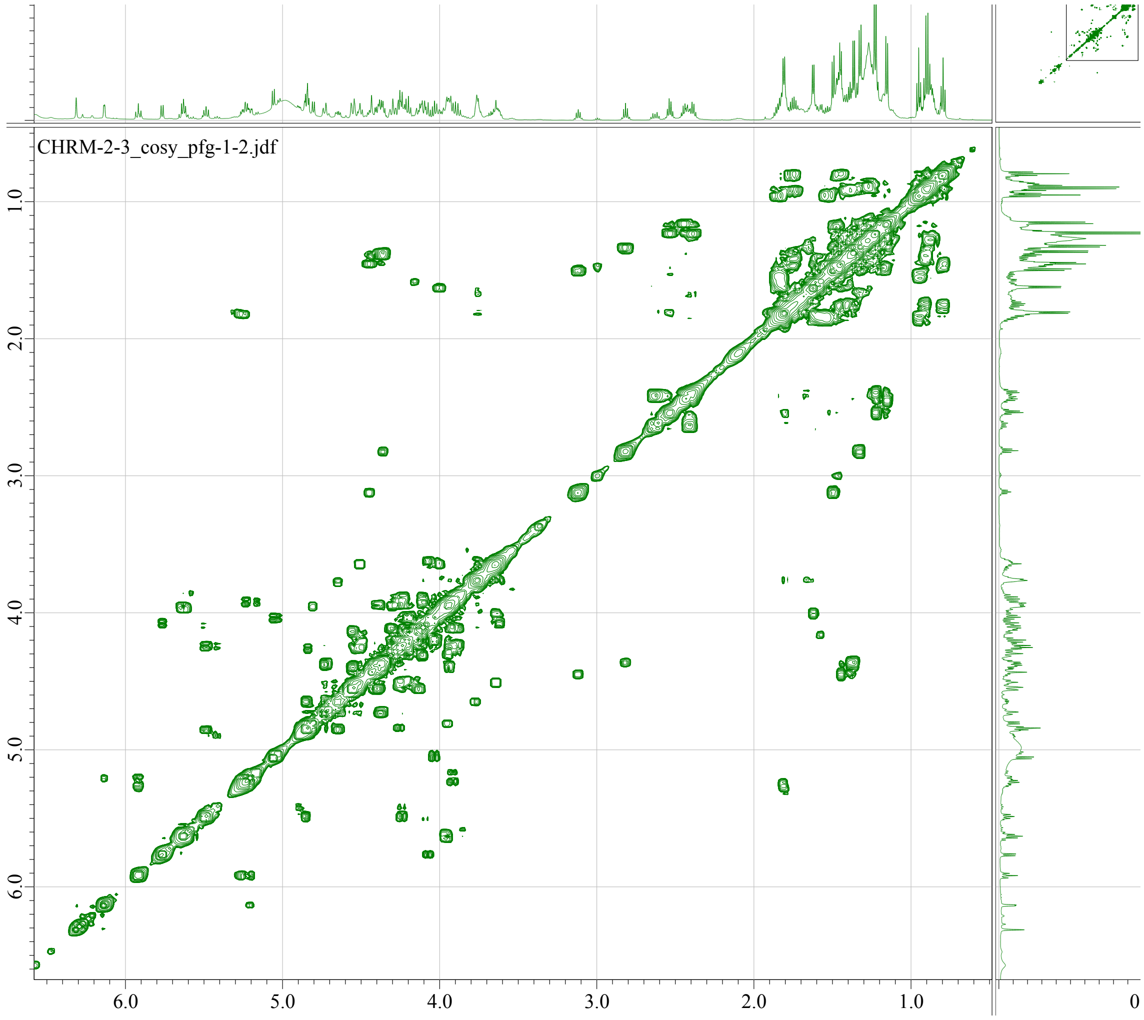

Figure S39: ^{1}H-^{1}H COSY (600 MHz, pyridine-d_5) spectrum of **6**

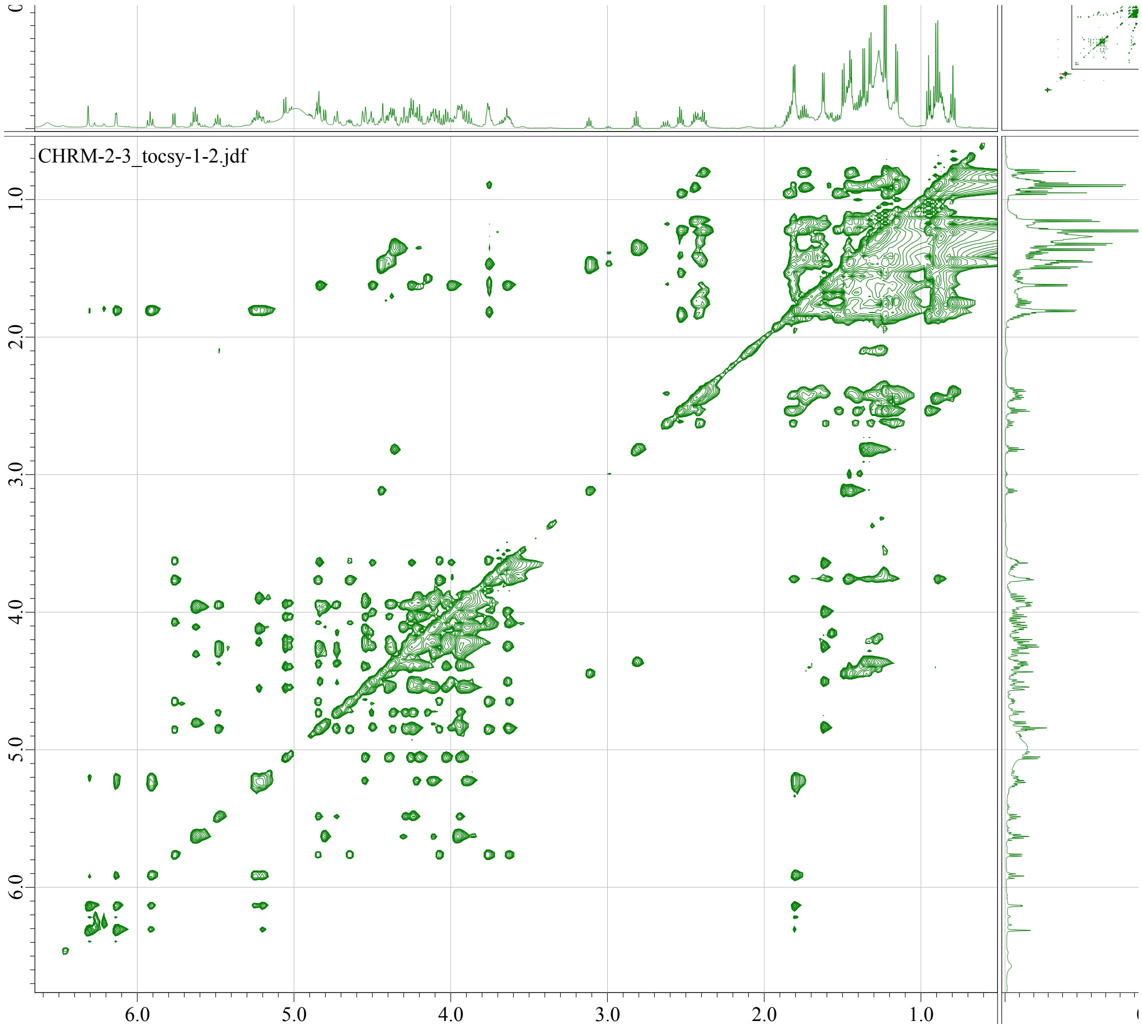

Figure S40: ^{1}H-^{1}H TOCSY (600 MHz, pyridine-d_5) spectrum of **6**

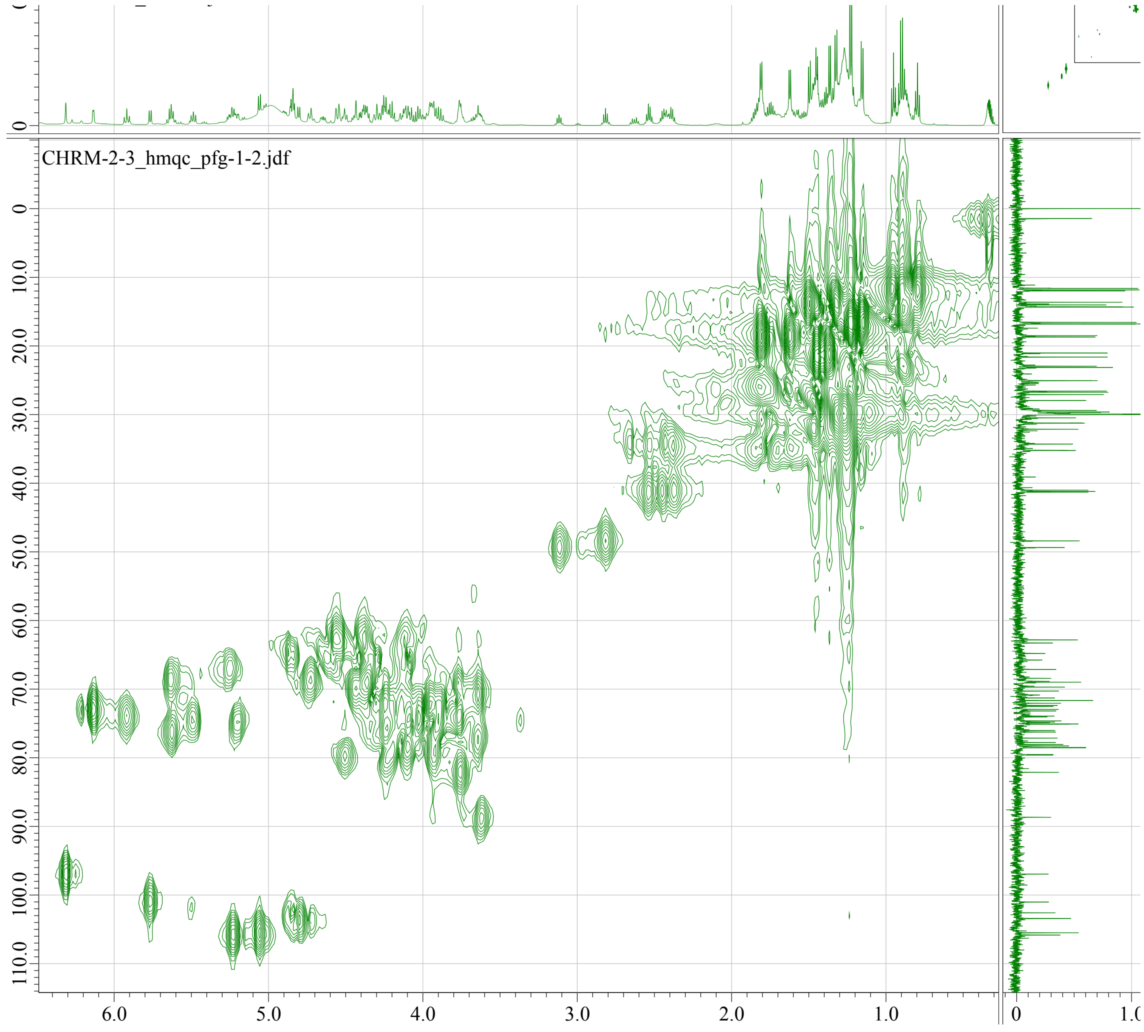

Figure S41: HMQC (600 MHz, pyridine-d_5) spectrum of **6**

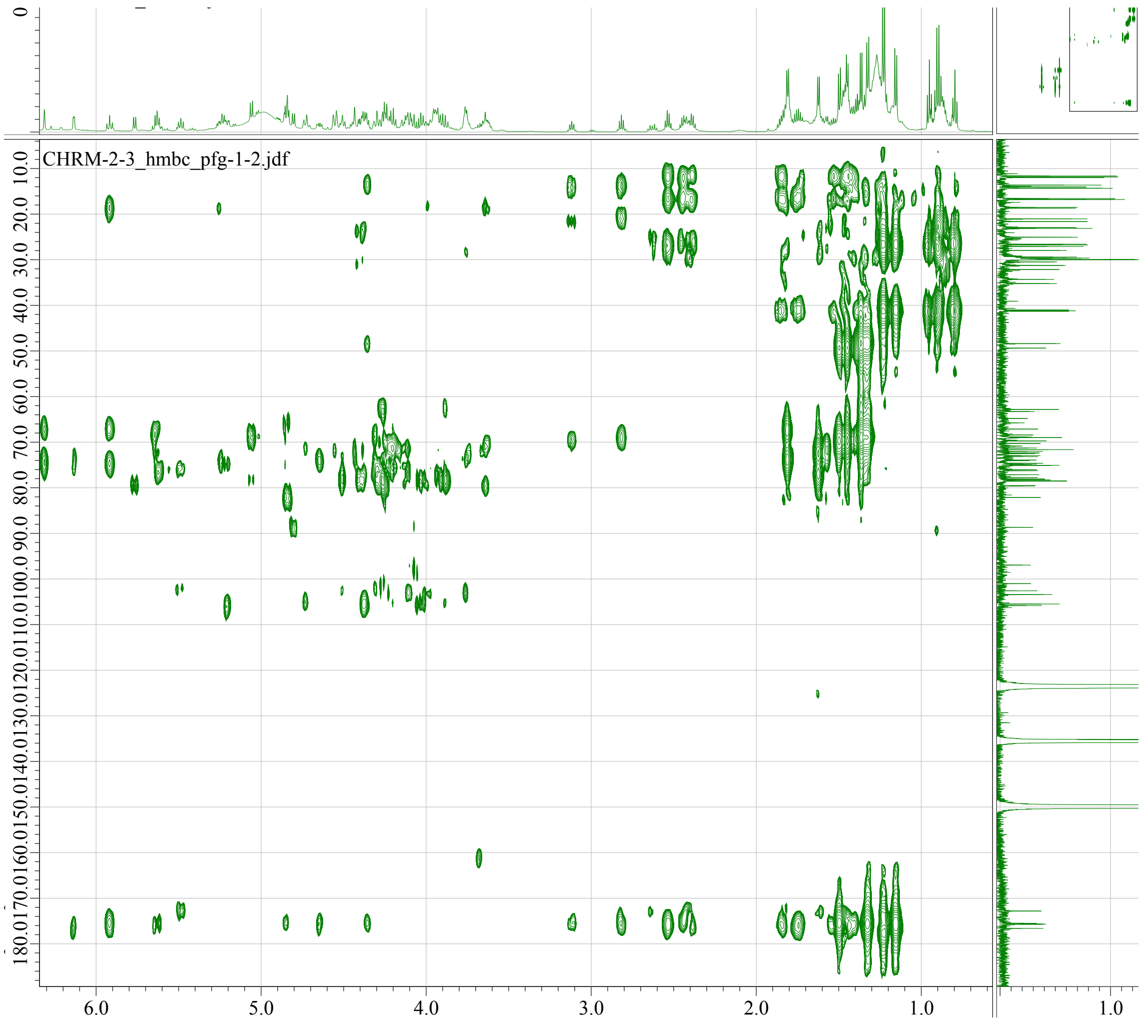

Figure S42: HMBC (600 MHz, pyridine-d_5) spectrum of **6**

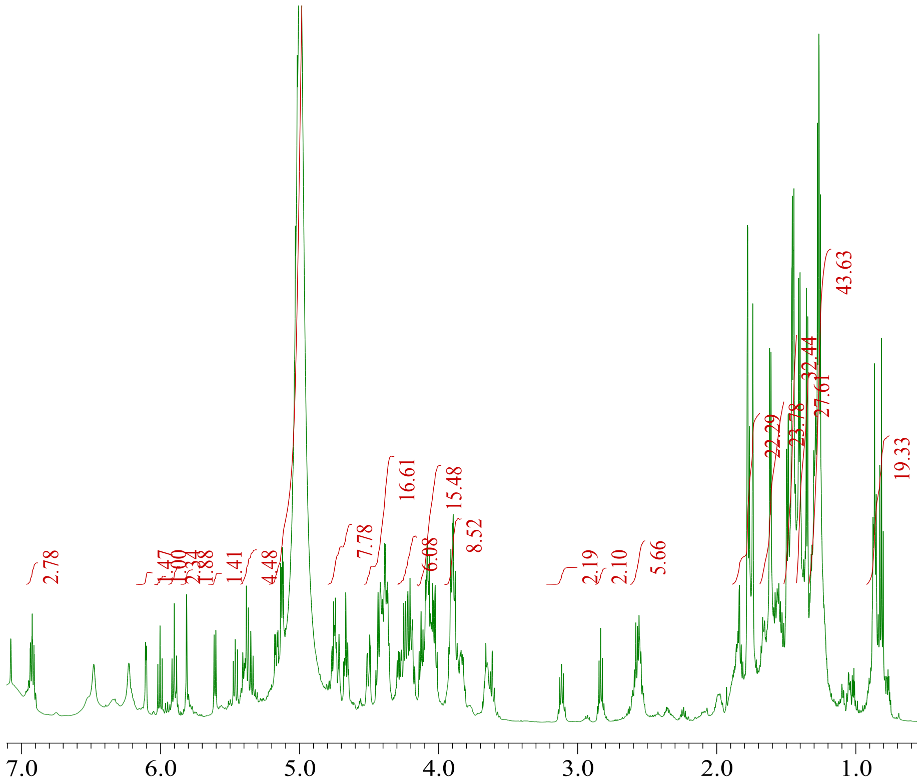

Figure S43: ^{1}H-NMR (600 MHz, pyridine-d_5) spectrum of **7**

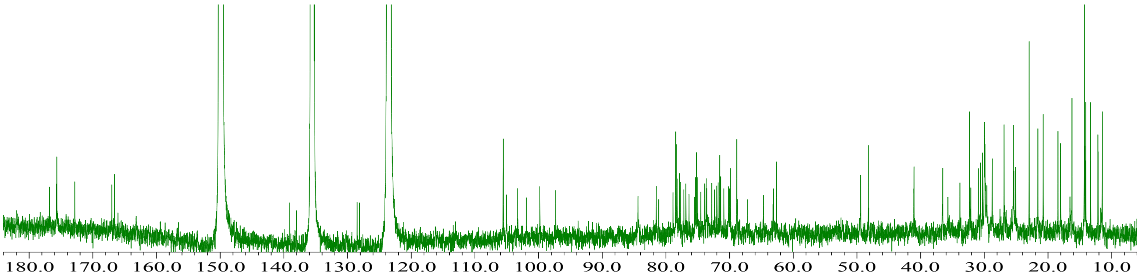

Figure S44: ^{13}C-NMR (150 MHz, pyridine-d_5) spectrum of **7**

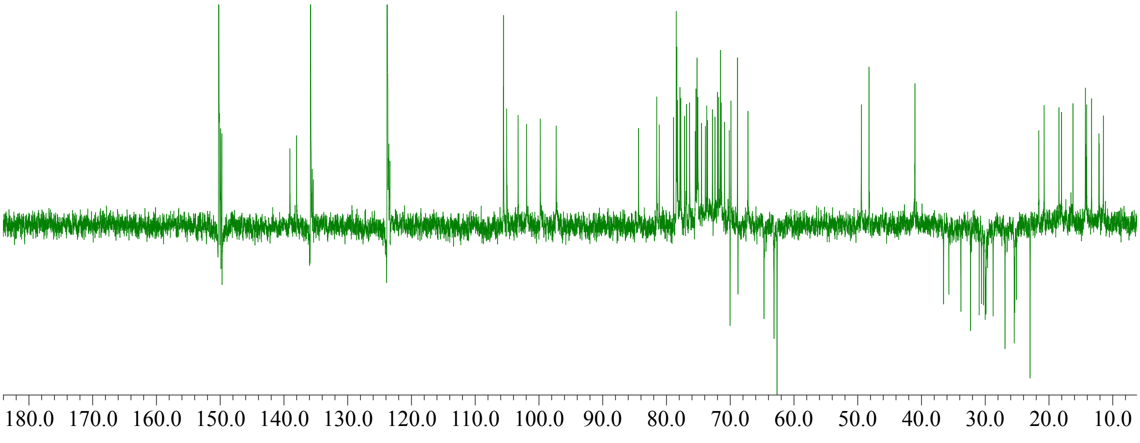

Figure S45: DEPT (150 MHz, pyridine-d_5) spectrum of **7**

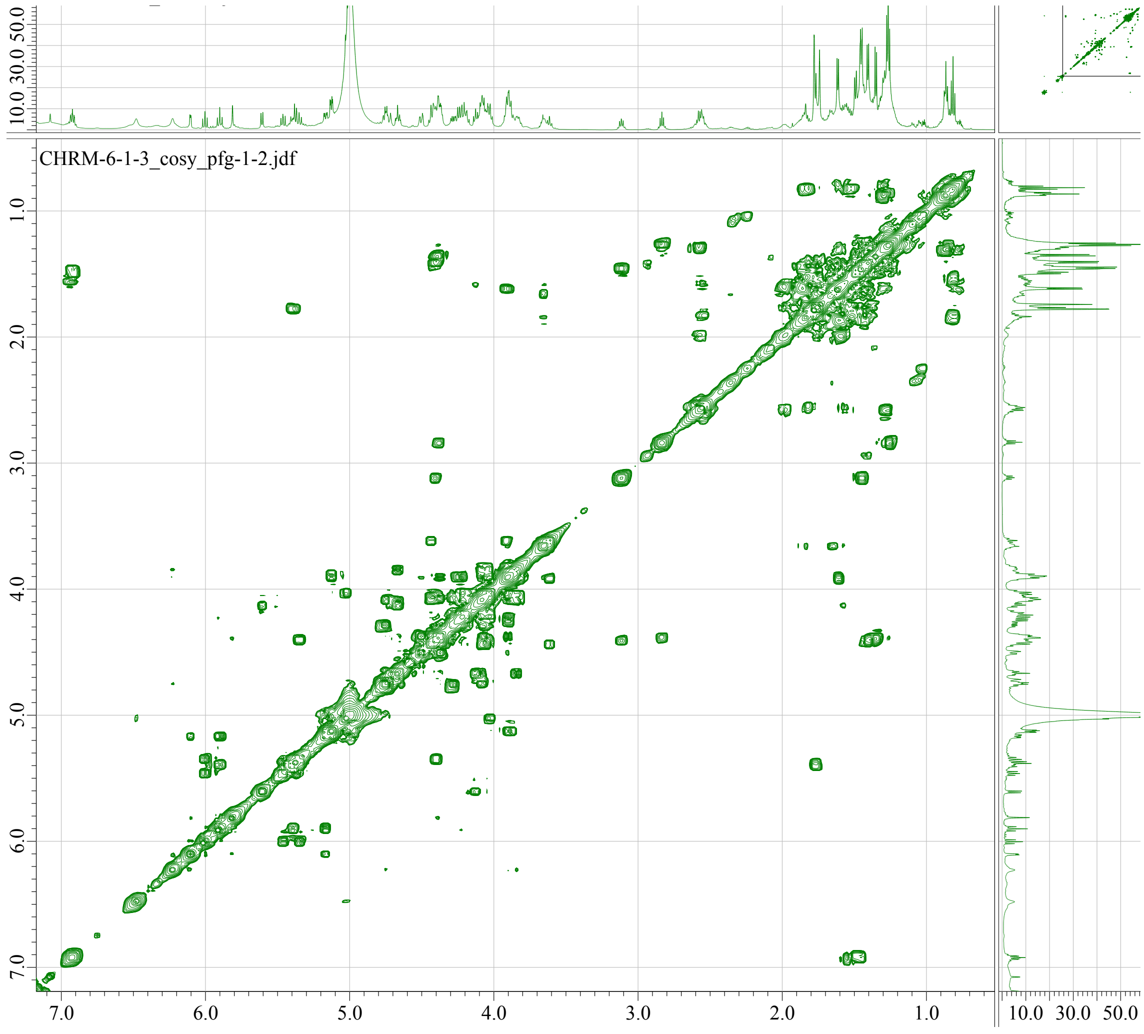

Figure S46: ^{1}H-^{1}H COSY (600 MHz, pyridine-d_5) spectrum of **7**

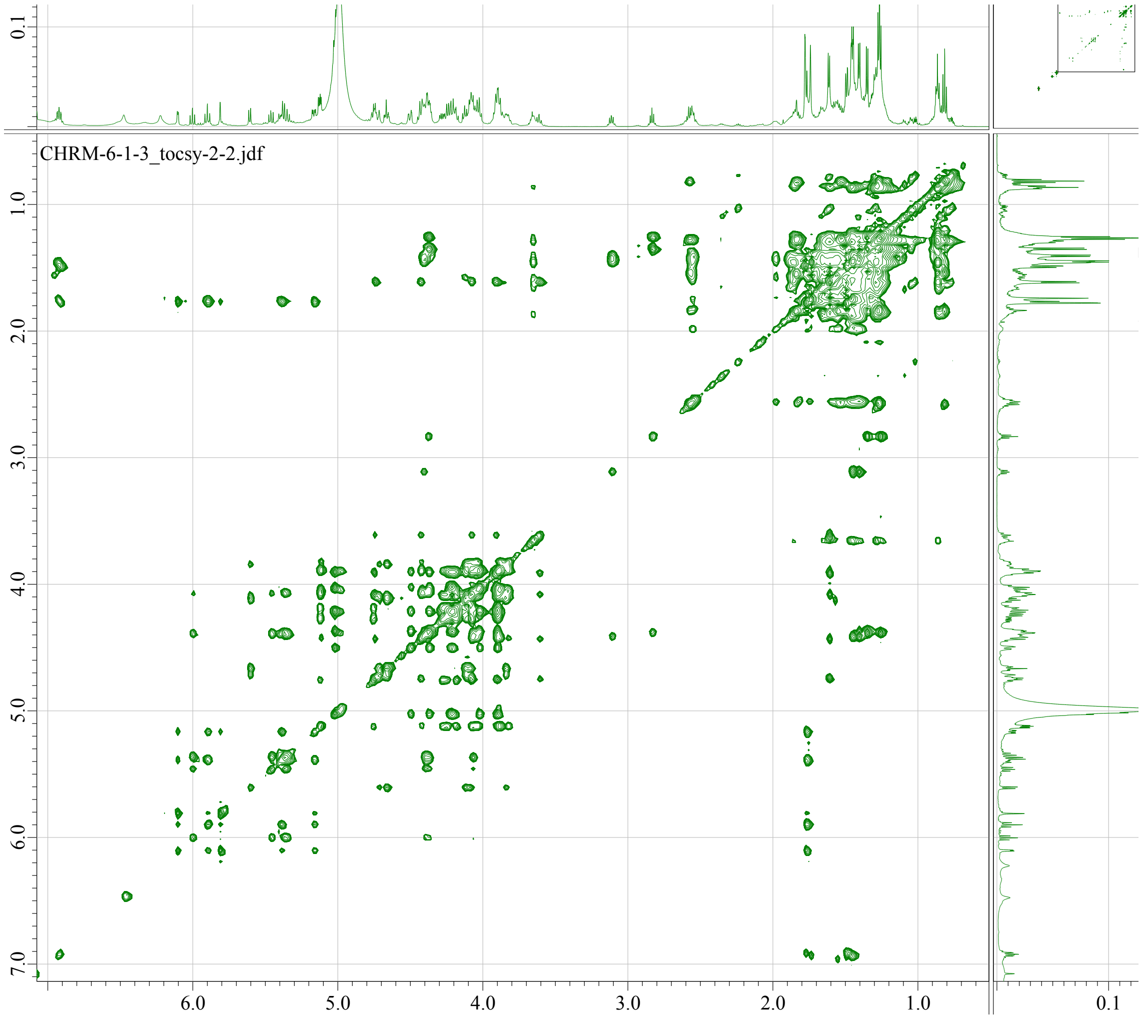

Figure S47: ^{1}H-^{1}H TOCSY (600 MHz, pyridine-d_5) spectrum of **7**

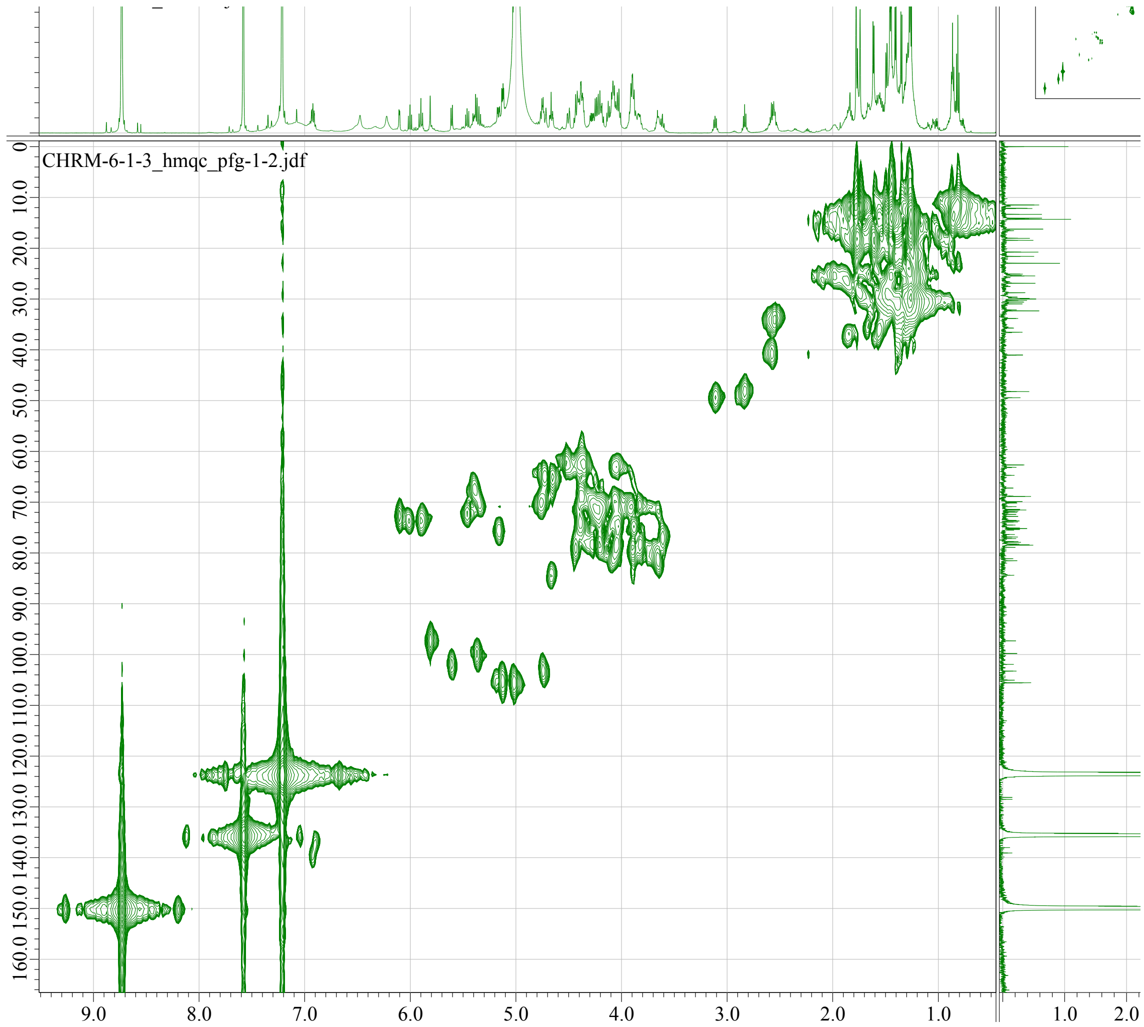

Figure S48: HMQC (600 MHz, pyridine-d_5) spectrum of **7**

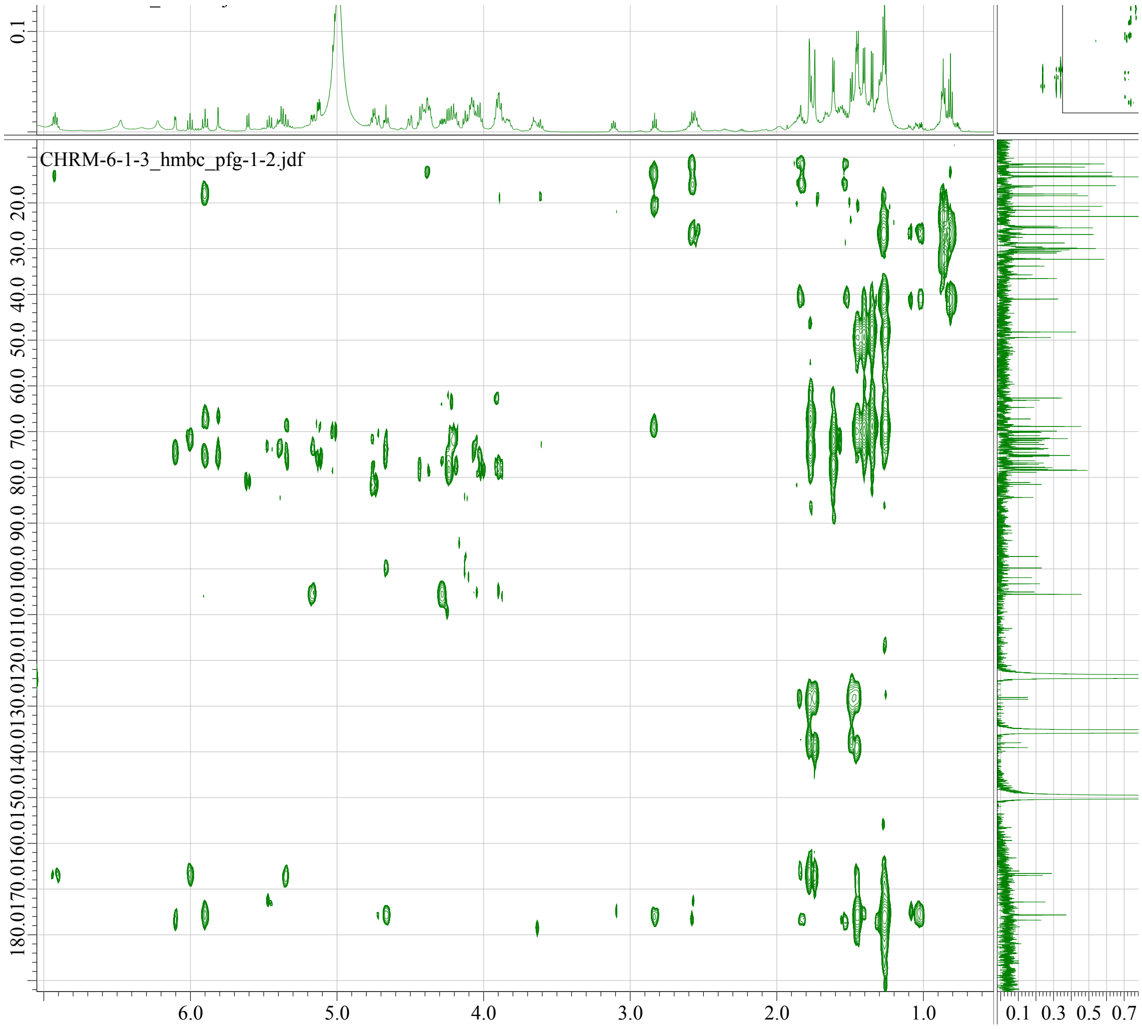

Figure S49: HMBC (600 MHz, pyridine-d_5) spectrum of **7**

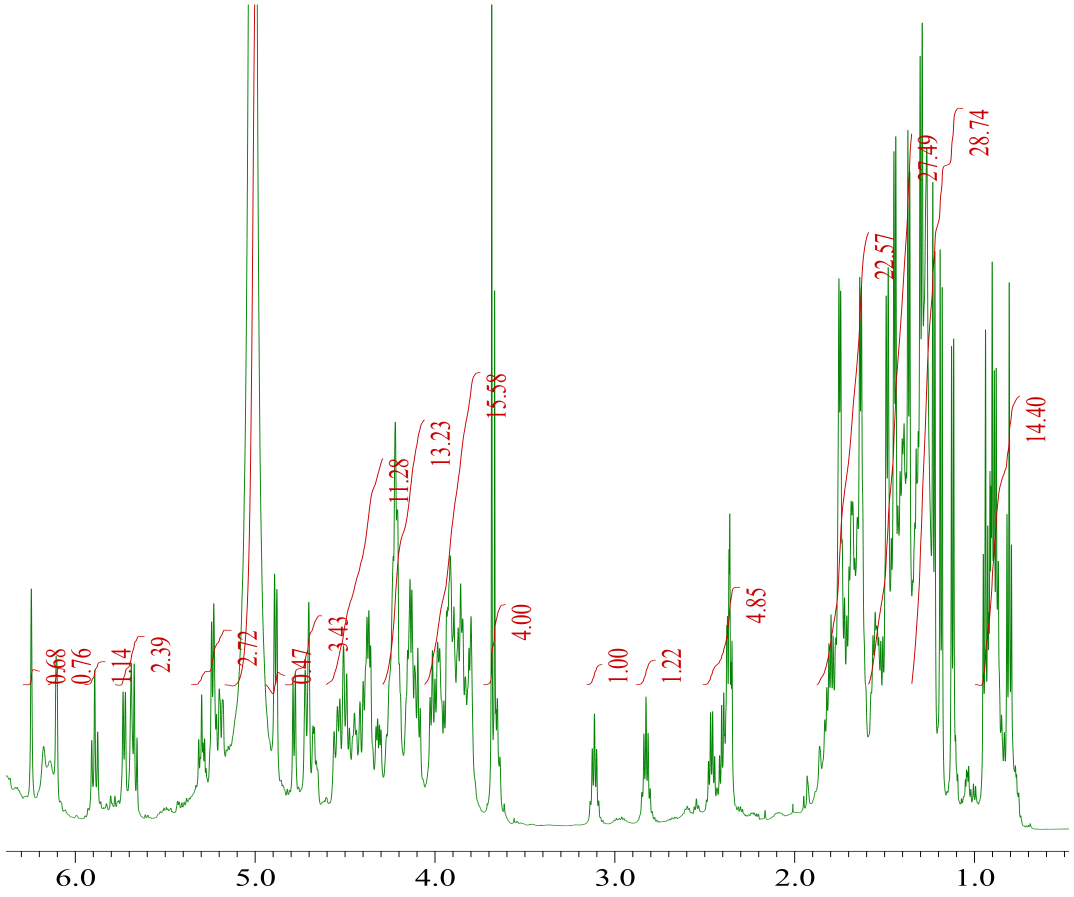

Figure S50: ¹H-NMR (600 MHz, pyridine-d_5) spectrum of **8**

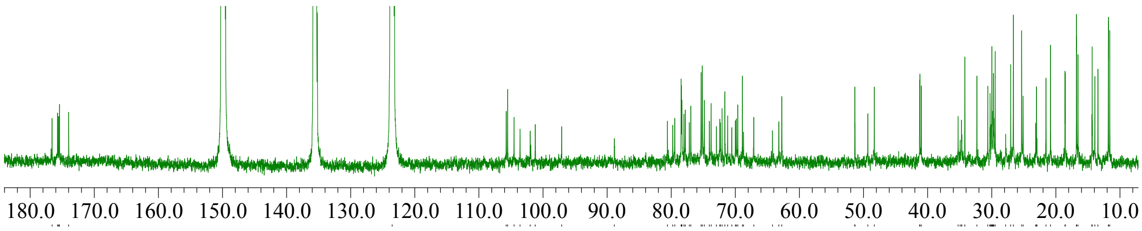

Figure S51: ¹³C-NMR (150 MHz, pyridine-d_5) spectrum of **8**

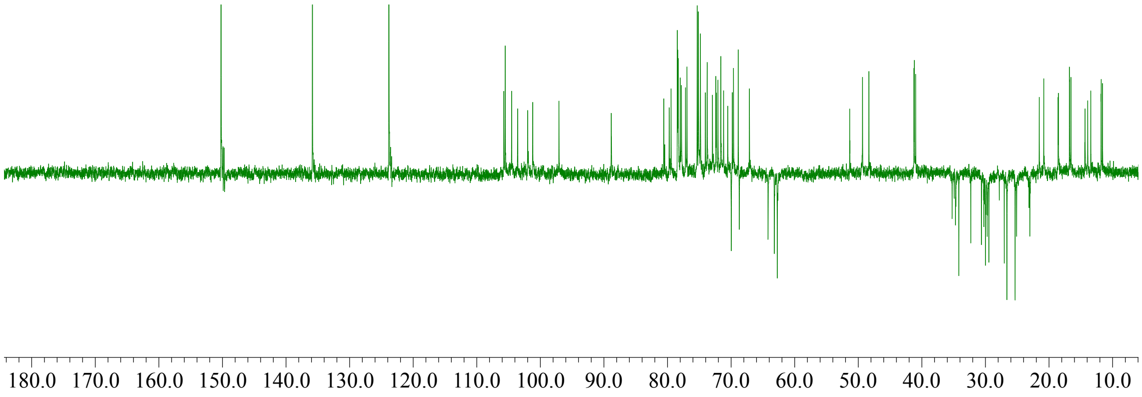

Figure S52: DEPT (150 MHz, pyridine-d_5) spectrum of **8**

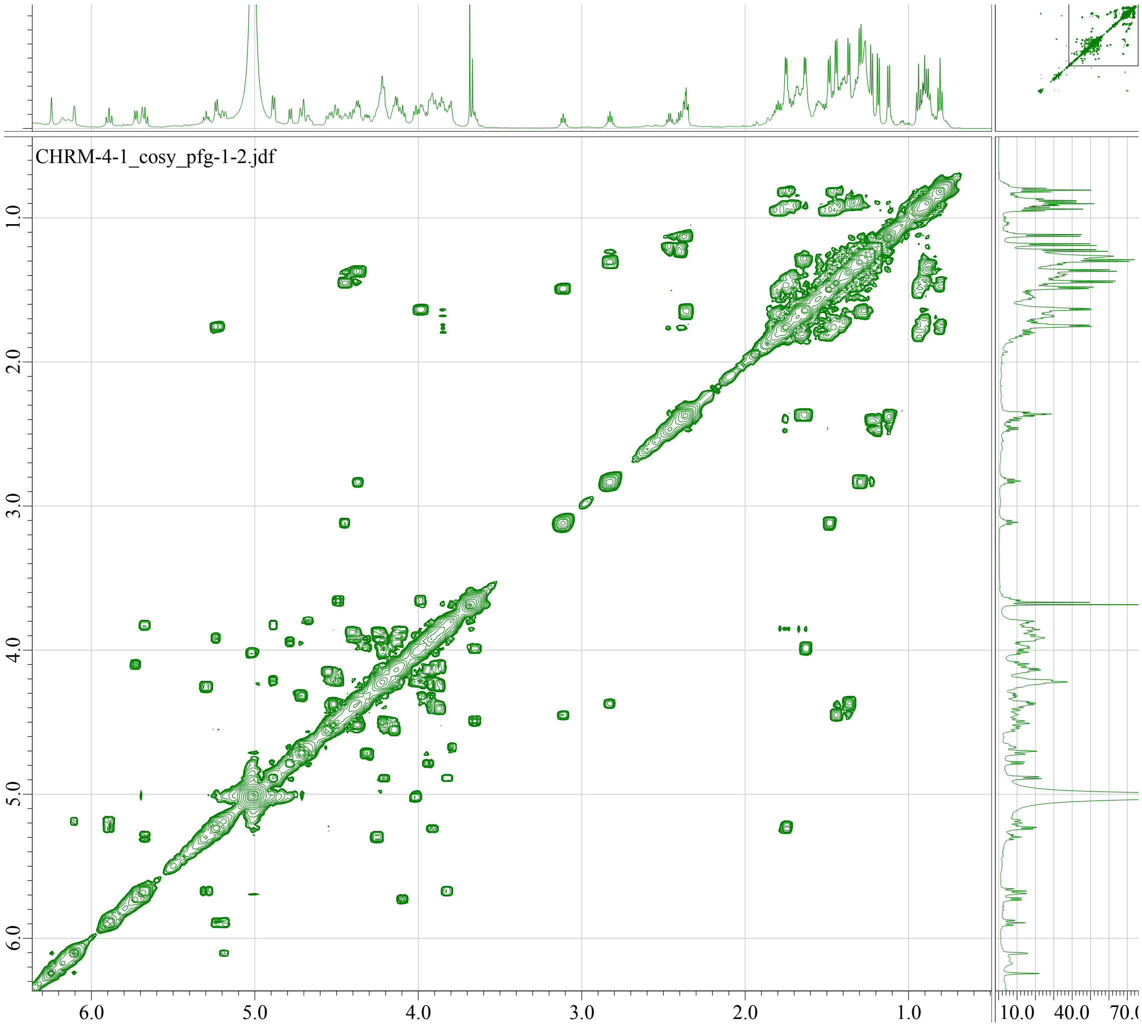

Figure S53: ^{1}H-^{1}H COSY (600 MHz, pyridine-d_5) spectrum of **8**

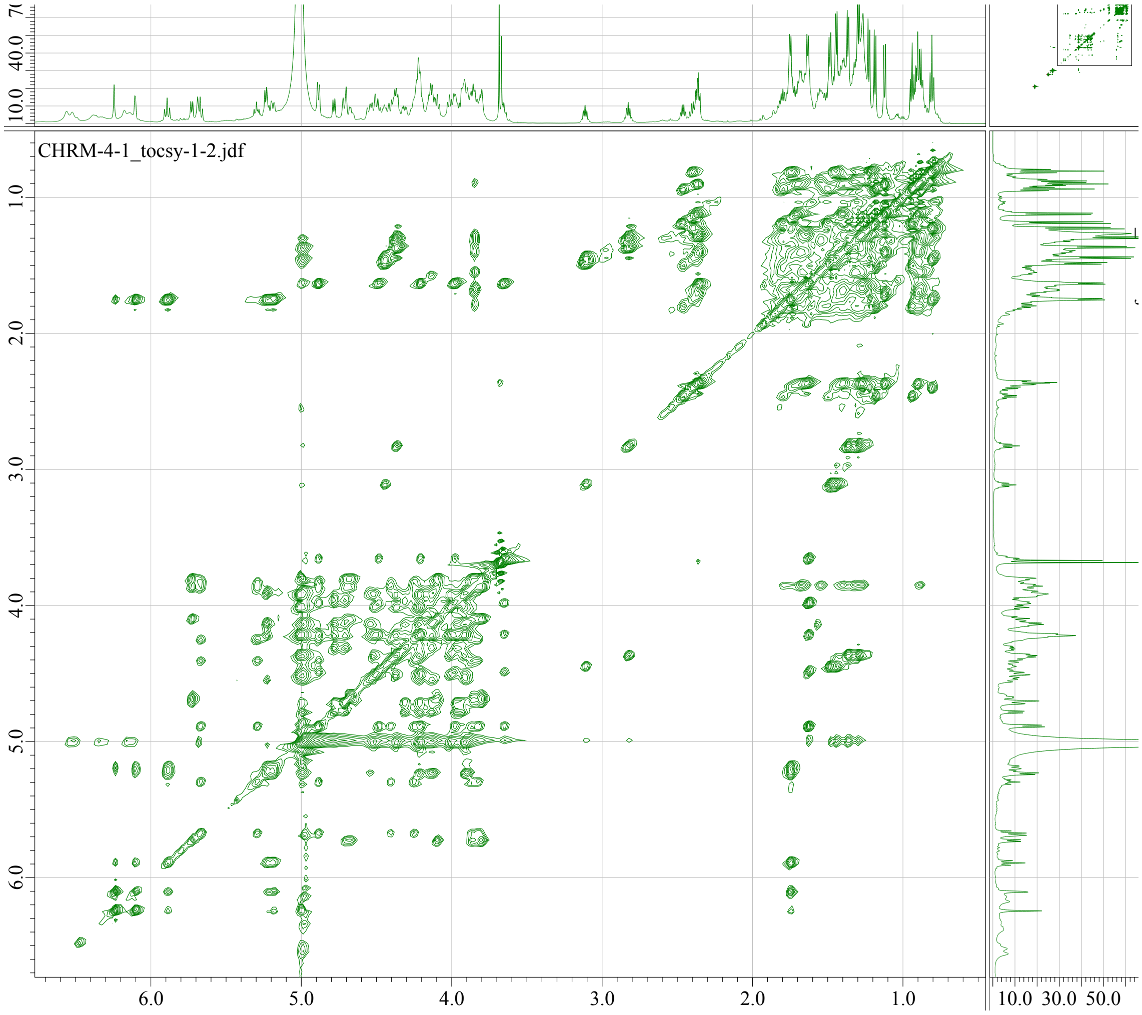

Figure S54: ^{1}H-^{1}H TOCSY (600 MHz, pyridine-d_5) spectrum of **8**

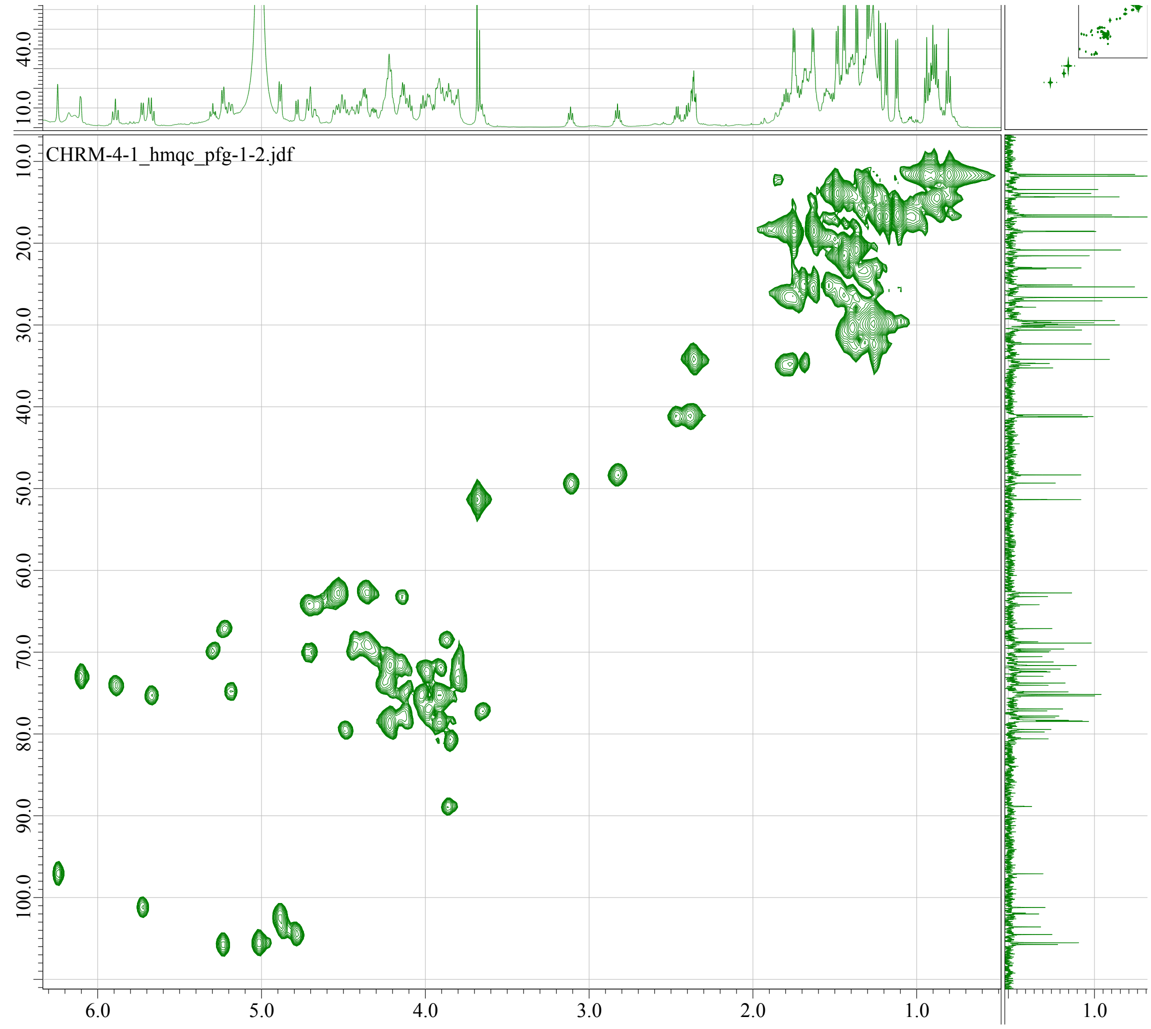

Figure S55: HMQC (600 MHz, pyridine-d_5) spectrum of **8**

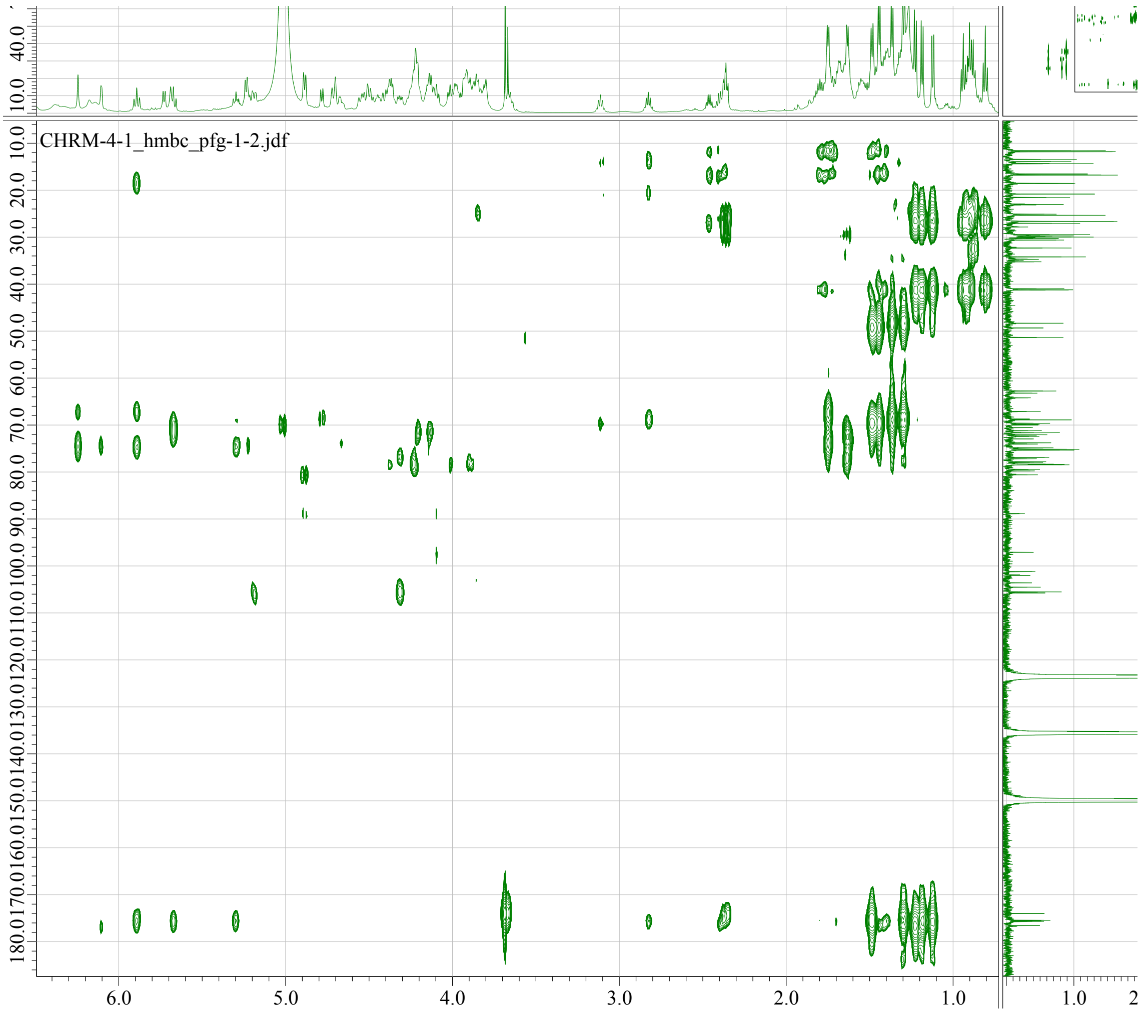

Figure S56: HMBC (600 MHz, pyridine-d_5) spectrum of **8**
